# Synthesis and Evaluation of the Tetracyclic Ring-System of Isocryptolepine and Regioisomers for Antimalarial, Antiproliferative and Antimicrobial Activities

**DOI:** 10.3390/molecules26113268

**Published:** 2021-05-30

**Authors:** Katja S. Håheim, Emil Lindbäck, Kah Ni Tan, Marte Albrigtsen, Ida T. Urdal Helgeland, Clémence Lauga, Théodora Matringe, Emily K. Kennedy, Jeanette H. Andersen, Vicky M. Avery, Magne O. Sydnes

**Affiliations:** 1 Department of Chemistry, Bioscience and Environmental Engineering, University of Stavanger, NO-4036 Stavanger, Norway; katja.s.haheim@uis.no (K.S.H.); emil.lindback@uis.no (E.L.); ida.t.helgeland@uis.no (I.T.U.H.); clemence.lauga08@gmail.com (C.L.); theodora.matringe@gmail.com (T.M.); 2 Discovery Biology, Griffith Institute for Drug Discovery, Griffith University, Don Young Road, Nathan, QLD 4111, Australia; kahni.tan@griffith.edu.au (K.N.T.); emily.kennedy@griffith.edu.au (E.K.K.); v.avery@griffith.edu.au (V.M.A.); 3 The Norwegian College of Fishery Science, Marbio, UiT—The Arctic University of Norway, Breivika, NO-9037 Tromsø, Norway; marte.albrigtsen@uit.no (M.A.); jeanette.h.andersen@uit.no (J.H.A.); 4 CRC for Cancer Therapeutics, Griffith University, Nathan, QLD 4111, Australia; 5 School of Environment & Science, Nathan, Griffith University, Nathan, QLD 4111, Australia

**Keywords:** indoloquinoline, antiplasmodial activity, antiproliferative activity, antimicrobial activity, biofilm inhibition

## Abstract

A series of novel quinoline-based tetracyclic ring-systems were synthesized and evaluated in vitro for their antiplasmodial, antiproliferative and antimicrobial activities. The novel hydroiodide salts **10** and **21** showed the most promising antiplasmodial inhibition, with compound **10** displaying higher selectivity than the employed standards. The antiproliferative assay revealed novel pyridophenanthridine **4b** to be significantly more active against human prostate cancer (IC_50_ = 24 nM) than Puromycin (IC_50_ = 270 nM) and Doxorubicin (IC_50_ = 830 nM), which are used for clinical treatment. Pyridocarbazoles **9** was also moderately effective against all the employed cancer cell lines and moreover showed excellent biofilm inhibition (**9a**: MBIC = 100 µM; **9b**: MBIC = 100 µM).

## 1. Introduction

Malaria and cancer are two major health issues affecting millions of lives annually. Malaria is a parasitic blood disease caused by protozoans of the *Plasmodium* genus. Although five *Plasmodium* strains are known to infect humans, namely *P. falciparum*, *P. vivax*, *P. ovale*, *P. knowlesi* and *P. malariae*, infections by *P. falciparum* are responsible for the majority of malaria-related deaths [1,2]. The World Health Organization (WHO) estimated the number of malaria cases to be 229 million in 2019, claiming approximately 409,000 lives [1], despite considerable global efforts to combat the disease. A major obstacle in the battle against malaria has been the rapid appearance and spread of resistant strains across endemic areas [3]. An excess of 90% of all malaria incidents occur in sub-Saharan Africa [1], a region sorely dependent on the availability of affordable treatments. Originally, malaria-endemic regions were primarily limited to the immediate areas surrounding the tropics. The increasing surface air temperatures as a consequence of global warming is predicted to change this, leaving also temperate climates susceptible to the disease, and with it, a larger part of the human population [4]. Following the widespread appearance of chloroquine (CQ)-resistant strains of *P. falciparum*, artemisinin-based therapies have been the gold standard of malaria treatment [5]. However, in 2008, the first reports of artemisinin-based resistance were observed in Cambodia [6] and ten years later, over 30 independent cases had been documented throughout southeast Asia [7]. Therefore, the development of novel and affordable treatments remains of paramount importance. 

Contrary to malaria, which is an infectious disease, cancer is a noncommunicable disease, ranking as the second leading cause of death globally, responsible for approximately 1 in 6 deaths. Estimates from WHO put the number of cancer cases in 2018 at 18.1 million, accompanied by 9.6 million fatalities [8]. The five most diagnosed cancers are lung, breast, colorectal, prostate and stomach. A variety of anti-cancer therapies are currently available, however, those treated suffer from the unwanted side effect of being highly immunosuppressed. Patients suffering from a compromised immune system following cancer treatments are therefore more likely to contract nosocomial infections [9], such as infection caused by drug-resistant *Staphylococcus aureus*, increasing the overall burden of nosocomial infectious diseases [10]. This is further complicated by the increased likelihood of formations of multidrug-resistant biofilms, which are notoriously hard to treat [11,12]. Bacterial infections are also known to be a cause of cancer on their own, and according to the WHO, roughly 13% of all cancers globally occur as a result of chronic infections [8]. Additionally, research in recent years has started to uncover a direct link between the formation of microbial biofilms in the body and the growth of certain cancers [13,14,15]. The availability of anticancer drugs with the dual capability of inhibiting biofilm growth is severely limited, making the development of such drugs greatly needed.

Natural products have proven to be an invaluable source of lead compounds for medicinal research in the past and present due to their wide array of structural diversity [16,17,18]. As of 2020, roughly 40% of all Food and Drugs Administration (FDA) approved drugs have natural origins [19], further demonstrating the importance of natural products in drug discovery. Accordingly, discovery and characterization of natural products and their semi-synthetic derivatives remain pivotal in the search for novel drug candidates [20]. The quinoline core represents a versatile structural motif, possessing applications in the fields of material science, the dye industry and moreover constitute an important building block in the design of pharmaceutical compounds [21]. In particular, due to the presence of the quinoline skeleton in numerous natural products displaying a vast array of biological activities, quinoline-based natural products and their derivatives are attractive medicinal targets [22,23,24,25].

**Figure 1:**
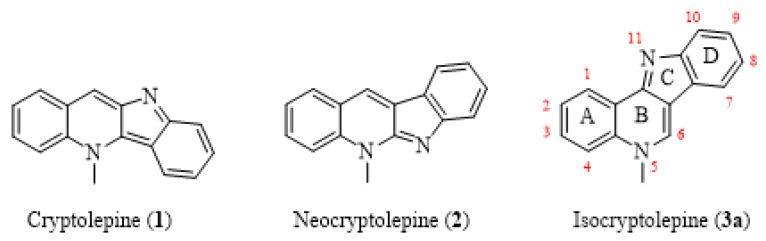
Structures of the major bioactive compounds isolated from *Cryptolepis Sanguinolenta*; cryptolepine (**1**), neocryptolepine (**2**) and isocryptolepine (**3a**).

Almost exclusively found in the West African climbing shrub *Cryptolepis sanguinolenta* [26,27], the indoloquinoline natural products cryptolepine (**1**), neocryptolepine (**2**), and isocryptolepine (**3a**) (Figure 1) represent a unique class of bioactive compounds. These alkaloids are characterized by a fused quinoline and indole moiety [28] and long before the constituents of *C. sanguinolenta* were identified, the extracts were used in herbal remedies to treat malarial fevers among other ailments [29]. The major bioactive component of the shrub was eventually determined to be cryptolepine (**1**), which has subsequently received the most attention in the literature of the three regioisomeric indoloquinolines **1**, **2**, and **3a**. A host of biological properties have been observed in cryptolepine (**1**) assays, such as antiplasmodial, antimalarial [30,31,32,33,34,35], anti-inflammatory [33], antifungal [36,37,38], antimicrobial [39,40,41,42], antiproliferative [43,44,45,46] and antiviral [40]. The linearly arranged planar structure of cryptolepine (**1**) is believed to be related to its high level of undesired cytotoxicity, resulting in its ability to non-specifically intercalate into DNA, inhibiting topoisomerase II [44,47,48,49]. Neocryptolepine (**2**) and isocryptolepine (**3a**) have also been demonstrated to possess similar biological profiles, although inferior to cryptolepine (**1**) [28,50]. Despite the lower potency, both neocryptolepine (**2**) and isocryptolepine (**3a**) were revealed to be significantly less cytotoxic than cryptolepine (**1**), allowing for the possibility of their derivatives to be developed into new lead compounds [49,51].

**Figure 2:**
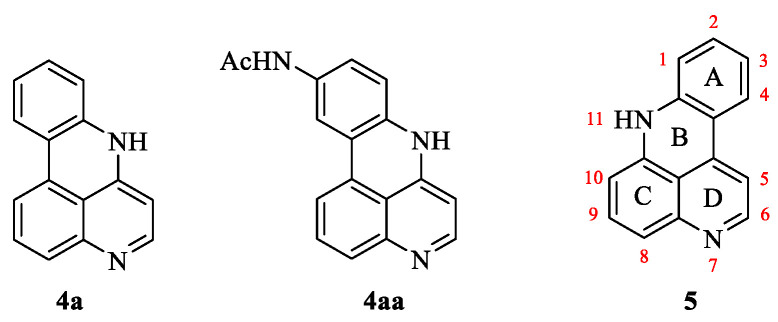
Structures of pyridophenanthridines **4a** and **4aa** along with pyridoacridine **5**.

The biological activities for the core structures of *C. sanguinolenta* have been extensively studied while their regioisomers have been largely undescribed. In particular, the novel pyridophenanthridine scaffold **4a** (Figure 2) unveiled in our previous study [52], represents an interesting target for biological evaluation. The pyridophenanthridine skeleton may be regarded as a regioisomer to the pyridoacridines (the core structure of which is illustrated in compound **5** in Figure 2), a well-studied class of marine alkaloids most notably known for exhibiting potent antiproliferative qualities [53,54,55,56,57]. Similarly, to cryptolepine (**1**), nearly all naturally occurring pyridoacridines have been shown to act as DNA intercalating agents, resulting in cytotoxic effects in cultured tumor cells [54,55,58]. They also possess the ability to inhibit topoisomerase II [53,58] and further contain biological profiles such as antibacterial, antifungal, antiviral, antiparasitic and insecticidal [53,56,57,59,60,61]. Consequently, it is postulated that the pyridoacridines and their synthetic derivatives are pivotal for the future generation of medicinal compounds [58]. 

Recently, we described the preparation of several isocryptolepine regioisomers and certain chemoisomers [52]. In this paper, we present modifications to our previous synthetic strategies which allowed for the realization of novel tetracyclic ring-systems (compounds **4b**, **8**, and **9**) along with the *N*-alkylation of several compounds to furnish new analogues (compounds **3b**, **3c**, **10**, and **21**). Moreover, the newly synthesized compounds, along with our existing library of natural products and analogues, were evaluated for their in vitro antiplasmodial activity against *Plasmodium falciparum* 3D7 parasites; cytotoxicity against normal mammalian cell line (HEK293), and three cancer cell lines (HCT116, MDA-MB-231 and PC-3). The compounds were also evaluated as antimicrobial agents against common pathogenic bacteria as well as their ability to inhibit biofilm growth. 

## 2. Results and Discussion

### 2.1. Chemistry

**Scheme 1:**
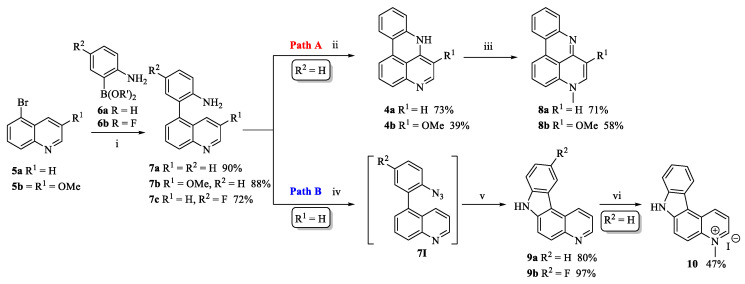
Synthesis of pyridophenanthridines **8** and pyridocarbazoles **9** from a common starting material. Conditions: (i) coupling partner **6**, Cs_2_CO_3_, Pd(PPh_3_)_4_ (5 mol%), DME/H_2_O, 80 °C; Path A: (ii) PdCl_2_ (dppf) (20 mol%), IMes (5 mol%), H_2_O_2_ (35 wt%, 29 mol%), AcOH, 118 °C, MW; (iii) CH_3_I, CH_3_CN, reflux; Path B: (iv) 1) HCl (37%), NaNO_2_ (0.4 M), 0 °C, 1.5 h; 2) NaN_3_/NaOAc, 0 °C, 1 h; (v) 1,2-dichlorobenzene, 180 °C, 3 h; (vi) CH_3_I, CH_3_CN, reflux.

We recently reported a concise synthesis of isocryptolepine (**3a**) and some regioisomers in which the two key synthetic steps were a Suzuki-Miyaura cross-coupling reaction followed by a palladium-catalyzed intramolecular cyclization [52,62]. The most unexpected result of our previous endeavor was the formation of a pyridophenanthridine scaffold **4a**, when biaryl **7a** was treated with palladium under our intramolecular cyclization conditions (Path A, Scheme 1). Shortly after our report, Kumar and co-workers reported the formation of compound **4a** by a palladium-catalyzed arylation technique utilizing diaryliodinium salts [63]. To the best of our knowledge, these two preparations of pyridophenanthridine **4a** remain the only descriptions in the literature. However, Beauchard and coworkers describe the accidental synthesis of the functionalized pyridophenanthridine **4aa** in 2006 (Figure 2) [64]. This was the result of attempting to synthesize isocryptolepine analogues by a microwave-induced thermal decomposition of a benzotriazole-coupled quinoline.

Intrigued by these results, we decided to investigate further and wondered if the regioselectivity would be the same utilizing a different synthetic strategy. Drawing inspiration from Timári et al. [65] in the synthesis of isocryptolepine and further expanded on by Hostyn et al. [66] for the synthesis of isoneocryptolepine, a Suzuki-Miyaura cross-coupling reaction and nitrene insertion approach was undertaken. Standard azidation of biaryl **7a** via installation of a diazonium salt yielded the aryl azide **7I**, which upon thermal decomposition in refluxing 1,2-dichlorobenzene interestingly gave pyridocarbazole **9a** as the only product without any traces of its regioisomer **4a** (Scheme 1, Path B). Thereby, it was concluded that **4a** and **9a** can be achieved from a common starting material by following reaction pathway A and B, respectively, in Scheme 1. A fluoro-substituted analogue of compound **9a**, namely **9b**, was further possible to construct starting from boronic acid **6b**. To conclude the synthetic pathways, compounds **4** and **9a** were finally regioselectively *N*-methylated using excess iodomethane in refluxing acetonitrile [49] to realize tetracycles **8** and **10**. 

**Scheme 2:**
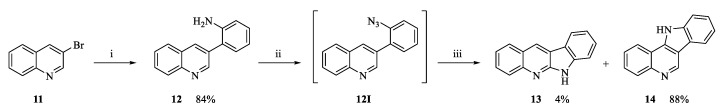
Synthesis of 6*H*-indolo[2,3-*b*] quinoline (**13**) and 11*H*-indolo[3,2-*c*] quinoline (**14**) via a Suzuki-Miyaura cross-coupling and thermally induced nitrene insertion approach. Conditions: (i) boronic acid **6a**, K_2_CO_3_, PdCl_2_ (dppf) (5 mol%), EtOH/H_2_O (5:1), 60 °C [52,62]; (ii) 1) HCl (37%), NaNO_2_ (0.4 M), 0 °C, 1.5 h; 2) NaN_3_/NaOAc, 0 °C, 1 h; (iii) 1,2-dichlorobenzene, 180 °C, 3 h.

**Scheme 3:**
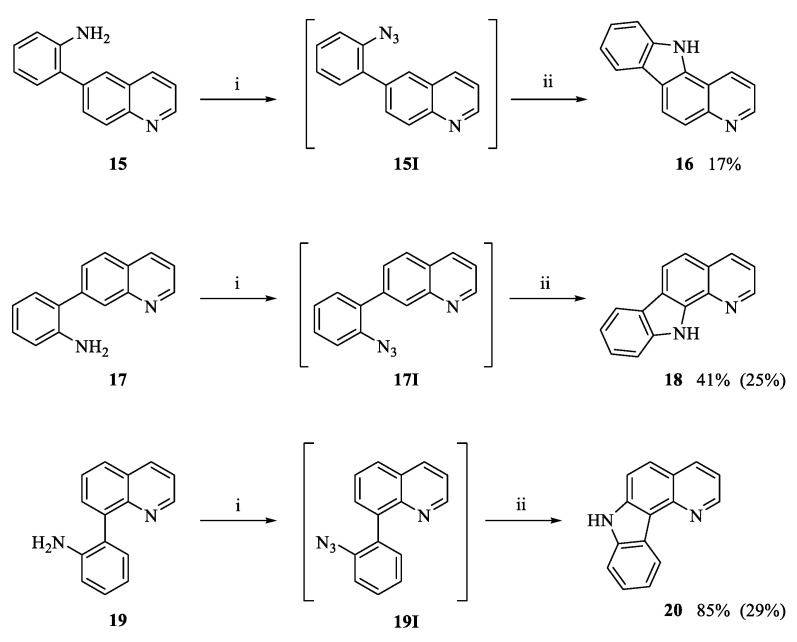
Synthesis of 11*H*-pyrido[3,2-*a*] carbazole (**16**), 11*H*-pyrido[2,3-*c*] carbazole (**18**) and 7*H*-pyrido[3,2-*c*] carbazole (**20**) using a diazotization-azidation-nitrene insertion approach. In brackets: yields from our previous endeavors [52]. Conditions: (i) HCl (37%), NaNO_2_ (0.4 M), 0 °C, 1.5 h; (ii) 1,2-dichlorobenzene, 180 °C, 3 h. For the synthesis of compounds **15**, **17**, and **19**, refer to our previous work [52].

**Scheme 4:**
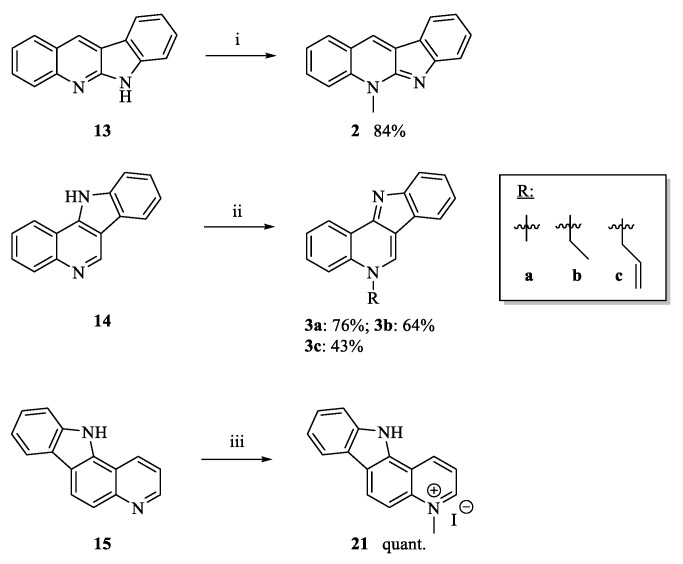
Regioselective *N*-alkylations to synthesize neocryptolepine (**2**), isocryptolepine (**3a**), *N*-alkyl isocryptolepine derivatives (**3b** and **3c**) and 4-methyl-11*H*-pyrido[3,2-*a*]carbazolium iodide (**21**). Conditions: (i) CH_3_CN, THF, reflux, 24 h [67]; (ii) a: CH_3_I, PhMe, reflux, 3 h [62]; b: CH_3_CH_2_I, PhMe, reflux, 3 h; c: CH_2_=CHCH_2_Br, PhMe, reflux, 22 h; (iii) CH_3_I, CH_3_CN, reflux, 20 h.

In Timári et al.’s original synthesis of isocryptolepine (**3a**) by means of a thermally induced nitrene insertion, only one regioisomeric product was observed, namely isocryptolepine precursor **14** [65]. Following the same conditions in our laboratories, the approach primarily resulted in the construction of indoloquinoline **14** but its regioisomer **13** was also formed in minor quantities (Scheme 2). Applying the nitrene insertion approach to biaryls **15**, **17**, and **19**, we were able to significantly improve the yields of tetracycles **18** and **20** compared to our previous endeavors (Scheme 3, previous yields in brackets) [52]. Following a literature procedure, neocryptolepine (**2**) was obtained in good yield starting from its precursor **13** (Scheme 4) [67].

Modification of our previously reported conditions for the *N*-methylation of tetracycle **14** to furnish isocryptolepine (**3a**) [62], allowed the formation of two novel isocryptolepine analogues **3b** and **3c**, albeit in lower yields than the parent alkaloid (Scheme 4). Of the remaining tetracycles, namely compounds **16**, **18**, and **20**, only compound **16** was successfully *N*-methylated using the same conditions as reported in our previous work [62]. Efforts to explain the failure of tetracycles **18** and **20** to undergo *N*-alkylation at the most reactive ring-nitrogen, presumably the quinoline moiety, is currently under way in our laboratories.

### 2.2. Antiplasmodial Assay

The prepared natural products and their derivatives were evaluated for their in vitro antiplasmodial activities against the *Plasmodium falciparum* 3D7 strain. The compounds were further tested for their in vitro cytotoxicity against HEK293 cells (human embryonic kidney cells) for the determination of their selectivity indices. Furthermore, to serve as positive controls for our analyses, chloroquine (CQ), dihydroartemisinin (DHA) and puromycin were employed. Results from these studies are summarized in Table 1. 

The tested compounds were found to possess diverse activities against the P*f*3D7 cell line. Albeit being well documented to have antiplasmodial activity in the literature, the parent alkaloid neocryptolepine (**2**) has thus far not been evaluated for in vitro antimalarial activity against P*f*3D7 (IC_50_ = 7249 nM), showing a lower potency compared to isocryptolepine (**3a**) (IC_50_ = 1211 nM). Out of the two novel isocryptolepine derivatives, allyl variant **3c** showed a marginal improvement compared to the natural product (IC_50_ = 1198 nM), while ethyl variant **3b** showed a lower activity (IC_50_ = 1318 nM). Both derivatives were revealed to be notably more cytotoxic than the parent alkaloid **3a**.

The neocryptolepine precursor **13** was revealed to display no antiplasmodial inhibition, which is in accordance with a previous study conducted by Jonckers et al., where they highlighted the importance of the *N*-5 methyl group for activity in certain halogen-substituted indolo[3,2-*b*]quinolines [68]. The regioisomer **15** was also shown to be inactive against P*f*3D7. Contrary to this, the isocryptolepine precursor **14** displayed more potent antimalarial activities (IC_50_ = 977 nM) compared to the parent alkaloid **3a**. For the isocryptolepine precursor **14**, it has been shown through previous work that by introduction of certain basic side chains at C-9, the in vitro antimalarial activity against the K-1 strain of *P. falciparum* was dramatically increased compared to isocryptolepine (**3a**). The authors argued that these observations could be attributed to the basic properties allowing the compound to experience a lower degree of hydrophobicity [69], a quality also observed for CQ [28].

Pyridophenanthridines **4** (a: IC_50_ = 548 nM; b: IC_50_ = 866 nM) outperformed both neocryptolepine (**2**) and isocryptolepine (**3a**) in terms of activity and selectivity; however, it displayed an unfavorable increase in cytotoxicity. Keeping in mind the effects observed by Jonckers et al. [68] for the functionalization of the isocryptolepine precursor **14**, addition of appropriate substituents to pyridophenanthridine **4a** could potentially result in increased antiplasmodial activity. Evidently, the presence of the methoxy substituent in compound **4b** negatively impacted both the antiplasmodial activity and cytotoxicity compared to the naked pyridophenanthridine **4a**. Interestingly, the addition of an *N*-methyl group to pyridophenanthridines **4** to furnish compounds **8** (a: IC_50_ = 1698 nM; b: IC_50_ = 1546 nM) negatively impacts the antiplasmodial activity. For the indoloquinoline natural products, the *N*-methyl group is considered an instrumental aspect for their parasitic inhibition [28], this is evidently not the case for the pyridophenanthridines, possibly suggesting the presence of a novel mode of action against the parasitic life cycle. As this represents the first case in the literature of the antiplasmodial evaluation of a pyridophenanthridine, other functionalizations of the core scaffold should nonetheless be further researched.

The two most prominent results of our studies were the novel hydroiodide salts **10** (IC_50_ = 128 nM) and **21** (IC_50_ = 380 nM), where the latter showed improved selectivity compared to the standards. Their precursors **9a** and **15** showed little to no activity, highlighting the importance of the *N*-methyl functionality. These results are possibly aided by the fact that the salt structure likely promotes increased solubility in aqueous media, further increasing the biological availability of the compounds, a fact which should be carefully considered when exploring new lead compounds.

### 2.3. Antiproliferative Assay

All prepared samples were evaluated in vitro against a panel of three cancer cell lines, including HCT116 (human colon cancer), MDA-MB-231 (human breast adenocarcinoma) and PC-3 (human prostate cancer) using a resazurin assay. Puromycin and Doxorubicin were employed as positive controls for the obtained IC_50_ results, which are summarized in Table 2.

Both parent alkaloids neocryptolepine (**2**) and isocryptolepine (**3a**) performed best against the HCT116 cell line (**2**: 6218 nM; **3a**: 667 nM) (Table 2). It is evident that isocryptolepine (**3a**) had an overall better performance against the tested cancer cell lines than neocryptolepine (**2**). The same was observed for the isocryptolepine derivatives **3b** and **3c**; however, the potency was less than for the parent isocryptolepine (**3a**). Derivatives **3b** and **3c** were revealed to become less potent with increasing alkyl chain length for the human colon cancer (**3b**: IC_50_ = 742 nM; **3c**: 1243 nM) and human breast adenocarcinoma (**3b**: IC_50_ = 998 nM; **3c**: 3064 nM) cell lines. Interestingly, for the human prostate cancer cell line, a different trend was observed (**3b**: IC_50_ = 2440 nM; **3c**: 1296 nM). The *N*-allyl group outperformed both the methyl and ethyl groups in terms of activity, suggesting that the alkene functionality is somehow important to the mechanism of cell growth inhibition. It is believed that the indoloquinolines inhibit cell growth by direct interactions with DNA, although the exact mechanism(s) remain uncertain [28,44,47,50,70].

Several of the tested compounds were found to display no activity against the panel of cancer cell lines, including novel compounds **10** and **21**. Another compound which was observed to be inactive was neocryptolepine precursor **13**, being inactive against all three cell lines. The isocryptolepine precursor **14** showed poor activity against all cancer cell lines and further highlights the necessity of the *N*-methyl group for cell growth inhibition.

The importance of incorporating an *N*-methyl is further demonstrated in compounds **4a** and its corresponding *N*-methylated product **8a**, showing an increase in activity against the HCT116 and MDA-MB-231 cell lines, favoring the inclusion of an *N*-methyl group. In the PC-3 cell lines, the pyridophenanthridines **4** showed a decrease in activity with the addition of an *N*-methyl substituent to give the corresponding compound **8**. However, the assay revealed the methoxy pyridophenanthridine **4b** to contain potent anticarcinogenic properties (IC_50_ = 24 nM) against the PC-3 cell line. Compound **4b** showed a 10-fold and 35-fold increase in activity compared to the positive controls Puromycin (IC_50_ = 270 nM) and Doxorubicin (IC_50_ = 830 nM), respectively. The positioning of the methoxy substituent at C-6 of the pyridophenanthridine scaffold appears to be key to the observed increase in activity, as the naked pyridophenanthridine **4a** showed only modest activity against the PC-3 cell line (IC_50_ = 1630 nM). A previous study by Lu and coworkers demonstrated the potential of the strategic installation of appropriate ring-substituents to obtain increased antiproliferative activity in various indolo[3,2-*b*]quinolines [71]. Similar to the observations made in this work, Lu et al. noted the potency of C-9 ester substituted indoloquinolines in their screening of several cancer cell lines [71], despite the parent neocryptolepine (**2**) displaying only minor inhibition of cell growth. The *N*-methylated pyridophenanthridine **8a** evaluated in this work was further shown to be more potent against the MDA-MB-231 (IC_50_ = 360 nM) cell line than Doxorubicin (IC_50_ = 590 nM). Being novel compounds, the mode of action of the pyridophenanthridines against proliferative cancer is naturally unknown. Thus, proceeding studies have the potential to unveil a new mode of action. The discovery of new modes of action is regarded as highly important in the field of drug discovery [72], further illustrating the potential for the novel pyridophenanthridine scaffold as a lead for subsequent development into a new anticancer therapy.

### 2.4. Antimicrobial and Biofilm Iinhibition Assay

The prepared samples were tested for in vitro antimicrobial activity against *E. faecalis*, *E. coli*, *P. aeruginosa*, *S. aureus*, *Streptococcus agalactiae* and *S. epidermis* using gentamycin as a reference compound. The compounds were tested at 100, 75, 50, 25, 12.5, 10, 6.3, 3.1 and 1.6 µM and the obtained minimal inhibitory concentrations (MIC) and minimal bacterial inhibition concentrations (MBIC) can be seen in Table 3. Several of the screened compounds contained no antibacterial properties against the tested panel of bacteria, including tetracycles **8a**, **10**, **13**-**14**, **16**, **18**, and **20**-**21**, while compounds **3b**, **3c**, and **21** were not tested. 

Neocryptolepine (**2**) showed only modest activity against *Streptococcus agalactiae* (MIC = 100 µM), while its precursor **13** was inactive against all bacterial strains. It has been shown previously that neocryptolepine (**2**) only possesses bacteriostatic properties against Gram-positive bacteria and displays no activity whatsoever against Gram-negative bacteria [40,72,73,74], which fits well with our observations. With the exception of *P. aeruginosa*, isocryptolepine (**3a**) contained modest activity against all the tested strains and excellent inhibition of biofilm growth.

The novel pyridophenanthridines **4a** and **8a** were both effective against the Gram-positive bacteria *E. faecalis* (**4a**: MIC = 100 µM; **8a**: MIC = 75 µM) and *S. aureus* (**4a**: MIC = 100 µM; **8a**: MIC = 75 µM) but were inactive against the rest. These results are comparable to previous observations for the indolo[2,3-*b*]quinolines (i.e., neocryptolepines), showing that the presence of an *N*-methyl substituent is essential for antimicrobial inhibition [73]. Methoxy substituted pyridophenanthridine **4b** was proven to be the most successful in the evaluated series, being moderately effective against *E. coli* (MIC = 50 µM) and *S. aureus* (MIC = 75 µM). Interestingly, addition of the *N*-methyl functionality to produce pyridophenanthridine **8b**, resulted in a complete loss of activity. Representing unknown scaffolds, the mode of action of the pyridophenanthridines are naturally not known; however, these data indicate that the methoxy substituted **4b** and **8b** could differ from their non-functionalized counterparts **4a** and **8a**.

Novel pyridocarbazoles **9** showed excellent biofilm formation inhibition (**9a**: MBIC = 100 µM; **9b**: MIC = 100 µM) and variant **9a** was also active against *Streptococcus agalactiae* (MBIC = 100 µM). The incorporation of a fluorine into a molecule is usually associated with a significant increase in biological activity [75], which is not the case for compound **9**, having the non-fluorinated **9a** performing better overall. In general, pyridocarbazoles have been primarily studied for their antiproliferative qualities in the past, with natural products such as the ellipticines containing potent anticancer properties [76]. The ellipticines are currently employed clinically as antiproliferative agents, though little is known about the inherent antimicrobial potential of such motifs. Although the antimicrobial activities observed for compound **9** were not particularly significant, this structural motif should be explored in greater detail in future research to uncover its full potential as a dual antimicrobial and antiproliferative agent.

## 3. Materials and Methods 

### 3.1. Chemistry

#### 3.1.1. General

Nuclear magnetic resonance (NMR) spectra were recorded on a Bruker AscendTM 400 series (Billerica, MA, USA), operating at 400.13 MHz for ^1^H, 376.49 MHz for ^19^F and 100.61 MHz for ^13^C, respectively. Chemical shifts (δ) are expressed in ppm relative to residual chloroform-d (^1^H, 7.26 ppm; ^13^C, 77.16 ppm), DMSO-*d_6_* (^1^H, 2.50 ppm; ^13^C, 39.52 ppm), methanol-*d_4_* (^1^H, 3.31 ppm; ^13^C, 49.00 ppm), acetone-*d_6_* (^1^H, 2.09 ppm; 13C, 30.60 ppm), dichloromethane-*d_2_* (^1^H, 5.32 ppm; ^13^C, 53.84 ppm) or α,α,α-trifluorotoluene (^19^F, −62.61 ppm) [77] as an external reference. The assignment of signals in various NMR spectra were often assisted by conducting correlation spectroscopy (COSY), heteronuclear single-quantum correlation spectroscopy (HSQC), heteronuclear multiple bond correlation spectroscopy (HMBC) and nuclear Overhauser effect spectroscopy (NOESY). 

Reactions were monitored by thin-layer chromatography (TLC) carried out on 0.25 mm silica gel F254 coated aluminum sheets using UV light as a visualizing agent. Silica gel 60 (particle size 40–63 μm) was used for flash chromatography. 

In addition to TLC, low resolution mass spectrometry (LRMS) was routinely used to monitor and identify the various components of reaction mixtures. The LRMS spectra were obtained using an Advion expressions CMS mass spectrometer operating at 3.5 kV in electrospray ionization (ESI) mode.

Infrared spectroscopy (IR) was performed on a Agilent Technologies Cary 360 FTIR spectrophotometer (Santa Clara, CA, USA). Solids were dissolved in CHCl_3_ or CH_2_Cl_2_ and adsorbed on a NaCl plate, or by placing the sample directly onto the crystal of an attenuated total reflectance (ATR) module. Melting points were measured using a Stuart Scientific SMP3 melting point apparatus and are uncorrected. High resolution mass spectrometry (HRMS) were conducted externally at the University of Bergen (UiB) or the University of Tromsø, using ESI mode. The microwave-assisted experiments were performed in a CEM Focused Microwave^TM^ Synthesis System (Charlotte, NC, USA), model type Discover, operating at 0–300 W, a pressure of 0–290 psi, at a temperature of 118 °C, using reactor vial volumes of either 10 or 35 mL. Commercially available chemicals were used as delivered from the supplier unless otherwise noted. 

Detailed experimental procedures and full characterizations for compounds **3a**, **4**, **5b**, **7**, **15**, **17**, and **19** are available through our previous works [52,62].

#### 3.1.2. 4-Fluoro-2-(4,4,5,5-tetramethyl-1,3,2-dioxaborolan-2-yl)aniline (**6b**)

To a mixture of 2-bromo-4-fluoroaniline (1000.0 mg, 5.26 mmol), anhydrous Et_3_N (2.93 m, 21.04 mmol), PdCl_2_(PPh_3_)_2_ (369.2 mg, 0.53 mmol, 10 mol%) in 20 mL anhydrous dioxane, was added 4,4,5,5-tetramethyl-1,3,2-dioxaborolane (2.30 mL, 15.79 mmol) dropwise. The resulting mixture was refluxed for 22 h and then allowed to cool to rt before being quenched by addition of suitable amounts of sat. aq. NH_4_Cl. The crude was subsequently extracted using CH_2_Cl_2_ (3 × 20 mL) and the combined organic phases were washed with water (1 × 20 mL), brine (1 × 20 mL), dried (MgSO_4_), filtered and concentrated in vacuo. The concentrate was then evaporated onto celite and purification by silica gel column chromatography (pet. ether/EtOAc, 9:1 *v*/*v*) and concentration of the relevant fractions [*R*_f_ = 0.33 (pet. ether/EtOAc, 9:1 *v*/*v*)] gave the target compound **6b** as a red solid (975.7 mg, 78%), mp 49–50 °C (lit. [78] 50–52 °C); IR (ATR): ν_max_ 3481, 3388, 2978, 2931, 1621, 1431, 1137, 854 cm^−1^; ^1^H NMR (400 MHz, CDCl_3_): δ 7.28 (dd, *J* = 9.1 Hz, 3.1 Hz, 1H), 6.92 (ddd, *J* = 8.6 Hz, 8.3 Hz, 3.1 Hz, 1H), 6.53 (dd, *J* = 8.8 Hz, 4.3 Hz, 1H), 4.55 (bs, 2H), 1.34 (s, 12H); ^13^C NMR (100 MHz, CDCl_3_): δ 155.3 (d, *J*_CF_ = 235.0 Hz), 149.9, 121.6 (d, *J*_CF_ = 20.3 Hz), 119.8 (d, *J*_CF_ = 23.0 Hz), 116.1 (d, *J*_CF_ = 6.9 Hz), 83.9, 25.0 (one carbon was obscured or overlapping); ^19^F NMR (376 MHz, CDCl_3_): δ −129.0. The spectroscopic data are in accordance with previously reported data [78].

#### 3.1.3. 4-Fluoro-2-(quinolin-5-yl)aniline (**7c**)

To a solution of 5-bromoquinoline (**5a**) (512.3 mg, 2.46 mmol) in 25 mL 1,2-dimethoxyethane (DME) under an argon atmosphere was added 4-fluoro-2-(4,4,5,5-tetramethyl-1,3,2-dioxaborolan-2-yl)aniline (**6b**) (875.7 mg, 3.69 mmol), an aqueous solution of Cs_2_CO_3_ (2805.3 mg, 8.61 mmol in 5 mL H_2_O) and Pd(PPh_3_)_4_ (142.1 mg, 0.12 mmol). The resulting mixture was stirred at 80 °C for 17 h before being allowed to cool to rt. The volatiles were then removed under reduced pressure and the concentrate was evaporated onto celite. Purification by silica gel column chromatography (pet. ether/EtOAc, 1:1 *v*/*v*) and concentration of the relevant fractions [*R*_f_ = 0.10 (pet. ether/EtOAc, 1:1 *v*/*v*)] gave the target compound **7c** as an orange solid (419.5 mg, 72%), mp 196–197 °C; IR (ATR): ν_max_ 3041, 2921, 2852, 1635, 1490, 1192, 900, 792 cm^−1^; ^1^H NMR (400 MHz, CD_2_Cl_2_): δ 8.90 (dd, *J* = 4.1 Hz, 1.7 Hz, 1H), 8.14–8.12 (m, 1H), 7.96 (ddd, *J* = 8.5 Hz, 1.6 Hz, 0.8 Hz, 1H), 7.79 (dd, J = 8.5 Hz, 7.1 Hz, 1H), 7.51 (dd, *J* = 7.0 Hz, 1.1 Hz, 1H), 7.36 (dd, *J* = 8.5 Hz, 4.2 Hz, 1H), 7.00 (td, *J* = 8.6 Hz, 3.0 Hz, 1H), 6.88 (dd, *J* = 9.0 Hz, 3.0 Hz, 1H), 6.79 (dd, *J* = 8.8 Hz, 4.8 Hz, 1H), 3.42 (bs, 2H) (Figure S3.1); ^13^C NMR (100 MHz, CD_2_Cl_2_): δ 156.4 (d, *J*_CF_ = 235.8 Hz), 151.0, 149.1, 141.4, 136.8, 134.5, 130.1, 129.6, 128.3, 127.0, 125.7 (d, *J*_CF_ = 7.2 Hz), 121.8, 117.7 (d, *J*_CF_ = 22.1 Hz), 116.6 (d, *J*_CF_ = 8.0 Hz), 115.9 (d, *J*_CF_ = 22.1 Hz) (Figure S3.2); ^19^F NMR (376 MHz, CD_2_Cl_2_): δ -128.0 (Figure S3.3); HRMS (ESI): calcd. for C_15_H_11_FN_2_ [M + H^+^] 239.0979, found 239.0988.

#### 3.1.4. Intramolecular Cyclization to Form Tetracycles **9**, **13**, **14**, **18** and **20**

##### General Procedures 

Method 1—palladium-catalyzed intramolecular C-H activation/C-N bond formation: The appropriate biaryl (1 equiv.) in a suitable amount of glacial acetic acid was added to a premixed solution of PdCl_2_(dppf) (10 mol%), 1,3-bis(2,4,6-trimethylphenyl)imidazolium (IMes) (5 mol%), H_2_O_2_ (35 wt%, 29 mol%) and a suitable amount of glacial acetic acid. The reaction mixture was then placed in a sealed reactor tube and immersed into the cavity of a microwave oven and heated at 118 °C until completion as indicated by TLC analysis. The reaction mixture was then transferred to a round-bottom flask with the aid of EtOAc/CHCl_3_ and the volatiles were removed under reduced pressure. The reaction mixture was finally evaporated onto celite and purified by column chromatography with the eluents as indicated in order to give the target compounds. 

Method 2—diazotization-azidation-nitrene insertion: The appropriate biaryl (1 equiv.) was dissolved in a suitable amount of aq. HCl (37%) and the mixture was cooled to 0 °C using an ice bath. Next, to ice-cooled aq. NaNO_2_ (0.4 M) was added the solution dropwise and the resulting mixture was stirred at 0 °C for 1.5 h. An ice-cooled aq. solution of NaN_3_/NaOAc (2.1 equiv./14 equiv. in an appropriate amount of H_2_O) was added dropwise and the mixture stirred for 1 h while keeping the temperature at 0 °C. The reaction mixture was quenched by addition of appropriate amounts of sat. aq. K_2_CO_3_ and subsequently extracted with CH_2_Cl_2_ (3 × 20 mL). The combined organic phases were washed with water (1 × 20 mL), brine (1 × 20 mL), dried (MgSO_4_), filtered and concentrated in vacuo. The obtained residue was dissolved in a suitable amount of 1,2-dichlorobenzene and flushed with argon. The resulting mixture was stirred at 180 °C for 3 h under an argon atmosphere before being cooled to rt. The solvent was removed under reduced pressure and the concentrate was evaporated onto celite and purified by column chromatography using the eluents as indicated in order to give the target compounds. 

##### 7*H*-Pyrido[2,3-c]carbazole (**9a**)

Method 2: Following the general procedure, the title compound was prepared from 2-(quinolin-5-yl)aniline (**7a**) (100.0 mg, 0.45 mmol), HCl (37%, 3 mL), NaNO_2_ (82.9 mg, 1.20 mmol in 3 mL H_2_O), NaN_3_ (61.4 mg, 0.94 mmol) and NaOAc (516.8 mg, 6.30 mmol in 5 mL H_2_O). After formation of the azide was confirmed by IR, the cyclization was carried out using 3 mL of 1,2-dichlorobenzene. The crude was purified by silica gel column chromatography (CH_2_Cl_2_/EtOAc, 95:5 → 9:1 *v*/*v*) and concentration of the relevant fractions [*R*_f_ = 0.22 (CH_2_Cl_2_/EtOAc, 95:5 *v*/*v*)] gave the target compound **9a** as a light brown solid (78.8 mg, 80%), mp 204–205 °C; IR (ATR): ν_max_ 3045, 2919, 2842, 1523, 1274, 956, 804, 728 cm^−1^; ^1^H NMR (400 MHz, DMSO-*d_6_*): δ 11.92 (bs, 1H), 9.17 (dd, *J* = 8.4 Hz, 1.4 Hz, 1H), 8.84 (dd, *J* = 4.1 Hz, 1.4 Hz, 1H), 8.60 (d, *J* = 8.0 Hz, 1H), 8.03–7.98 (m, 2H), 7.69–7.65 (m, 2H), 7.47–7.44 (m, 1H), 7.34.7.31 (m, 1H) (Figure S6.1, S6.3, and S6.4); ^13^C NMR (100 MHz, DMSO-*d_6_*): δ 146.4, 144.3, 139.0, 136.9, 130.8, 127.7, 124.5, 124.3, 122.9, 121.6, 121.5, 119.8, 116.8, 113.6, 111.9 (Figure S6.2, S6.5, and S6.6); HRMS (ESI): calcd. for C_15_H_10_N_2_ [M + H^+^] 219.0917, found 219.0927. 

##### 10-Fluoro-7*H*-pyrido[2,3-c]carbazole (**9b**)

Method 2: Following the general procedure, the title compound was prepared from 4-fluoro-2-(quinolin-5-yl)aniline (**7c**) (419.5 mg, 1.76 mmol), HCl (37%, 8 mL), NaNO_2_ (137.9 mg, 2.00 mmol), NaN_3_ (240.5 mg, 3.70 mmol) and NaOAc (2021.2 mg, 24.64 mmol in 15 mL H_2_O). The crude was essentially pure by ^1^H NMR and 100.0 mg of the azide was dissolved in 2 mL 1,2-dichlorobenzene and reacted without any further purification. The cyclization yielded a reaction crude which was also pure by NMR and the target compound **9b** was obtained as a dark green solid (87.3 mg, 97%), mp 256–257 °C; IR (ATR): ν_max_ 3137, 2974, 2746, 1460, 1149, 789 cm^−1^; ^1^H NMR (400 MHz, CD_3_OD): δ 9.07 (ddd, *J* = 8.4 Hz, 1.6 Hz, 0.8 Hz, 1H), 8.74 (dd, *J* = 4.4 Hz, 1.6 Hz, 1H), 8.18–8.15 (m, 1H), 7.98 (dd, *J* = 9.1 Hz, 0.7 Hz, 1H), 7.91 (d, *J* = 9.1 Hz, 1H), 7.67 (dd, *J* = 8.4 HZ, 4.4 HZ, 1H), 7.58 (ddd, *J* = 8.8 Hz, 4.5 Hz, 0.5 Hz, 1H), 7.25-7.20 (m, 1H) (Figure S7.1, S7.4, and S7.5); ^13^C NMR (100 MHz, CD_3_OD): δ 159.2 (d, JCF = 234.5 Hz), 146.9, 145.1, 139.8, 137.3, 132.9, 127.9, 126.4, 124.7 (d, *J*_CF_ = 9.5 Hz), 122.7, 118.3, 115.1 (d, *J*_CF_ = 5.3 Hz), 113.7 (d, *J*_CF_ = 24.0 Hz), 113.5 (d, *J*_CF_ = 7.2 Hz), 107.6 (d, *J*_CF_ = 24.8 Hz) (Figure S7.2, S7.6, and S7.7); ^19^F NMR (376 MHz, CD_3_OD): δ -123.6 (Figure S7.3); HRMS (ESI): calcd. for C_15_H_9_FN_2_ [M + H^+^] 237.0823, found 237.0830. 

##### 6*H*-Indolo[2,3-b]quinoline (**13**) and 11H-indolo[3,2-c]quinoline (**14**)

Method 2: Following the general procedure, the title compounds were prepared starting from 2-(quinolin-3-yl)aniline (**12**) (100.0 mg, 0.45 mmol), HCl (37%, 3 mL), NaNO_2_ (82.8 mg, 1.20 mmol in 3 mL H_2_O), NaN_3_ (61.4 mg, 0.94 mmol) and NaOAc (516.8 mg, 6.30 mmol in 5 mL H_2_O). After formation of the azide was confirmed by IR, the cyclization was carried out using 3 mL 1,2-dichlorobenzene. The crude was purified by silica gel column chromatography (CH_2_Cl_2_/EtOAc, 8:2 → 0:1 *v*/*v*) and concentration of the relevant fractions [*R*_f_ = 0.56 (CH_2_Cl_2_/EtOAc, 2:8 *v*/*v*)] gave compound **13** as off-white crystals (4.2 mg, 4%) along with compound **14** as an off-white solid (86.4 mg, 88%). 

##### Characterization of Compound **13**

mp 341–342 °C (lit. [79] 342–346 °C); IR (ATR): ν_max_ 3139, 2923, 2849, 1402, 725 cm^−1^; ^1^H NMR (400 MHz, DMSO-*d_6_*): δ 11.72 (bs, 1H), 9.06 (s, 1H), 8.26 (d, *J* = 7.7 Hz, 1H), 8.11 (dd, *J* = 8.1 Hz, 1.3 Hz, 1H), 7.99–7.97 (m, 1H), 7.75–7.70 (m, 1H), 7.55–7.46 (m, 3H), 7.29–7.25 (m, 1H); ^13^C NMR (100 MHz, DMSO-*d_6_*): δ 152.7, 146.1, 141.4, 128.7, 128.6, 128.2, 127.7, 126.8, 123.6, 122.8, 121.8, 120.3, 119.7, 118.0, 110.9. The spectroscopic data are in accordance with previously reported data [79].

##### Characterization of Compound **14**

mp 333-335 °C (lit. [62] 340–341 °C); IR (NaCl): ν_max_ 3060, 2958, 2854, 1682, 1582, 1515, 1493 cm^−1^; ^1^H NMR (400 MHz, DMSO-*d_6_*): δ 12.74 (bs, 1H), 9.60 (s, 1H), 8.52 (dd, *J* = 7.8 Hz, 1.1 Hz, 1H), 8.33–8.31 (m, 1H), 8.14 (dd, *J* = 8.4 Hz, 1.1 Hz, 1H), 7.77–7.68 (m, 3H), 7.52–7.48 (m, 1H), 7.37–7.33 (m, 1H); ^13^C NMR (100 MHz, DMSO-*d_6_*): δ 145.3, 144.7, 139.8, 138.8, 129.4, 128.1, 125.7, 125.6, 122.1, 121.9, 120.6, 120.1, 117.1, 114.3, 111.9. The spectroscopic data are in accordance with previously reported data [62].

##### 11*H*-Pyrido[2,3-a]carbazole (**18**)

Method 2: Following the general procedure, the title compound was prepared from 2-(quinolin-7-yl)aniline (**17**) (100.0 mg, 0.45 mmol), HCl (37%, 3 mL), NaNO_2_ (82.8 mg, 1.20 mmol in 3 mL H_2_O), NaN_3_ (61.4 mg, 0.94 mmol) and NaOAc (516.8 mg, 6.30 mmol in 3 mL H_2_O). After formation of the azide was confirmed by IR, the cyclization was carried out using 3 mL 1,2-dichlorobenzene. The crude was purified by silica gel column chromatography (CH_2_Cl_2_/EtOAc, 9:1 *v*/*v*) and concentration of the relevant fractions [*R*_f_ = 0.36 (CH_2_Cl_2_/EtOAc, 9:1 *v*/*v*)] gave the target compound **18** as off-white crystals (40.0 mg, 41%), mp 164 °C (lit. [52] 165–167 °C); IR (ATR): ν_max_ 3263, 3043, 2923, 2854, 1523, 1369, 820, 734 cm^−1^; ^1^H NMR (400 MHz, CDCl_3_): δ 10.20 (bs, 1H), 8.92 (dd, *J* = 4.4 Hz, 1.5 Hz, 1H), 8.35 (dd, *J* = 8.3 Hz, 1.5 Hz, 1H), 8.24 (d, *J* = 8.5 Hz, 1H), 8.19–8.17 (m, 1H), 7.62–7.60 (m, 2H), 7.51–7.47 (m, 2H), 7.35–7.31 (m, 1H); ^13^C NMR (100 MHz, CDCl_3_): δ 147.8, 139.2, 137.4, 136.8, 134.9, 127.3, 125.9, 123.8, 121.7, 120.8, 120.5, 120.4, 120.2, 118.8, 111.8. The spectroscopic data are in accordance with previously reported data [52].

##### 7*H*-Pyrido[3,2-c]carbazole (**20**)

Method 2: Following the general procedure, the title compound was prepared from 2-(quinolin-8-yl)aniline (**19**) (450.0 mg, 2.04 mmol), HCl (37%, 10 mL), NaNO_2_ (137.9 mg, 2.00 mmol in 5 mL H_2_O), NaN_3_ (278.5 mg, 4.28 mmol) and NaOAc (2342.8 mg, 28.56 mmol in 10 mL H_2_O). After formation of the azide was confirmed by IR, the cyclization was carried out using 5 mL 1,2-dichlorobenzene. The crude was purified by silica gel column chromatography (pet. ether/EtOAc, 1:1 *v*/*v*) and concentration of the relevant fractions [*R*_f_ = 0.85 (pet. ether/EtOAc, 1:1 *v*/*v*)] gave the target compound **20** as a dark red oil (195.9 mg, 44%). IR (ATR): ν_max_ 3207, 2976, 2919, 2850, 2740, 2605, 2499 cm^−1^; ^1^H NMR (400 MHz, DMSO-*d_6_*): δ 11.92 (bs, 1H), 9.02 (dd, *J* = 4.4 Hz, 1.8 Hz, 1H), 8.90–8.88 (m, 1H), 8.46 (dd, *J* = 8.1 Hz, 1.4 Hz, 1H), 7.92 (d, *J* = 8.8 Hz, 1H), 7.84 (d, *J* = 8.8 Hz, 1H), 7.66–7.64 (m, 1H), 7.49 (dd, *J* = 8.0 Hz, 4.3 Hz, 1H), 7.46–7.42 (m, 1H), 7.33-7.29 (m, 1H); ^13^C NMR (100 MHz, DMSO-*d_6_*): δ 149.8, 145.3, 139.6, 138.5, 136.5, 126.0, 124.5, 123.1, 122.9, 122.8, 119.7, 118.3, 115.3, 114.2, 111.4. The spectroscopic data are in accordance with previously reported data [52].

#### 3.1.5. Neocryptolepine (**2**)

To a solution of 6*H*-indolo[2,3-*b*]quinoline (**13**) (23.0 mg, 0.10 mmol) in 2 mL THF, iodomethane (0.66 mL, 10.0 mmol) was added and the resulting mixture refluxed for 24 h. The volatiles were then removed under reduced pressure and the concentrate was evaporated onto celite. Purification by silica gel column chromatography (CH_2_Cl_2_/MeOH, 95:5 *v*/*v*) and concentration of the relevant fractions [*R*_f_ = 0.18 (CH_2_Cl_2_/MeOH, 95:5 *v*/*v*)] gave the hydroiodide salt of neocryptolepine. To obtain the free base, the hydroiodide salt was dissolved in a 20 mL 1:1 mixture of CH_2_Cl_2_ and NH_3_(aq) (20%) and stirred at rt for 30 min. The organic layer was then separated and the aqueous layers were extracted with CH_2_Cl_2_ (3 × 10 mL) and the combined organic layers were washed with water (1 × 10 mL), brine (1 × 10 mL), dried (MgSO_4_), filtered and concentrated in vacuo to give neocryptolepine (**2**) as a dark yellow solid (19.5 mg, 84%), mp 85–86 °C (lit. [67] 104–105 °C); IR (ATR): ν_max_ 3051, 2961, 2923, 2852, 1494, 1012, 741 cm^−1^; ^1^H NMR (400 MHz, CD_3_OD): δ 8.67 (s, 1H), 8.05-8.02 (m, 2H), 7.90 (d, *J* = 8.6 Hz, 1H), 7.83–7.78 (m, 1H), 7.59–7.57 (m, 1H), 7.50–7.45 (m, 2H), 7.19 (td, *J* = 7.5 Hz, 1.0 Hz, 1H), 4.23 (s, 3H); ^13^C NMR (100 MHz, CD_3_OD): δ 156.9, 155.4, 138.1, 132.0, 131.2, 130.4, 130.1, 128.4, 124.9, 123.6, 122.4, 122.2, 121.2, 117.7, 115.7, 33.7. The spectroscopic data are in accordance with previously reported data [67].

#### 3.1.6. 5-Ethyl-5*H*-indolo[3,2-c]quinoline (**3b**)

To a solution of 11*H*-indolo[3,2-*c*]quinoline (**14**) (15.0 mg, 0.068 mmol) in 3 mL toluene, ethyl iodide (1.1 mL, 13.68 mmol) was added and the resulting mixture was refluxed for 3 h. The volatiles were then removed under reduced pressure and the concentrate was evaporated onto celite. Purification by silica gel column chromatography (CHCl_3_/MeOH, 9:1 *v*/*v*) and concentration of the relevant fractions [*R*_f_ = 0.21 (CHCl_3_/MeOH, 9:1 *v*/*v*)] gave the hydroiodide salt of compound **3b**. To obtain the free base, the hydroiodide salt was dissolved in a 40 mL 1:1 mixture of CH_2_Cl_2_ and NH_3_ (aq) (20%) and stirred at rt for 5 min. The organic layer was then separated and the aqueous layers were extracted with CH_2_Cl_2_ (2 × 10 mL) and the combined organic layers were washed with brine (1 × 10 mL), dried (MgSO_4_), filtered and concentrated in vacuo to give the target compound **3b** as a yellow solid (11.0 mg, 64%), mp 198 °C. IR (NaCl): ν_max_ 3371, 3049, 2960, 2927, 2856, 1731, 1640, 1598, 1455, 1392, 1353 cm^−1^; ^1^H NMR (400 MHz, DMSO-*d_6_*): δ 9.59 (s, 1H), 8.84 (dd, *J* = 8.1 Hz, 1.3 Hz, 1H), 8.23 (d, *J* = 8.7 Hz, 1H), 8.20–8.18 (m, 1H), 7.93–7.89 (m, 1H), 7.81 (d, *J* = 8.1 Hz, 1H), 7.79–7.75 (m, 1H), 7.51–7.47 (m, 1H), 7.34–7.30 (m, 1H), 4.81 (q, *J* = 7.1 Hz, 2H), 1.56 (t, *J* = 7.1 Hz, 3H) (Figure S1.1, S1.3, and S1.4); ^13^C NMR (100 MHz, DMSO-*d_6_*): δ 151.2, 150.5, 138.6, 134.3, 130.1, 126.1, 125.8, 124.7, 124.4, 120.5, 120.2, 119.9, 117.8, 117.2, 115.9, 49.7, 15.0 (Figure S1.2, S1.5, and S1.6); HRMS (ESI): calcd. for C_17_H_14_N_2_ [M + H^+^] 247.1235, found 247.1238. 

#### 3.1.7. 5-Allyl-5*H*-indolo[3,2-c]quinoline (**3c**)

To a solution of 11*H*-indolo[3,2-*c*]quinoline (**14**) (30.0 mg, 0.14 mmol) in 5 mL toluene, allyl bromide (1.14 mL, 13.76 mmol) was added and the resulting mixture was refluxed for 22 h. The volatiles were then removed under reduced pressure and the concentrate was evaporated onto celite. Purification by silica gel column chromatography (CHCl_3_/MeOH, 95:5 → 9:1 *v*/*v*) and concentration of the relevant fractions [*R*_f_ = 0.18 (CHCl_3_/MeOH, 9:1 *v*/*v*)] gave the hydroiodide salt of compound **3c**. To obtain the free base, the hydroiodide salt was dissolved in a 20 mL 1:1 mixture of CH_2_Cl_2_ and NH_3_ (aq) (20%) and stirred at rt for 45 min. The organic layer was then separated and the aqueous layers were extracted with CH_2_Cl_2_ (3 × 10 mL) and the combined organic layers were washed with water (1 × 10 mL), brine (1 × 10 mL), dried (MgSO_4_), filtered and concentrated in vacuo to give the target compound **3c** as a yellow viscous oil (15.5 mg, 43%). IR (ATR): ν_max_ 2924, 2723, 1596, 1349, 1204, 927, 743 cm^−1^; ^1^H NMR (400 MHz, DMSO-*d_6_*): δ 9.67 (s, 1H), 8.84 (dd, *J* = 8.0 Hz, 1.3 Hz, 1H), 8.22–8.20 (m, 1H), 8.15 (d, *J* = 8.7 Hz, 1H), 7.92–7.88 (m, 1H), 7.84–7.77 (m, 2H), 7.55–7.51 (m, 1H), 7.37–7.34 (m, 1H), 6.26–6.17 (m, 1H), 5.46–5.44 (m, 2H), 5.31–5.28 (m, 1H), 5.15–5.11 (m, 1H) (Figure S2.1, S2.3, and S2.4); ^13^C NMR (100 MHz, DMSO-*d_6_*): δ 150.0, 149.9, 139.6, 134.9, 132.8, 130.3, 126.5, 126.1, 124.4, 124.3, 121.0, 120.1, 119.7, 118.5, 118.2, 116.8, 115.7, 56.5 (Figure S2.2 and S2.5); HRMS (ESI): calcd. for C_18_H_14_N_2_ [M + H^+^] 259.1230, found 259.1232.

#### 3.1.8. 4-Methyl-4*H*-pyrido[4,3,2-gh]phenanthridine (**8a**)

To a solution of 7*H*-pyrido[4,3,2-*gh*]phenanthridine (**4a**) (70.0 mg, 0.32 mmol) in 2 mL acetonitrile, iodomethane (2.0 mL, 32.0 mmol) was added and the resulting mixture was refluxed for 2 h. The volatiles were then removed under reduced pressure and the concentrate was evaporated onto celite. Purification by silica gel column chromatography (CHCl_3_/MeOH, 95:5 + 0.3% NH_3_ (aq) *v*/*v*) and concentration of the relevant fractions [*R*_f_ = 0.33 (CHCl_3_/MeOH, 95:5 + 0.3% NH_3_(aq) *v*/*v*)] gave the hydroiodide salt of compound **8a**. To obtain the free base, the hydroiodide salt was dissolved in a 20 mL 1:1 mixture of CH_2_Cl_2_ and NH_3_ (aq) (20%) and stirred at rt for 20 min. The organic layer was separated and the aqueous layers were extracted with Et_2_O (2 × 20 mL) and the combined organic layers were washed with water (1 × 10 mL), brine (1 × 10 mL), dried (MgSO_4_), filtered and concentrated in vacuo to give the target compound **8a** as dark yellow crystals (52.8 mg, 71%), mp 182–183 °C; IR (ATR): ν_max_ 3485, 3051, 2922, 2851, 2574, 1601, 1327, 820, 748 cm^−1^; ^1^H NMR (400 MHz, DMSO-*d_6_*): δ 8.29 (dd, *J* = 8.1 Hz, 1.1 Hz, 1H), 7.97 (d, *J* = 7.9 Hz, 1H), 7.71 (t, *J* = 8.1 Hz, 1H), 7.56 (dd, *J* = 8.2 Hz, 1.2 Hz, 1H), 7.51–7.47 (m, 2H), 7.30–7.26 (m, 1H), 7.05 (d, *J* = 8.1 Hz, 1H), 6.18 (d, *J* = 7.6 Hz, 1H), 3.45 (s, 3H) (Figure S4.1, S4.3, and S4.4); ^13^C NMR (100 MHz, DMSO-*d_6_*): "δ" 152.9, 145.9, 141.0, 140.9, 133.8, 131.6, 129.2, 127.0, 123.2, 122.9, 121.3, 119.6, 112.2, 108.8, 106.2, 39.6 (Figure S4.2, S4.5, and S4.6); HRMS (ESI): calcd. for C_16_H_12_N_2_ [M + H^+^] 233.1073, found 233.1073.

#### 3.1.9. 6-Methoxy-4-methyl-4*H*-pyrido[4,3,2-gh]phenanthridine (**8b**)

To a solution of 6-methoxy-7*H*-pyrido[4,3,2-*gh*]phenanthridine (**4b**) (90.0 mg, 0.36 mmol) in 10 mL acetonitrile, iodomethane (2.25 mL, 36.3 mmol) was added and the resulting mixture refluxed for 2 h. The volatiles were then removed under reduced pressure and the concentrate was evaporated onto celite. Purification by silica gel column chromatography (EtOH + 0.1 → 5% NH_3_ (aq) *v*/*v*) and concentration of the relevant fractions [*R*_f_ = 0.23 (EtOH)] gave the hydroiodide salt of compound **8b**. To obtain the free base, the hydroiodide salt was dissolved in a 20 mL 1:1 mixture of CH_2_Cl_2_ and NH_3_ (aq) (20%) and stirred at rt for 20 min. The organic layer was separated and the aqueous layers were extracted with CH_2_Cl_2_ (4 × 20 mL) and the combined organic layers were washed with water (1 × 20 mL), brine (1 × 20 mL), dried (MgSO_4_), filtered and concentrated in vacuo to give the target compound **8b** as a dark yellow gel (55.1 mg, 58%). IR (ATR): ν_max_ 2918, 2850, 1600, 1255, 1059, 745 cm^−1^; ^1^H NMR (400 MHz, CD_2_Cl_2_): δ 8.19 (dd, *J* = 8.2 Hz, 1.3 Hz, 1H), 7.79 (dd, *J* = 8.2 Hz, 1.0 Hz, 1H), 7.72 (d, *J* = 7.9 Hz, 1H), 7.57–7.51 (m, 2H), 7.34–7.30 (m, 1H), 6.74–6.72 (m, 2H), 3.85 (s, 3H), 3.34 (s, 3H) (Figure S5.1, S5.3 and S5.4); ^13^C NMR (100 MHz, CD_2_Cl_2_): δ 148.1, 146.3, 140.8, 139.9, 134.8, 130.9, 129.4, 128.6, 124.1, 122.7, 122.5, 119.9, 110.6, 107.7, 57.1, 40.4 (one carbon was obscured or overlapping) (Figure S5.2, S5.5 and S5.6); HRMS (ESI): calcd. for C_17_H_14_N_2_O 263.1179, found 263.1188. 

#### 3.1.10. 4-Methyl-7*H*-pyrido[2,3-c]carbazolium Iodide (**10**)

To a solution of 7*H*-pyrido[2,3-*c*]carbazole (**9a**) (40.7 mg, 0.19 mmol) in 5 mL acetonitrile, iodomethane (1.20 mL, 19.6 mmol) was added and the resulting mixture refluxed for 20 h. The volatiles were then removed under reduced pressure and the concentrate was evaporated onto celite. Purification by alumina gel column chromatography (CHCl_3_/MeOH, 9:1 *v*/*v* + 1% NH_3_ (aq)) and concentration of the relevant fractions [*R*_f_ = 0.12 (CHCl_3_/MeOH, 9:1 *v*/*v* + 2% NH_3_ (aq))] gave the target compound **10** as a bright yellow solid (20.9 mg, 47%), mp 284–286; IR (ATR): ν_max_ 3353, 3043, 3006, 2960, 2921, 2853, 1556, 1370, 1326, 741 cm^−1^; ^1^H NMR (400 MHz, DMSO-*d_6_*): δ 12.84 (bs, 1H), 9.99 (d, *J* = 8.4 Hz, 1H), 9.39 (d, *J* = 5.6 Hz, 1H), 8.76 (d, *J* = 8.1 Hz, 1H), 8.50 (d, *J* = 9.3 Hz, 1H), 8.43 (d, *J* = 9.4 Hz, 1H), 8.22 (dd, *J* = 8.5 Hz, 5.7 Hz, 1H), 7.82 (d, *J* = 8.2 Hz, 1H), 7.64-7.60 (m, 1H), 7.48–7.44 (m, 1H), 4.74 (s, 3H) (Figure S8.1, S8.3, and S8.4); ^13^C NMR (100 MHz, DMSO-*d_6_*): δ 145.0, 140.8, 139.8, 137.5, 134.5, 126.6, 125.8, 122.4, 122.1, 121.9, 121.6, 121.1, 116.0, 114.3, 112.8, 46.3 (Figure S8.2, S8.5 and S8.6); HRMS (ESI): calcd. for C_16_H_13_N_2_I [M – I^-^] 233.1073, found 233.1073.

#### 3.1.11. 4-Methyl-11*H*-pyrido[3,2-a]carbazolium Iodide (**21**)

To a solution of 11*H*-pyrido[3,2-*a*]carbazole (**15**) (32.1 mg, 0.15 mmol) in 5 mL acetonitrile, iodomethane (0.92 mL, 14.72 mmol) was added and the resulting mixture stirred at reflux for 20 h. The volatiles were then removed under reduced pressure and the obtained yellow crystals were thoroughly washed with n-hexanes and dried in vacuo to give the target compound **21** as a dark orange crystalline solid (47.6 mg, quant.), mp 279–280 °C; IR (ATR): ν_max_ 3416, 3165, 3077, 2997, 2905, 1599, 1452, 1371, 740 cm^−1^; ^1^H NMR (400 MHz, CD_3_OD): δ 9.64 (d, *J* = 8.6 Hz, 1H), 9.28 (d, *J* = 5.7 Hz, 1H), 8.99 (d, *J* = 9.1 Hz, 1H), 8.35–8.33 (m, 1H), 8.15–8.10 (m, 2H), 7.79–7.77 (m, 1H), 7.65–7.61 (m, 1H), 7.46–7.42 (m, 1H), 4.76 (s, 3H) (Figure S9.1, S9.3, and S9.4); ^13^C NMR (100 MHz, CD_3_OD): δ 148.2, 141.7, 141.6, 139.1, 136.1, 131.1, 128.4, 123.6, 122.4, 122.3, 121.6, 121.1, 119.9, 113.1, 109.0, 46.9 (Figure S9.2, S9.5 and S9.6); HRMS (ESI): calcd. for C_16_H_13_N_2_I [M – I^-^] 233.1073, found 233.1075.

### 3.2. Biological Testing Assay

#### 3.2.1. General

All compounds for antimicrobial testing were diluted to a final assay concentration of 40 µL, 0.4% DMSO, and tested in full dose-response using three concentrations per log dose (16 points with a concentration range of 0.33 nM–40 µM, for reference compounds: 21 points with a concentration of 0.01 nM–40 µM).

All compounds for antiplasmodial testing were diluted to a final assay concentration of 40 µL, 0.4% DMSO, and tested in full dose-response using three concentrations per log dose (16 points with a concentration range of 0.4 nM–40 µM, for reference compounds: 16 points with a concentration rage of 0.4 nM–40 µM for chloroquine and puromycin: 0.001 nM–0.1 µM for artemisinin). Compounds tested in the antiproliferative assays were tested in 11 dilution points (0.02 µM–40 µM or 0.04 µM–80 µM).

##### Antiplasmodial Imaging Assay 

Antiplasmodial activity was determined as previously described by Duffy and Avery [80]. Briefly, compounds were incubated with 2% parasitemia in 0.3% hematocrit, in an assay volume of 50 µL for 72 h at 37 °C and 5% CO_2_ in CellCarrier Ultra 384-well PDL-imaging plates. After incubation, plates were stained with 4’,6-diamidino-2-phenylindole (DAPI) in a permeabilization buffer for 5 h at rt in the dark. Plates were imaged on the Opera confocal microplate image reader (PerkinElmer). Parasite inhibition was calculated using the minimum (0.4% DMSO) and maximum (5 µM puromycin) controls, and IC_50_ values determined using GraphPad Prism software. 

##### Cytotoxicity Assay

The cytotoxicity of compounds was determined using a resazurin-based viability assay in HEK293 (ATCC^®^, CRL-1573), as described by Fletcher and Avery [81]. Compounds were added to TC-treated 384-well plates (Greiner, Kremsmünster, AT) containing 2500 HEK293 cells per well, total assay volume of 50 µL and incubated for 72 h at 37 °C, 5% CO_2_. After incubation, media was removed, replaced with 44 µM resazurin and plates incubated 6 h under the same experimental conditions. Fluorescence was measured using an EnSight plate reader (PerkinElmer, Waltham, MA, USA). Cell viability was calculated using positive (45 µM puromycin) and negative (0.4% DMSO) controls, and the IC_50_ values determined using GraphPad Prism software. 

##### Antiproliferative Assay

Antiproliferative activity was assessed in HCT116 (ATCC^®^ CCL-247; 1000 cells/well), MDA-MB-231 (ATCC^®^ HTB-26; 2000 cells/well) and PC-3 (ATCC^®^ CRL-1435; 1000 cells/well) cells. HCT116 cells were maintained in McCoy’s 5A media (Life Technologies, CA, USA), MDA-MB-231 cells were cultured in DMEM media (Life Technologies) with 10 mM HEPES (Life Technologies), whilst PC-3 cells were maintained in RPMI media (Life Technologies). All media were supplemented with 10% heat-inactivated fetal bovine serum (Australian source; Corning, CA, USA) and all cells were incubated at 37 °C in a humidified incubator with 5% CO_2_. 

Cells were seeded in 384-well plates (Greiner Bio-One, NC, USA) using the respective complete media. After 24 h cell seeding, compounds were added and antiproliferative activity was determined using the resazurin assay after 72 h compound incubation. Briefly, cells were incubated with 60 µM resazurin (Cayman, MI, USA) for 6 h at 37 °C and fluorescence signals were measured using a microplate reader (EnSight, PerkinElmer, Waltham, MA, USA). Fluorescence signals were normalized to 0.4% DMSO and 50 µM puromycin and IC_50_ values were calculated from non-linear dose-response curves using GraphPad Prism 7 software (La Jolla, CA, USA). 

#### 3.2.2. Growth Inhibition Assay

To determine and quantify antimicrobial activity, a bacteria growth inhibition assay in liquid media was executed. Compounds **2**–**3**, **4**, **8**–**10**, **13**–**14**, **16**, **18**, and **20** were tested against *Staphylococcus aureus* (ATCC 25923), *Escherichia coli* (ATCC 259233), *Enterococcus faecialis* (ATCC 29122), *Pseudomonas aeruginosa* (ATCC 27853) and *Streptococcus agalactiae* (ATCC 12386); all strains from LGC Standards (Teddington, UK). *S. aureus*, *E. coli*, and *P. aeruginosa* were grown in Muller Hinton broth (275730, Becton, Franklin Lakes, NJ, USA). *E. faecalis* and *S. agalactiae* were cultured in brain hearth infusion broth (53286, Sigma, St Louise, MO, USA). Fresh bacterial colonies were transferred in the respective medium and incubated at 37 °C overnight. The bacterial cultures were diluted to a culture density representing the log phase and µL/well were pipetted into a 96-well microtiter plate (734–2097, NunclonTM, Thermo Scientific, Waltham, MA, USA). The final cell density was 1500–15.000 colony forming units/well. The compound was diluted in 2% (*v*/*v*) DMSO in ddH_2_O, providing a final assay concentration of 50% of the prepared sample, since 50 µL of sample in DMSO/water were added to 50 µL bacterial culture. After adding the samples to the plates, they were incubated overnight at 37 °C and the growth was determined by measuring the optical density at λ = 600 nm (OD600) with a 1420 Multilabel Counter VICTOR3TM (Perkin Elmer, Waltham, MA, USA). A water sample was used as a reference control, growth medium without bacteria was used as a negative control and dilution series of gentamycin (A2712, Merck, Darmstadt, DE) from 32 to 0.01 µg/mL was used as positive control and visually inspected for bacterial growth. The positive control was used as a system suitability test and the results of the antimicrobial assay were only considered valid when positive control was passed. The final concentration of DMSO in the assays was ≤2% (*v*/*v*) and was known to have no effect in the tested bacteria. The data was processed using GraphPad Prism 8. 

#### 3.2.3. Biofilm Formation Inhibition Assay 

For testing the inhibition of biofilm formation, the biofilm-producing *Staphylococcus epidermidis* (ATCC 35984) was grown in Tryptic Soy Broth (TSB, 105459, Merck, Kenilworth, NJ, USA) overnight at 37 °C. The overnight culture was diluted in fresh medium with 1% glucose (D9434, Sigma) before being transferred to a 96-well microtiter plate; 50 µL/well were incubated overnight with 50 µL of the test compound dissolved in 2% (*v*/*v*) DMSO aq. added in duplicates. The bacterial culture was removed from the plate and the plate was washed with tap water. The biofilm was fixed at 65 °C for 1 h before 70 µL 0.1% crystal violet (115940, Millipore, Burlington, MA, USA) was added to the wells for 10 min of incubation and 70 µL of 70% ethanol was then added to each well and the plate incubated on a shaker for 5–10 min. Biofilm formation inhibition was assessed by the presence of violet color and measured at 60 nm absorbance using a 1420 Multilabel Counter VICTOR3TM; 50 µL of a non-biofilm forming Staphylococcus haemolyticus (clinical isolate 8-7A, University Hospital of North Norway Tromsø, Norway) mixed in 50 µL autoclaved Milli-Q water was used as a control; 50 µL S. epidermidis mixed in 50 µL autoclaved Milli-Q water was used as the control for biofilm formation; and 50 µL TSB with 50 µL autoclaved Milli-Q water was used as a medium blank control.

## 4. Conclusions

In conclusion, a series of quinoline-based tetracyclic ring-systems were prepared and evaluated for their in vitro antiplasmodial, antiproliferative and antimicrobial activities against selected strains. Through these studies, it was determined that the ionic pyridocarbazoles **10** and **21** showed the best antiplasmodial activity against *the Plasmodium falciparum* 3D7 strains (**10**: IC_50_ = 128 nM; **21**: IC_50_ = 380 nM) of the evaluated compounds. The antiproliferative assay revealed that the novel pyridophenanthridine scaffold **4** was the most active. In particular, compound **4b** showed excellent potency against the PC-3 cell line (IC_50_ = 24 nM), significantly outperforming Puromycin (IC_50_ = 270 nM) and Doxorubicin (IC_50_ = 830 nM). The pyridophenanthridines **4** were also active against certain strains of Gram-positive and Gram-negative bacteria, with compound **4b** being moderately active against *E. coli* (MIC = 50 µM) and *Streptococcus agalactiae* (MIC = 75 µM). The antimicrobial studies further demonstrated pyridocarbazoles **9** to be highly potent against biofilm growth (**9a**: MBIC = 100 µM; **9b**: MBIC = 100 µM). Overall, this study has highlighted the potential for the novel pyridophenanthridine motif **4** and the studied pyridocarbazoles **9** to be developed into future drug candidates, with emphasis on the formulation of a dual antimicrobial and antiproliferative agent.

## Figures and Tables

**Table 1:** In vitro antiplasmodial activities of tetracyclic ring-systems **2**–**3**, **4**, **8**–**10**, **13**–**14**, **16**, **18**, **20**, and **21** against the 3D7 *P. falciparium* strain, cytotxicity against HEK293 cells and selectivity indices.

Entry	Compound	3D7 IC_50_ (nM)	Cytotoxicity IC_50_ (nM)	SI ^a^
1	Neocryptolepine (**2**)	7249 ± 6	>20,000	2.8
2	Isocryptolepine (**3a**)	1211 ± 84	2074 ± 70	1.7
3	**3b**	1318 ± 5	3078 ± 49	2.3
4	**3c**	1198 ± 32	3152 ± 40	2.6
5	**4a**	548 ± 3	2834 ± 92	5.2
6	**4b**	866 ± 2	3657 ± 2	4.2
7	**8a**	1698 ± 5	7410 ± 207	4.4
8	**8b**	1546 ± 27	5057 ± 45	3.3
9	**9a**	6825 ± 61	>80,000	11.7
10	**9b**	NT ^b^	NT ^b^	-
11	**10**	128 ± 2	NA ^c^	213.9
12	**13**	NA ^c^	NA ^c^	-
13	**14**	977 ± 11	18460 ± 183	18.9
14	**16**	NA ^c^	NA ^c^	-
15	**18**	NA ^c^	NA ^c^	-
16	**20**	2414 ± 42	NA ^c^	16.6
17	**21**	380 ± 0.5	NA ^c^	105.4
18	Chloroquine	24 ± 1	>4000	165
19	DHA	1 ± 0.07	NA ^c^	74
20	Puromycin	93 ± 2	3 ± 3	0.03

Data are presented as the mean ± standard deviation from two separate experiments. IC_50_ values were calculated using non-linear dose-response curves in GraphPad Prism. ^a^ SI = selectivity index = cytotoxicity in HEK293/activity in 3D7; ^b^ NT = not tested; ^c^ NA = not active.

**Table 2:** In vitro antiproliferative activities and cytotoxicities of tetracyclic ring-systems **2**–**3**, **4**, **8**–**10**, **13**–**14**, **16**, **18**, **20** and **21** against three cancer cell lines.

Entry	Compound	HCT116 IC_50_ (nM)	MDA-MB-231 IC_50_ (nM)	PC-3 IC_50_ (nM)
13	Neocryptolepine (**2**)	6218 ± 90	10,435 ± 375	27% at 80 µM
14	Isocryptolepine (**3a**)	667 ± 45	695 ± 130	1821 ± 7
15	**3b**	742 ± 11	998 ± 300	2440 ± 94
16	**3c**	1243 ± 80	3064 ± 467	1296 ± 51
1	**4a**	721 ± 27	594 ± 140	1630 ± 173
2	**4b**	166 ± 16 ^a^	1002 ± 297	24 ± 3 ^b^
3	**8a**	444 ± 52	360 ± 51	2571 ± 114
4	**8b**	871 ± 172	814 ± 162	4539 ± 361
5	**9a**	20,015 ± 1665	21,540 ± 2480	17,790 ± 1640
6	**9b**	NT ^c^	NT ^c^	NT ^c^
7	**10**	38% at 40 µM	24% at 40 µM	36% at 40 µM
8	**13**	NA ^d^	NA ^d^	NA ^d^
9	**14**	3573 ± 309	36% at 80 µM ^e^	30% at 80 µM ^f^
10	**16**	82% at 80 µM	80% at 80 µM	NA ^d^
11	**18**	NT ^c^	NT ^c^	NT ^c^
12	**20**	17,030 ^g^	16,415 ± 2305	47% at 40 µM
17	**21**	NA ^d^	NA ^d^	NA ^d^
18	Puromycin	85	300	270
19	Doxorubicin	150	590	830

Data are presented as the mean ± sem (standard error of the mean) from two separate experiments. IC_50_ values were calculated using non-linear dose-response curves in GraphPad Prism. ^a^ 89% metabolic activity at 40 nM; ^b^ 45% metabolic activity at 40 nM; ^c^ NT = not tested; ^d^ NA = not active; ^e^ 55% metabolic activity at 40 µM; ^f^ 50% metabolic activity at 40 µM; ^g^ 12% metabolic activity at 40 µM.

**Table 3:** In vitro antimicrobial activities of tetracyclic ring-systems **2**–**3**, **4**, and **8**–**9** against five bacterial cell lines and inhibition of biofilm formation.

Tested Strain	MIC (µM)							
	2	3a	4a ^a^	4b ^a^	8a ^a^	9a	9b	Gentamycin
*E. faecalis* (ATCC 29122)	NA ^b^	100	100	NA ^b^	75	NA ^b^	NA ^b^	8
*E. coli* (ATCC 259233)	NA ^b^	100	NA ^b^	50	NA ^b^	NA ^b^	NA ^b^	0.13
*P. aeruginosa* (ATCC 27853)	NA ^b^	NA ^b^	NA ^b^	NA ^b^	NA ^b^	NA ^b^	NA ^b^	0.25
*S. aureus* (ATCC 25923)	NA ^b^	100	100	NA ^b^	75	NA ^b^	NA ^b^	0.06
*Streptococcus agalactiae* (ATCC 12386)	100	100	NA ^b^	75	NA ^b^	100	NA ^b^	4
MBIC (µM)								
*S. epidermis* (ATCC 35984)	NA ^b^	100	NA ^b^	NA ^b^	NA ^b^	100	100	NT ^c^

^a^ Also tested at concentrations of 100, 75, 50, 25, 12.5, 10, 6.3, 3.1 and 1.6 µM. Compounds **8b**, **10**, **13**–**14**, **16**, **18**, and **20** showed no activity against any of the tested strains; ^b^ NA = not active; ^c^ NT = not tested. Compounds **3b**, **3c**, and **21** were not tested.

## Data Availability

The data presented in this study are contained within the article and Supplementary Material.

## Supplementary Materials

Figure S1.1: ^1^H NMR of 5-ethyl-5*H*-indolo[3,2-*c*]quinoline (**3b**), Figure S1.2: ^13^C NMR of 5-ethyl-5*H*-indolo[3,2-*c*]quinoline (**3b**), Figure S1.3: COSY of 5-ethyl-5*H*-indolo[3,2-*c*]quinoline (**3b**), Figure S1.4: NOESY of 5-ethyl-5*H*-indolo[3,2-*c*]quinoline (**3b**), Figure S1.5: HSQC of 5-ethyl-5*H*-indolo[3,2-*c*]quinoline (**3b**), Figure S1.6: HMBC of 5-ethyl-5*H*-indolo[3,2-*c*]quinoline (**3b**), Figure S2.1: ^1^H NMR of 5-allyl-5*H*-indolo[3,2-*c*]quinoline (**3c**), Figure S2.2: ^13^C NMR of 5-allyl-5*H*-indolo[3,2-*c*]quinoline (**3c**), Figure S2.3: COSY of 5-allyl-5*H*-indolo[3,2-*c*]quinoline (**3c**), Figure S2.4: NOESY of 5-allyl-5*H*-indolo[3,2-*c*]quinoline (**3c**), Figure S2.5: HMBC of 5-allyl-5*H*-indolo[3,2-*c*]quinoline (**3c**), Figure S3.1: ^1^H NMR of 4-fluoro-2-(quinolin-5-yl)aniline (**7c**), Figure S3.2: ^13^C NMR of 4-fluoro-2-(quinolin-5-yl)aniline (**7c**), Figure S3.3: ^19^F NMR of 4-fluoro-2-(quinolin-5-yl)aniline (**7c**), Figure S4.1: ^1^H NMR of 4-methyl-4*H*-pyrido[4,3,2-*gh*]phenanthridine (**8a**), Figure S4.2: ^13^C NMR of 4-methyl-4*H*-pyrido[4,3,2-*gh*]phenanthridine (**8a**), Figure S4.3: COSY of 4-methyl-4*H*-pyrido[4,3,2-*gh*]phenanthridine (**8a**), Figure S4.4: NOESY of 4-methyl-4*H*-pyrido[4,3,2-*gh*]phenanthridine (**8a**), Figure S4.5: HSQC of 4-methyl-4*H*-pyrido[4,3,2-*gh*]phenanthridine (**8a**), Figure S4.6: HMBC of 4-methyl-4*H*-pyrido[4,3,2-*gh*]phenanthridine (**8a**), Figure S5.1: ^1^H NMR of 6-methoxy-4-methyl-4*H*-pyrido[4,3,2-*gh*]phenanthridine (**8b**), Figure S5.2: ^13^C NMR of 6-methoxy-4-methyl-4*H*-pyrido[4,3,2-*gh*]phenanthridine (**8b**), Figure S5.3: COSY of 6-methoxy-4-methyl-4*H*-pyrido[4,3,2-*gh*]phenanthridine (**8b**), Figure S5.4: NOESY of 6-methoxy-4-methyl-4*H*-pyrido[4,3,2-*gh*]phenanthridine (**8b**), Figure S5.5: HSQC of 6-methoxy-4-methyl-4*H*-pyrido[4,3,2-*gh*]phenanthridine (**8b**), Figure S5.6: HMBC of 6-methoxy-4-methyl-4*H*-pyrido[4,3,2-*gh*]phenanthridine (**8b**), Figure S6.1: ^1^H NMR of 7*H*-pyrido[2,3-*c*]carbazole (**9a**), Figure S6.2: ^13^C NMR of 7*H*-pyrido[2,3-*c*]carbazole (**9a**), Figure S6.3: COSY of 7*H*-pyrido[2,3-*c*]carbazole (**9a**), Figure S6.4: NOESY of 7*H*-pyrido[2,3-*c*]carbazole (**9a**), Figure S6.5: HSQC of 7*H*-pyrido[2,3-*c*]carbazole (**9a**), Figure S6.6: COSY of 7*H*-pyrido[2,3-*c*]carbazole (**9a**), Figure S7.1: ^1^H NMR of 10-fluoro-7*H*-pyrido[2,3-*c*]carbazole (**9b**), Figure S7.2: ^13^C NMR of 10-fluoro-7*H*-pyrido[2,3-*c*]carbazole (**9b**), Figure S7.3: ^19^F NMR of 10-fluoro-7*H*-pyrido[2,3-*c*]carbazole (**9b**), Figure S7.4: COSY of 10-fluoro-7*H*-pyrido[2,3-*c*]carbazole (**9b**), Figure S7.5: NOESY of 10-fluoro-7*H*-pyrido[2,3-*c*]carbazole (**9b**), Figure S7.6: HS of 10-fluoro-7*H*-pyrido[2,3-*c*]carbazole (**9b**), Figure S7.7: HMBC of 10-fluoro-7*H*-pyrido[2,3-*c*]carbazole (**9b**), Figure S8.1: ^1^H NMR of 4-methyl-7*H*-pyrido[2,3-*c*]carbazolium iodide (**10**), Figure S8.2: ^13^C NMR of 4-methyl-7*H*-pyrido[2,3-*c*]carbazolium iodide (**10**), Figure S8.3: COSY of 4-methyl-7*H*-pyrido[2,3-*c*]carbazolium iodide (**10**), Figure S8.4: NOESY of 4-methyl-7*H*-pyrido[2,3-*c*]carbazolium iodide (**10**), Figure S8.5: HSQC of 4-methyl-7*H*-pyrido[2,3-*c*]carbazolium iodide (**10**), Figure S8.6: HMBC of 4-methyl-7*H*-pyrido[2,3-*c*]carbazolium iodide (**10**), Figure S9.1: ^1^H NMR of 4-methyl-11*H*-pyrido[3,2-*a*]carbazolium iodide (**21**), Figure S9.2: ^13^C NMR of 4-methyl-11*H*-pyrido[3,2-*a*]carbazolium iodide (**21**), Figure S9.3: COSY of 4-methyl-11*H*-pyrido[3,2-*a*]carbazolium iodide (**21**), Figure S9.4: NOESY of 4-methyl-11*H*-pyrido[3,2-*a*]carbazolium iodide (**21**), Figure S9.5: HSQC of 4-methyl-11*H*-pyrido[3,2-*a*]carbazolium iodide (**21**), Figure S9.6: HMBC of 4-methyl-11*H*-pyrido[3,2-*a*]carbazolium iodide (**21**).

## Sample Availability

Samples of the compounds are available from the authors.
