# Supplementary material for: Synthesis and Evaluation of the Tetracyclic Ring-System of Isocryptolepine and Regioisomers for Antimalarial, Antiproliferative and Antimicrobial Activities
Source: Molecules. 2021 May 30;26(11):3268. doi: 10.3390/molecules26113268 (PMC8198049; doi:10.3390/molecules26113268)
Supplement: Supplementary file 1 [file molecules-26-03268-s001.zip › molecules-1220471-supplementary.pdf]

Supplementary material

Synthesis and evaluation of the tetracyclic ring-system of isocryptolepine and regioisomers for antimalarial, antiproliferative and antimicrobial activities

Katja S. Håheim[a], Emil Lindbäck[a], Kah Ni Tan[b] , Marte Albrigtsen[c] , Ida T. Urdal Helgeland[a], Clémence Lauga[a], Théodora Matringe[a], Emily K. Kennedy[b], Jeanette H. Andersen[c], Vicky M. Avery[b,d,e] and Magne O. Sydnes[a*]

email: magne.o.sydnes@uis.no

Enclosed is the ^{1}H-, ^{13}C-, where relevant, ^{19}F-NMR and 2D NMR (COSY, NOESY, HMBC and HSQC) spectra for compounds **3b**, **3c**, **7c**, **8a**, **8b**, **9a**, **9b**, **10** and **21**.

5-Ethyl-5*H*-indolo[3,2-*c*]quinoline (3b)

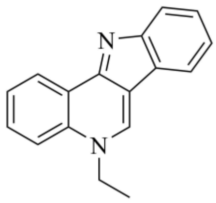

^{1}H NMR (400 MHz, DMSO-d_6)

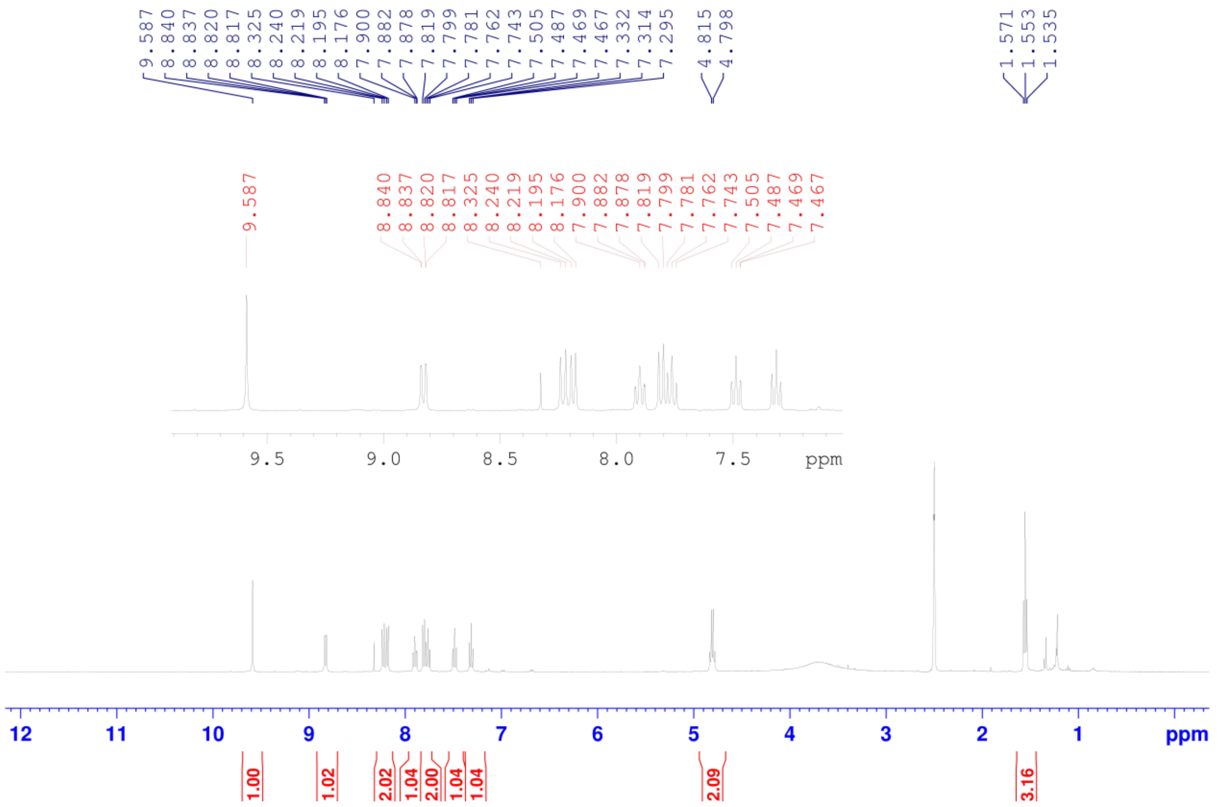

^{13}C NMR (100 MHz, DMSO-d_6)

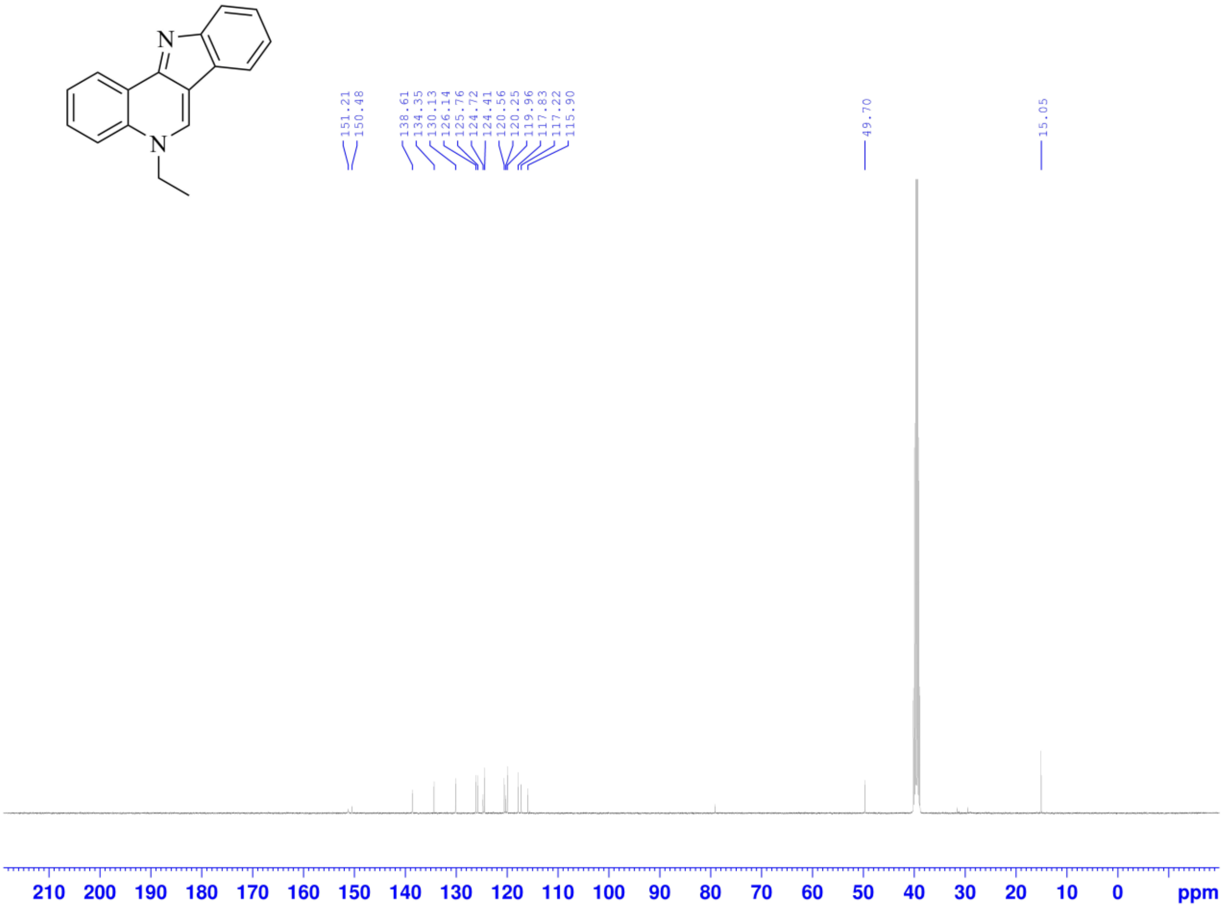

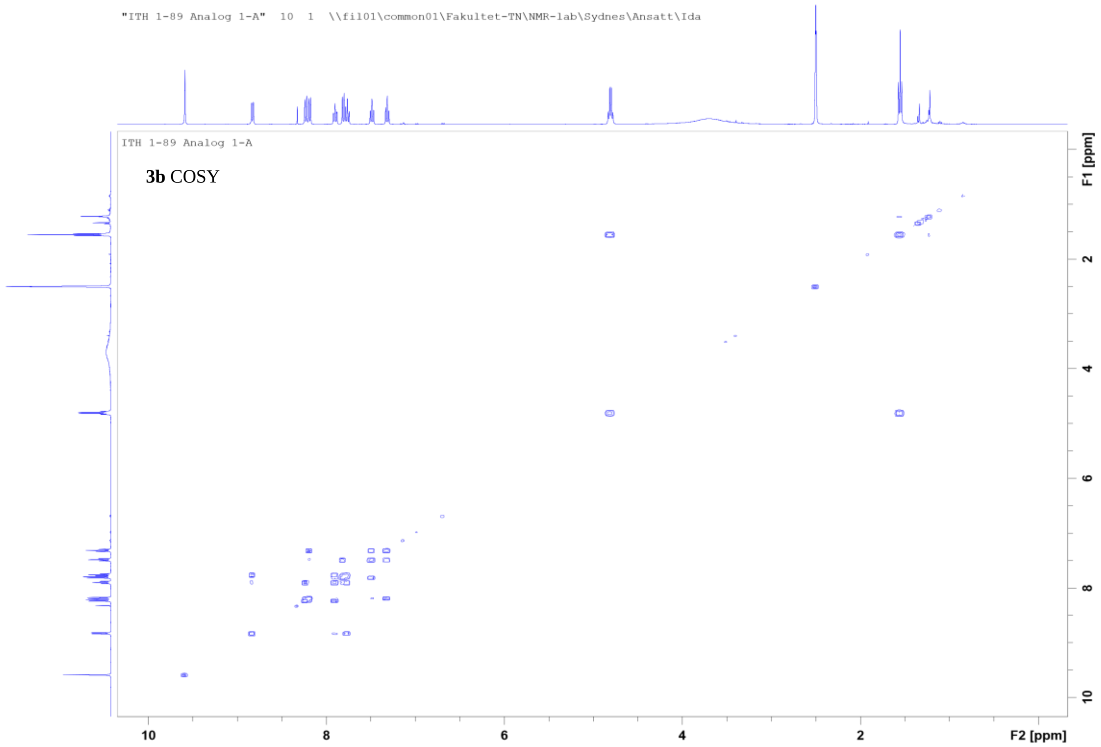

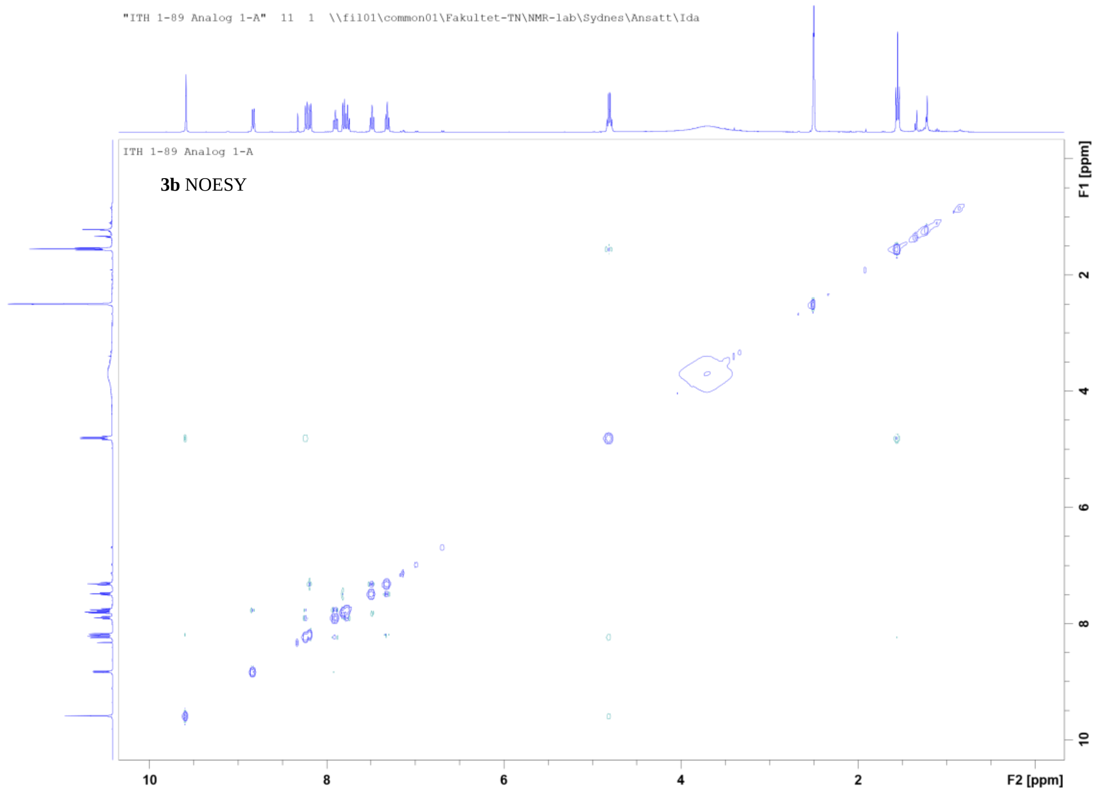
"ITH 1-89 Analog 1-A" 11 1 \\fil01\common01\Fakultet-TN\NMR-lab\Sydnes\Ansatt\Ida
ITH 1-89 Analog 1-A
3b NOESY
F1 [ppm]
F2 [ppm]

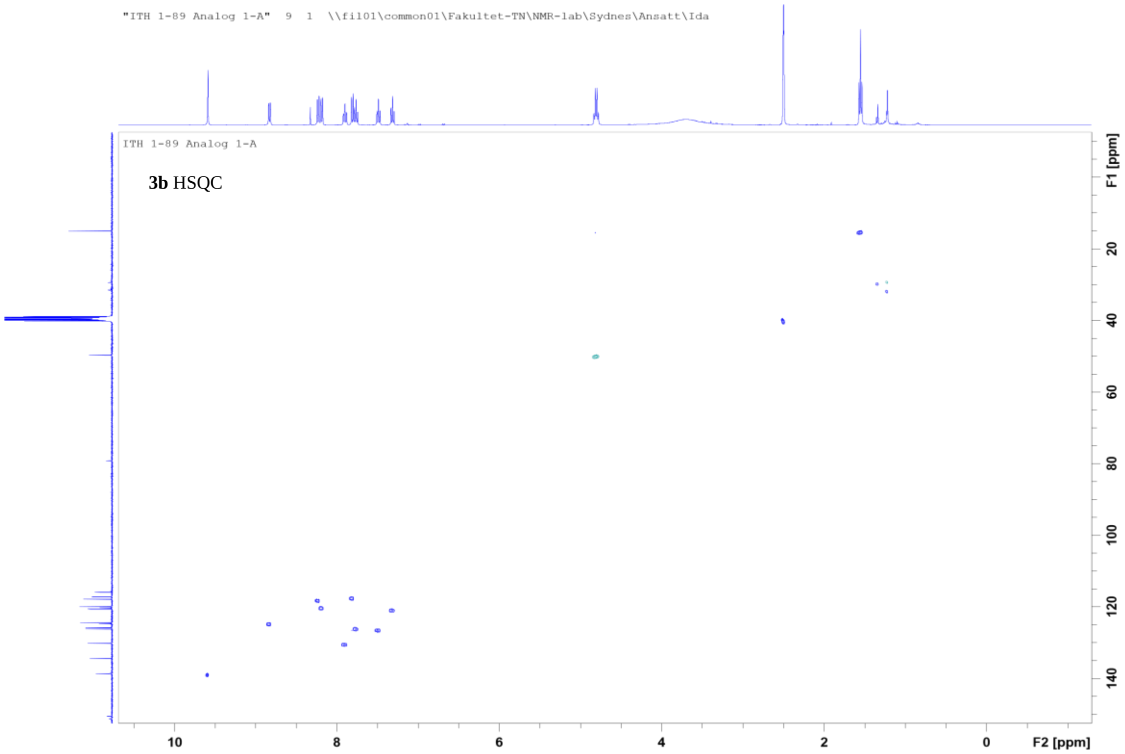
"ITH 1-89 Analog 1-A" 9 1 \\fil01\common01\Fakultet-TN\NMR-lab\Sydnes\Ansatt\Ida
ITH 1-89 Analog 1-A
3b HSQC
F1 [ppm]
F2 [ppm]

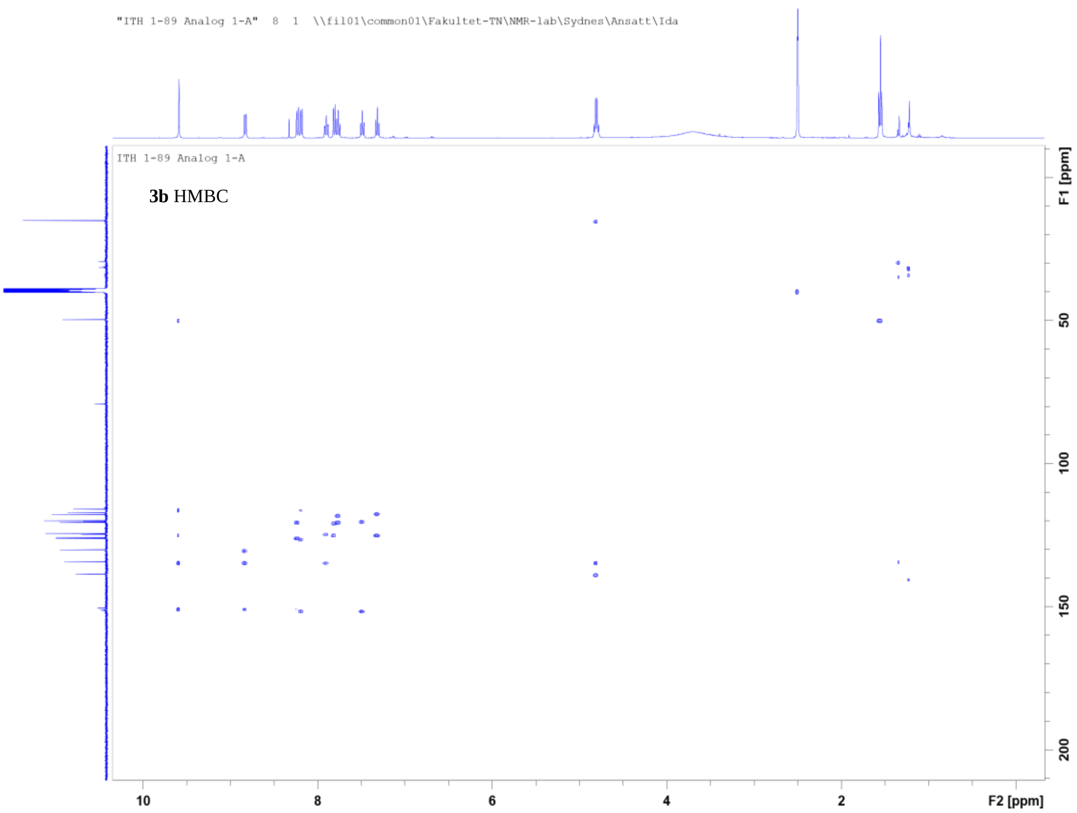
"ITH 1-89 Analog 1-A" 8 1 \\fil01\common01\Fakultet-TN\NMR-lab\Sydnes\Ansatt\Ida
ITH 1-89 Analog 1-A
3b HMBC
F1 [ppm]
50
100
150
200
10
8
6
4
2
F2 [ppm]

5-Allyl-5*H*-indolo[3,2-*c*]quinoline (3c)

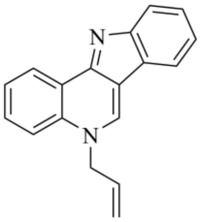

^{1}H NMR (400 MHz, DMSO-d_6)

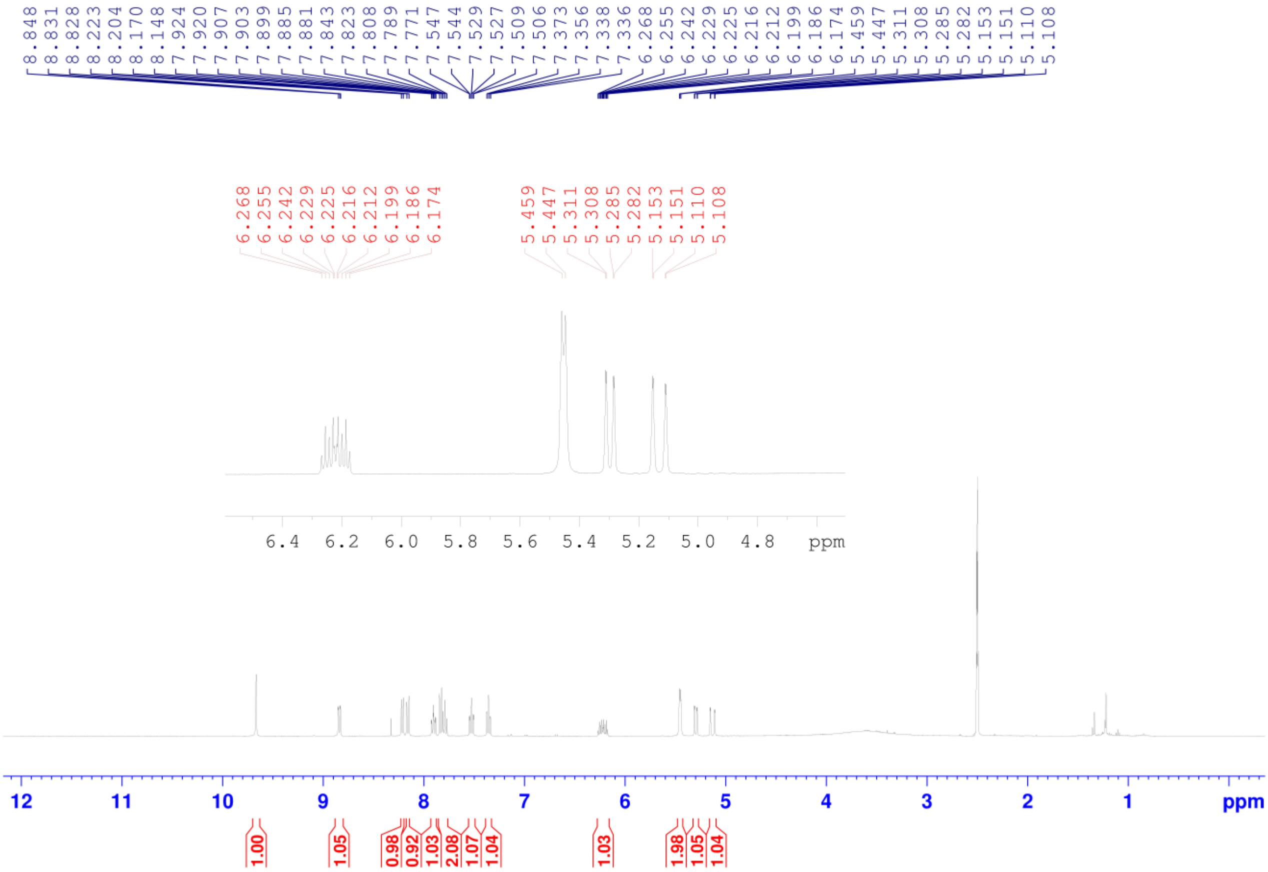

^{13}C NMR (100 MHz, DMSO-d_6)

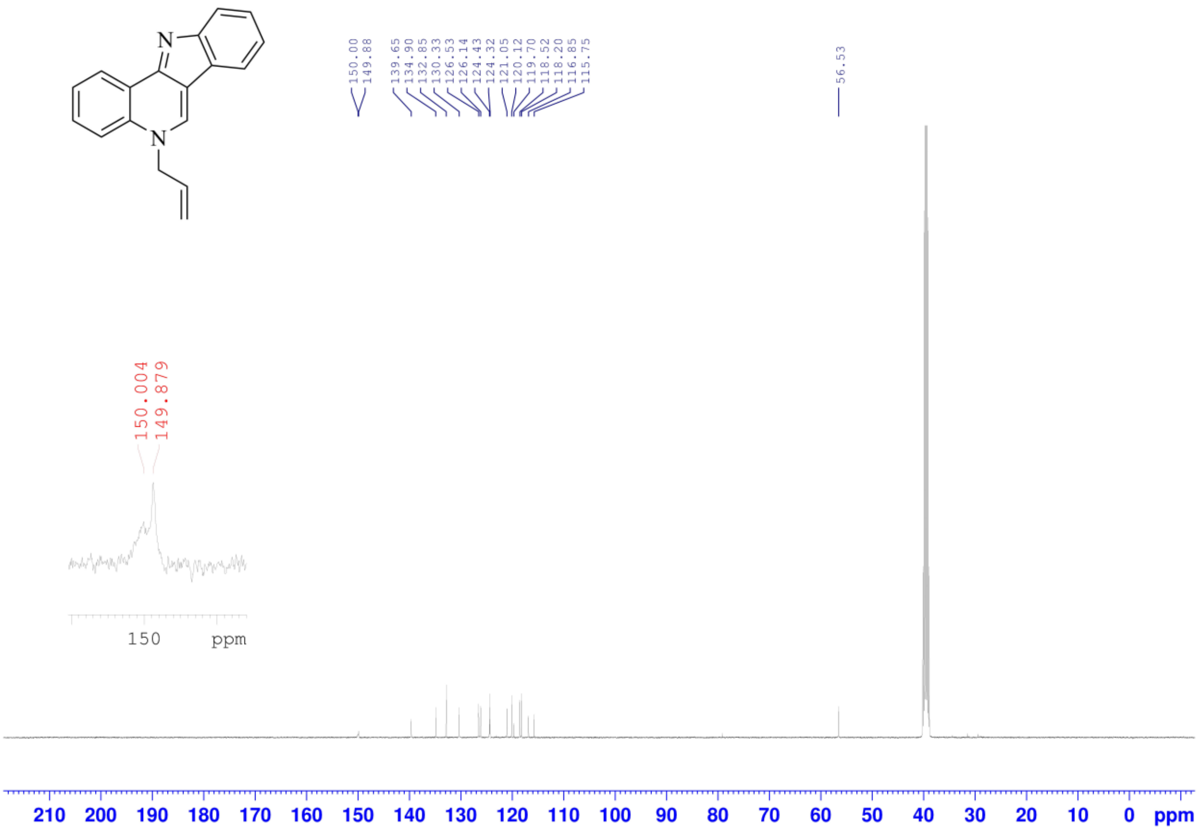

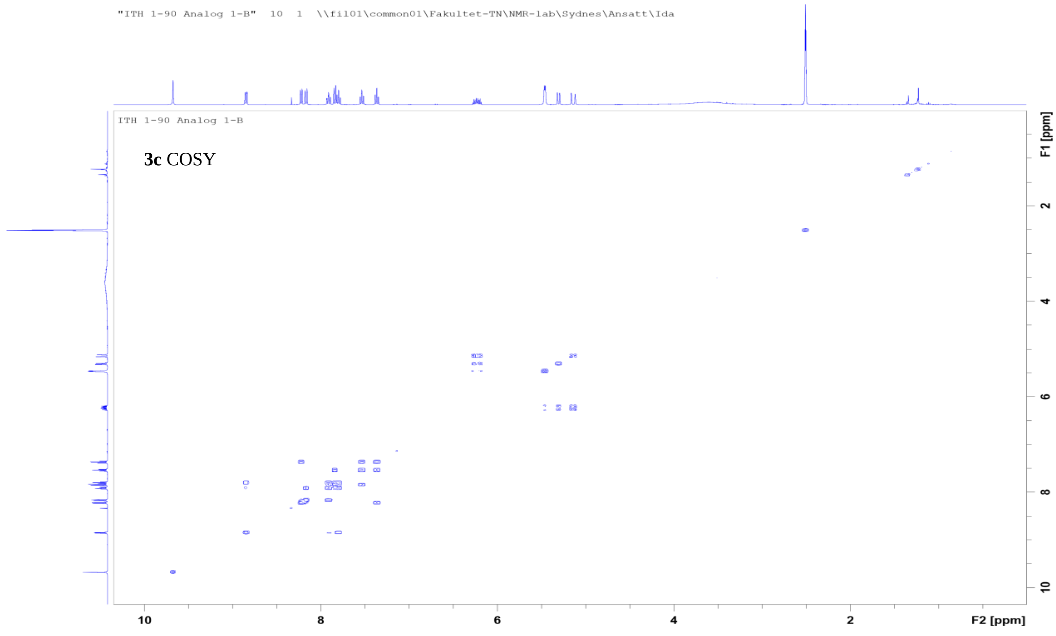

3c COSY

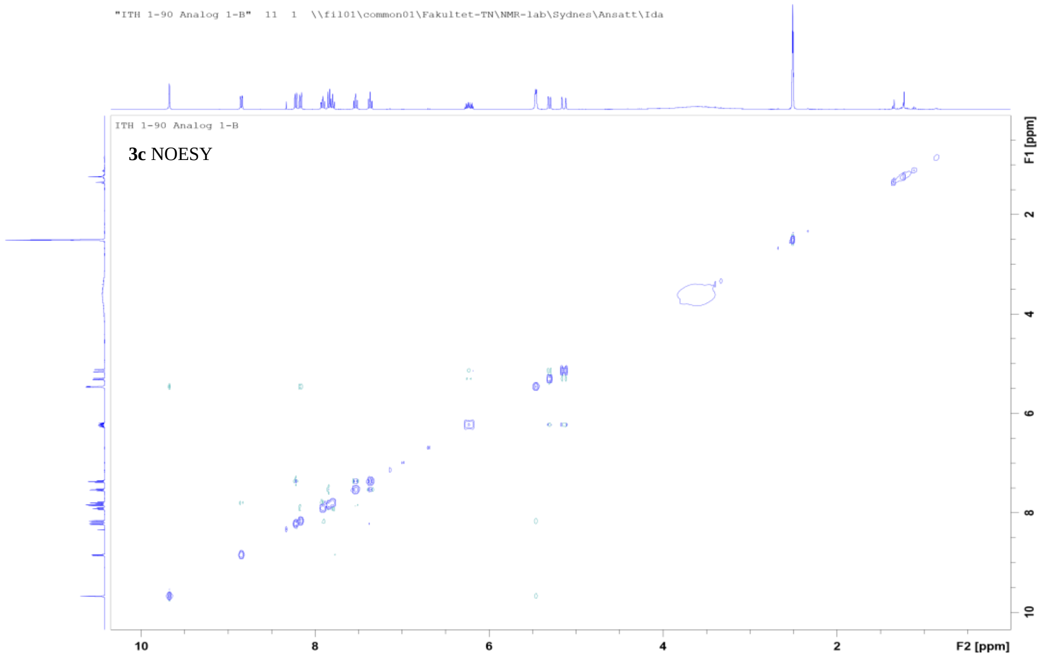
"ITH 1-90 Analog 1-B" 11 1 \\fi101\common01\Fakultet-TN\NMR-lab\Sydnes\Ansatt\Ida
ITH 1-90 Analog 1-B
3c NOESY
F1 [ppm]
F2 [ppm]

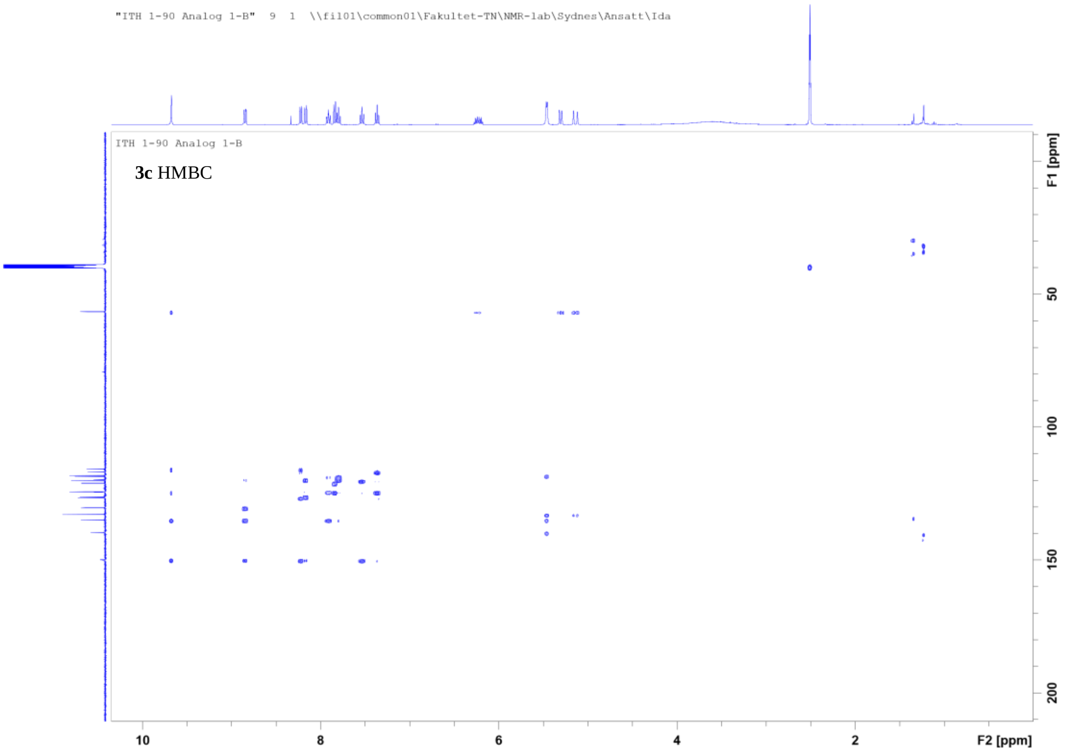
"ITH 1-90 Analog 1-B" 9 1 \\fi101\common01\Fakultet-TN\NMR-lab\Sydnes\Ansatt\Ida
ITH 1-90 Analog 1-B
3c HMBC
F1 [ppm]
F2 [ppm]

4-Fluoro-2-(quinolin-5-yl)aniline (7c)

^{1}H NMR (400 MHz, CD_2Cl_2)

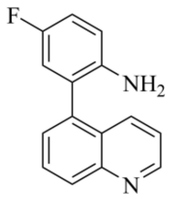

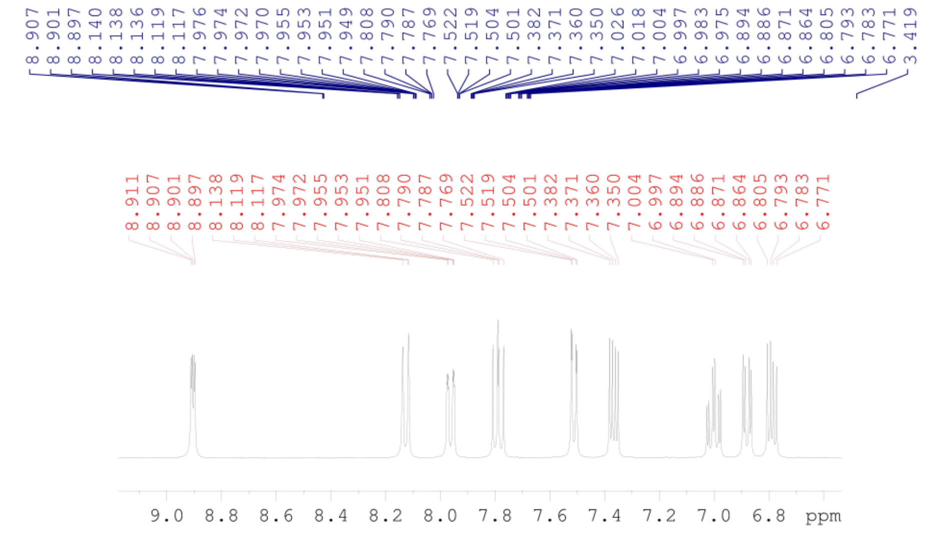

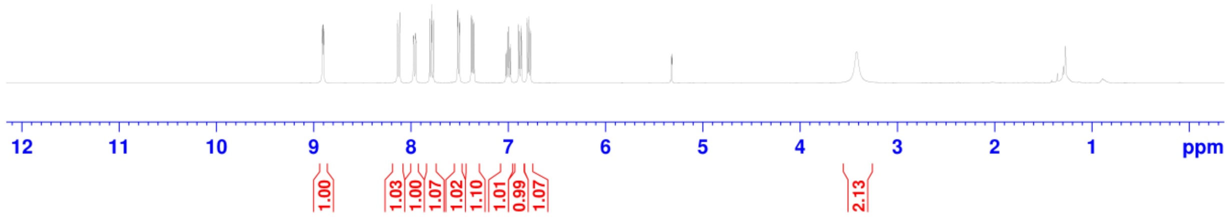

^{13}C NMR (100 MHz, CD_2Cl_2)

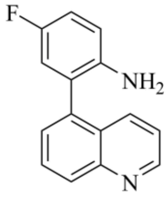

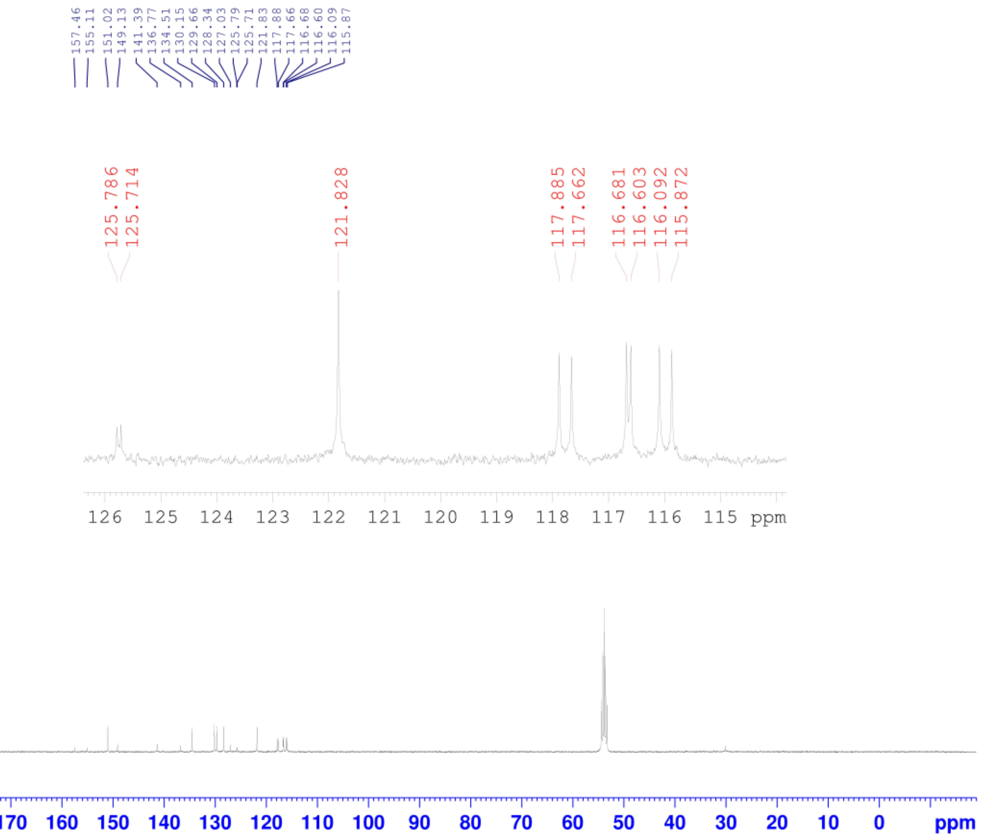

^{19}F NMR (376 MHz, CD_2Cl_2)

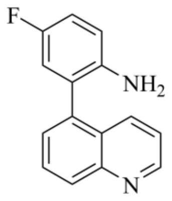

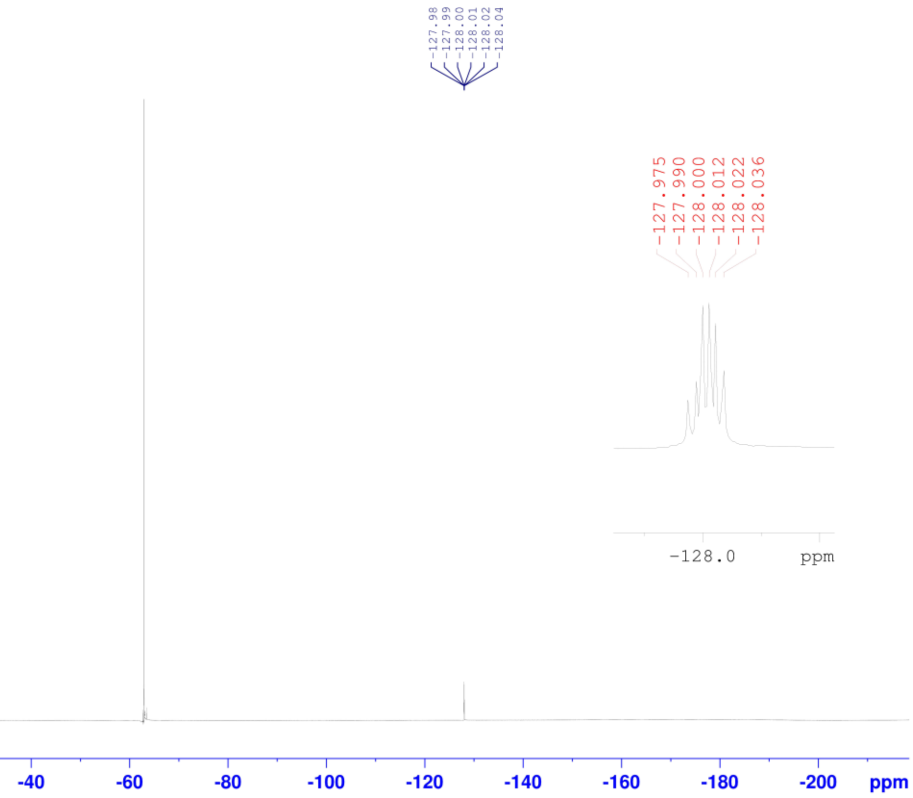

4-Methyl-4*H*-pyrido[4,3,2-*gh*]phenanthridine (8a)

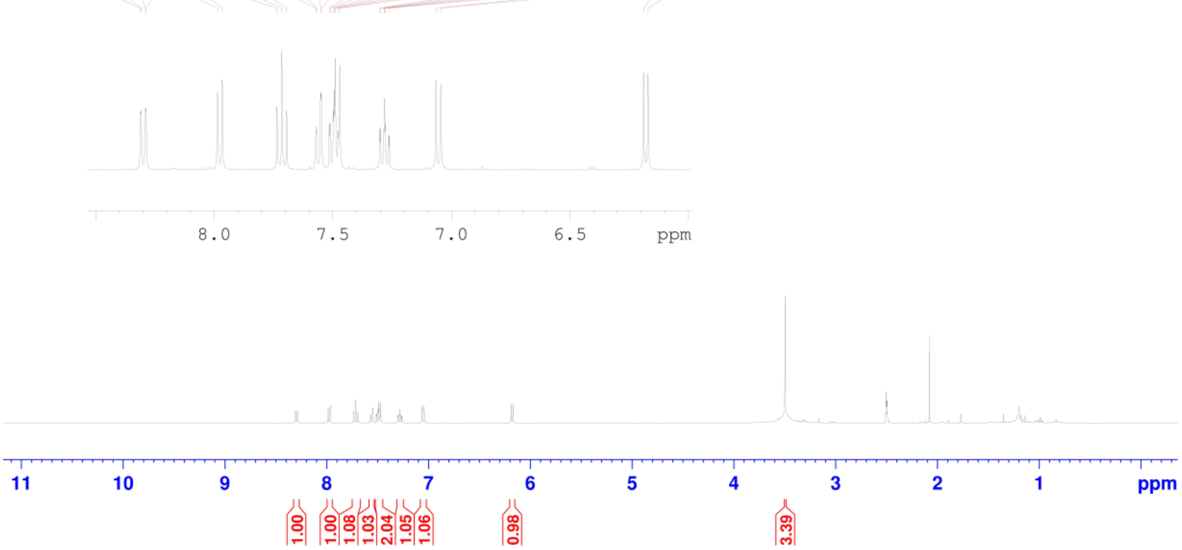

^{1}H NMR (400 MHz, DMSO-d_6)

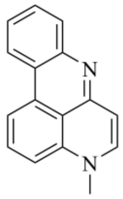

^{13}C NMR (100 MHz, DMSO-d_6)

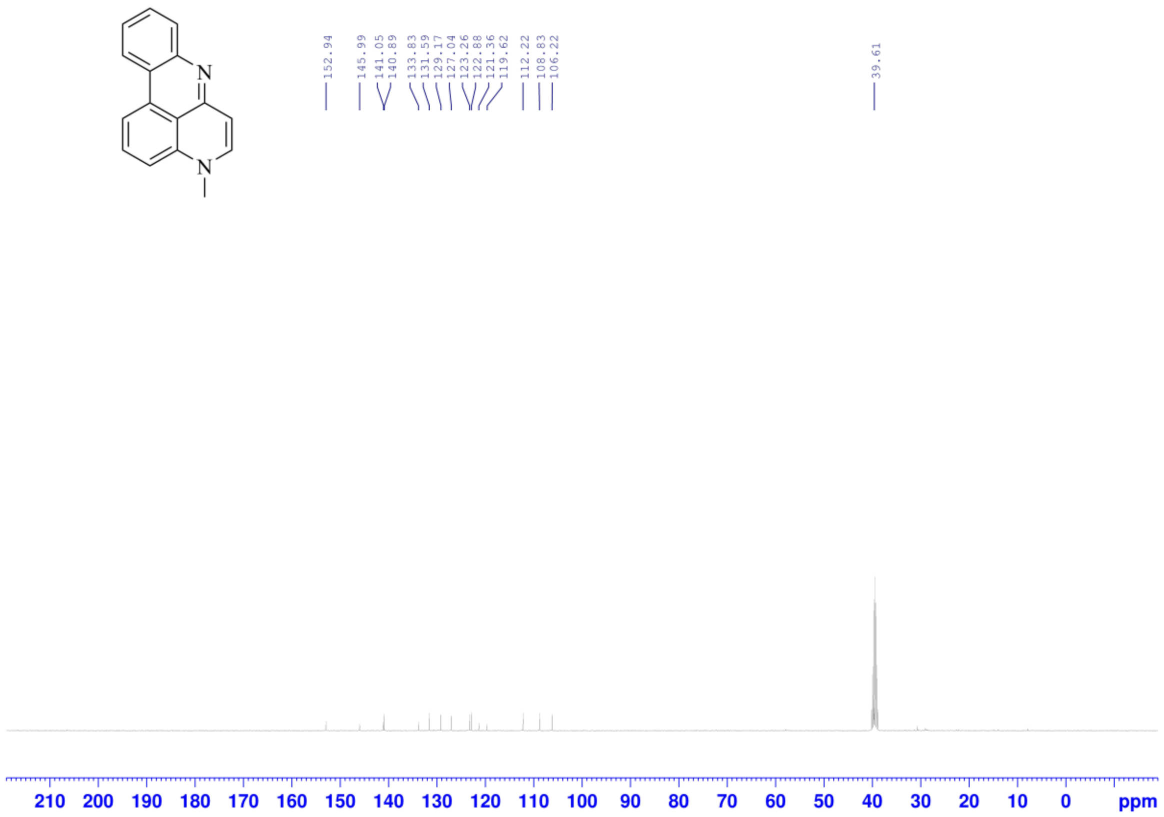

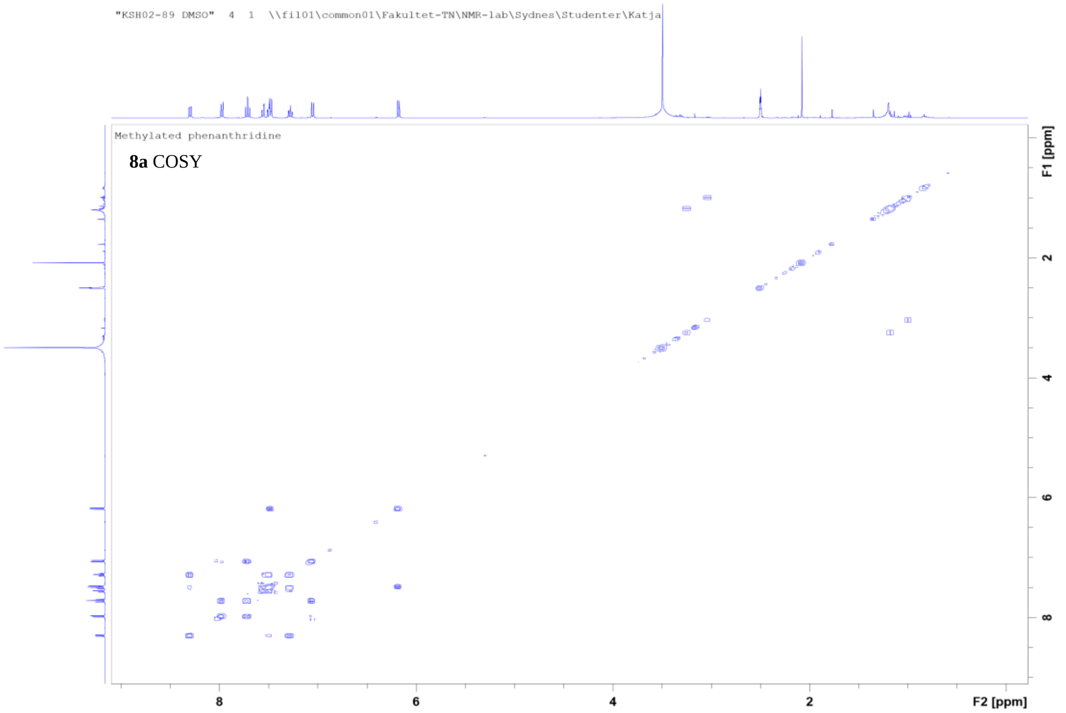

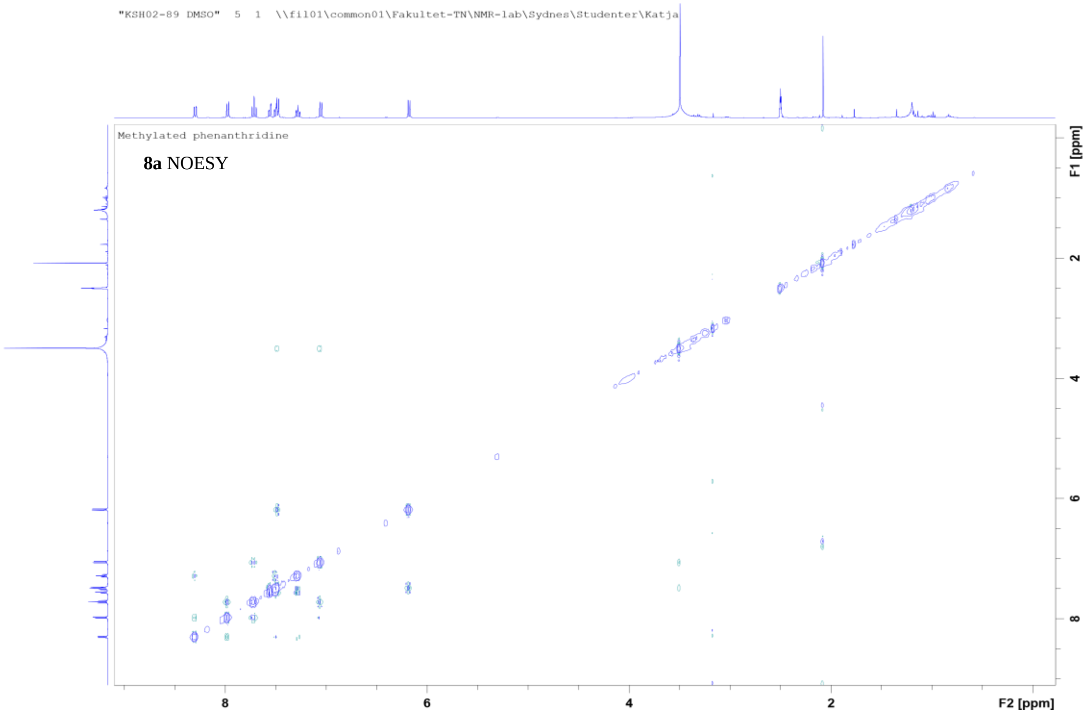
"KSH02-89 DMSO" 5 1 \\fil01\common01\Fakultet-TN\NMR-lab\Sydnes\Studenter\Katja
Methylated phenanthridine
8a NOESY
F1 [ppm]
F2 [ppm]

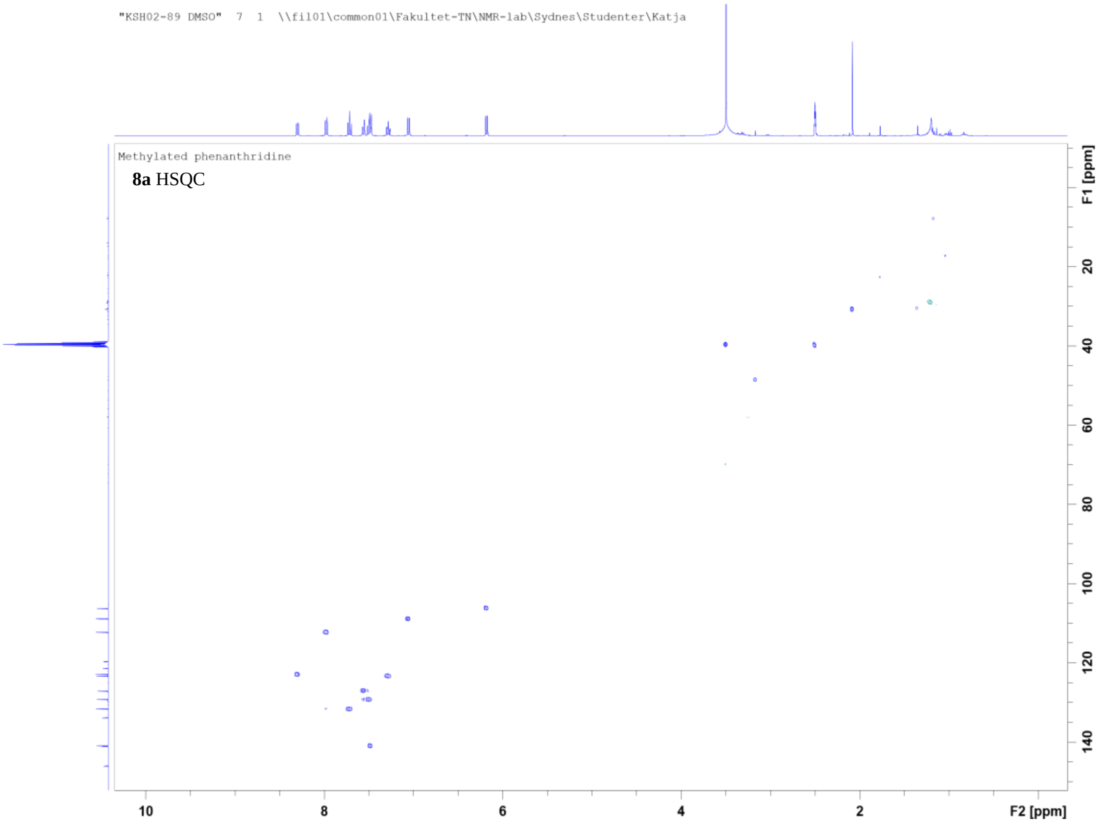
"KSH02-89 DMSO" 7 1 \\fil01\common01\Fakultet-TN\NMR-lab\Sydnes\Studenter\Katja
Methylated phenanthridine
8a HSQC
F1 [ppm]
F2 [ppm]

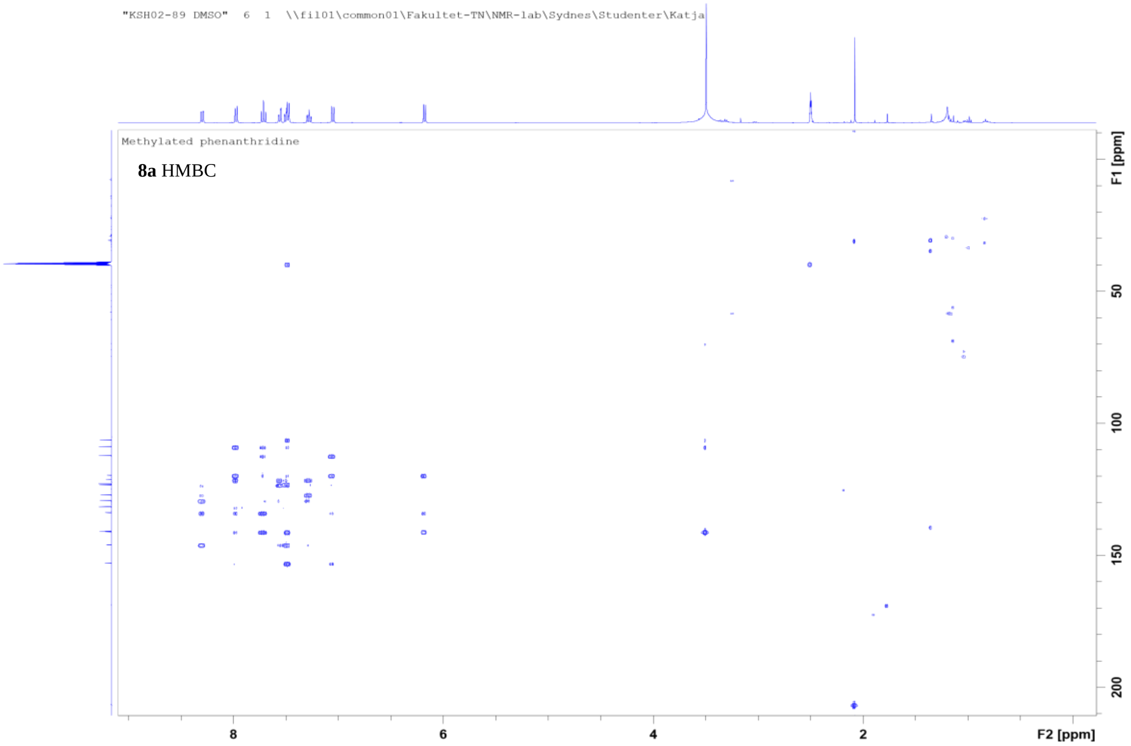
"KSH02-89 DMSO" 6 1 \\fil01\common01\Fakultet-TN\NMR-lab\Sydnes\Studenter\Katja
Methylated phenanthridine
8a HMBC
F1 [ppm]
50
100
150
200
8
6
4
2
F2 [ppm]

6-Methoxy-4-methyl-4*H*-pyrido[4,3,2-*gh*]phenanthridine (8b)

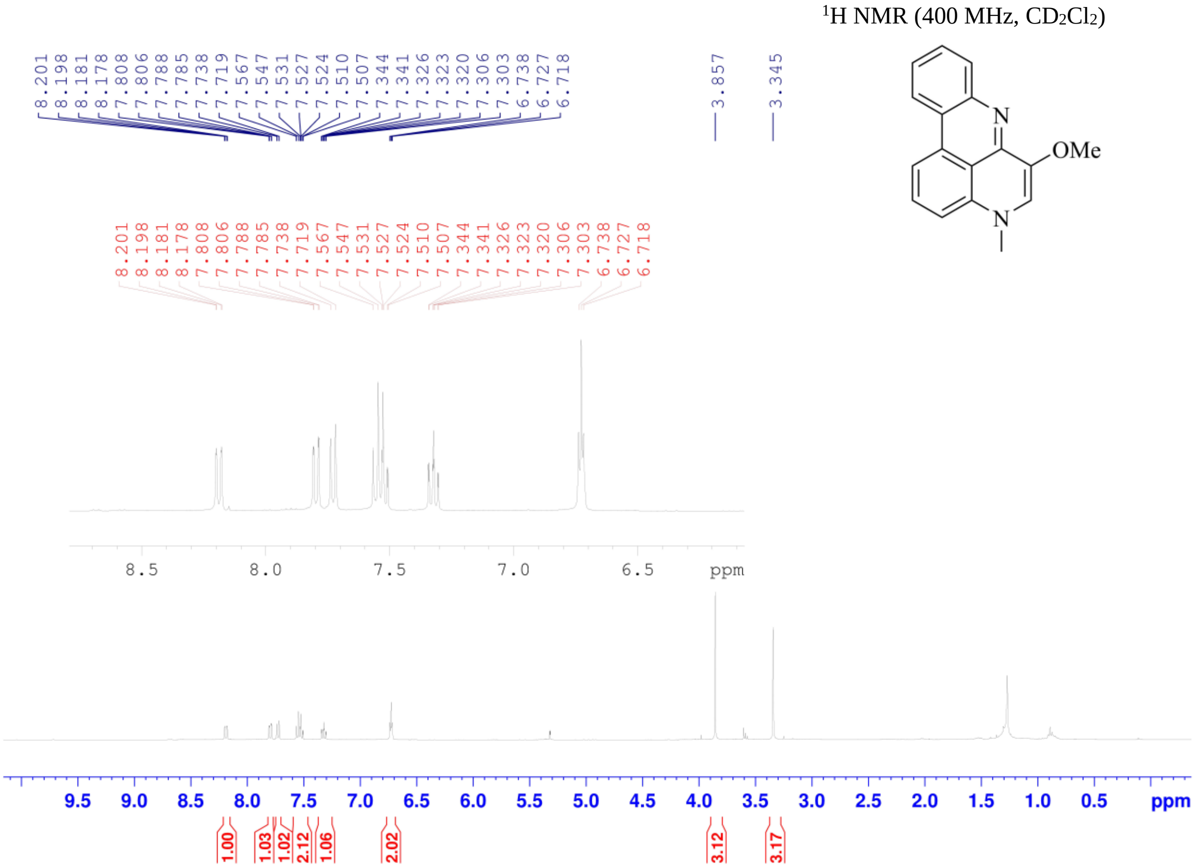

^{13}C NMR (100 MHz, CD_2Cl_2)

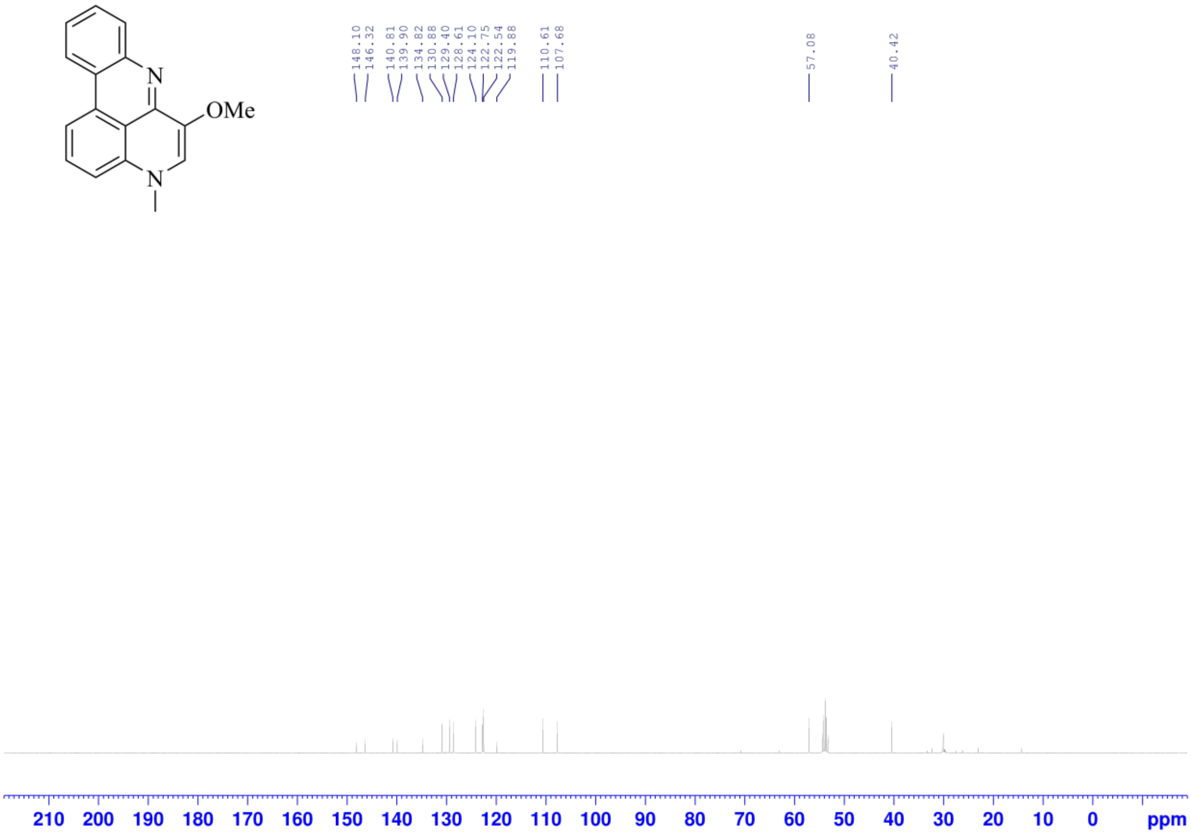

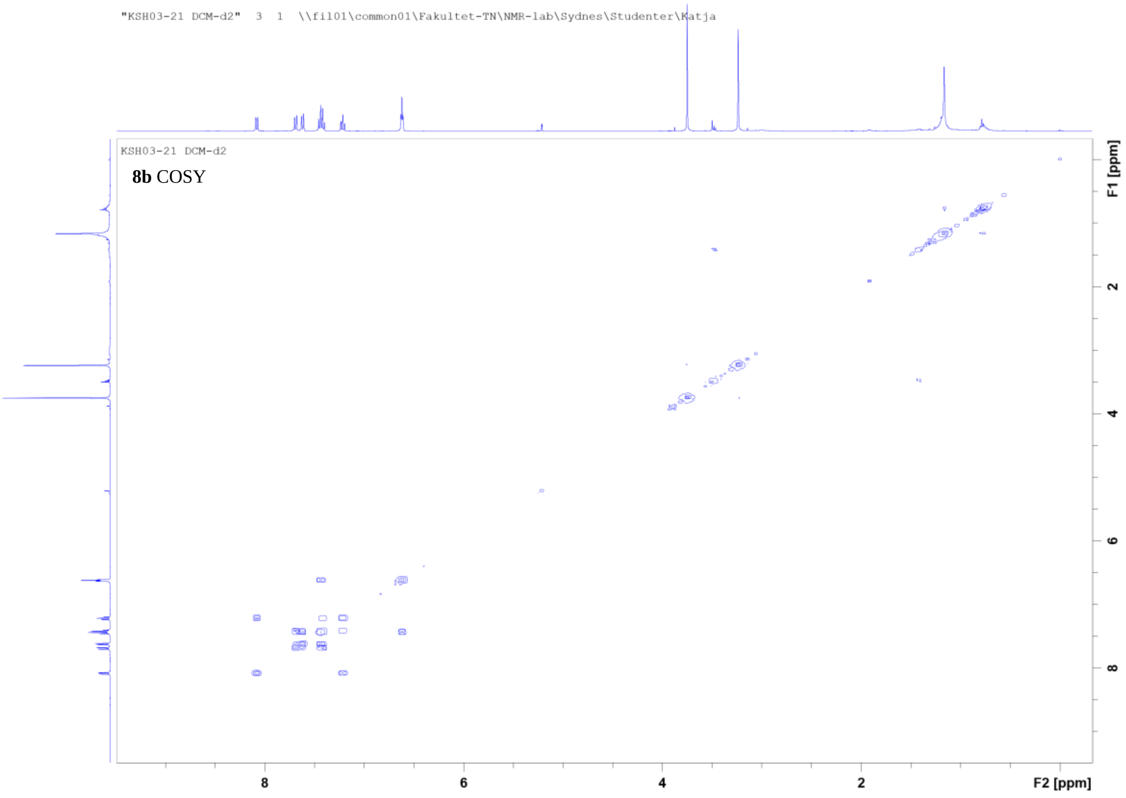

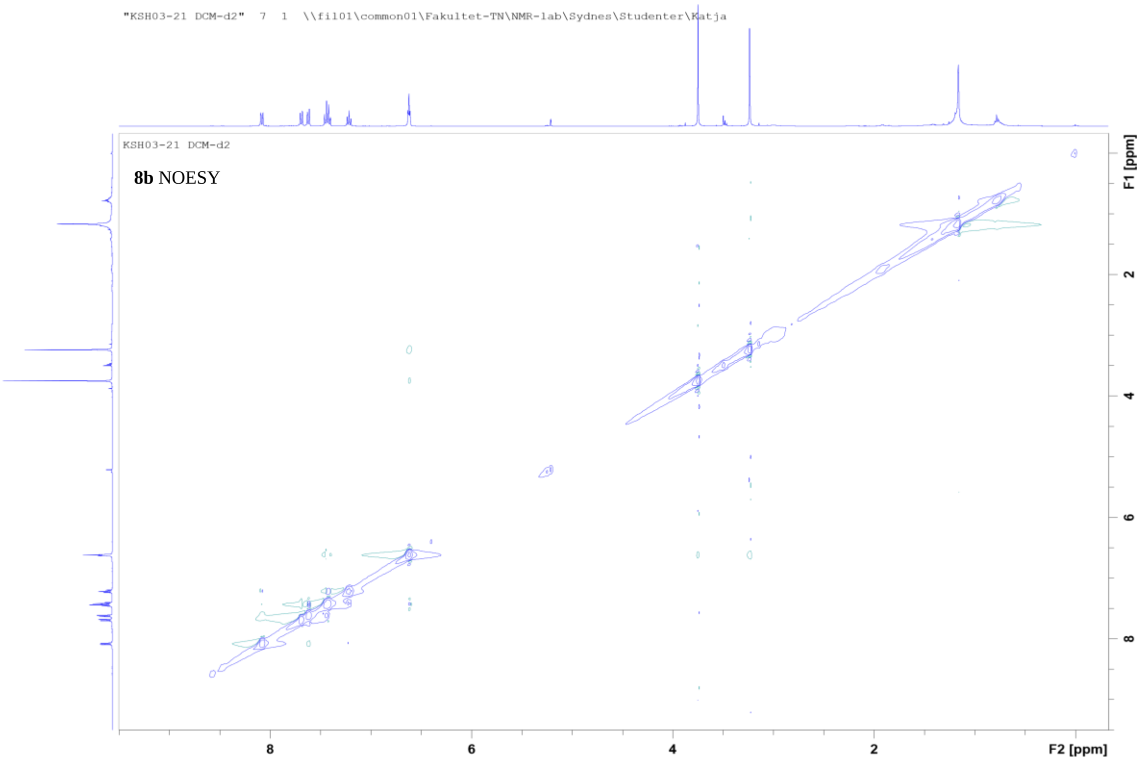
"KSH03-21 DCM-d2" 7 1 \\fi101\common01\Fakultet-TN\NMR-lab\Sydnes\Studenter\Katja
KSH03-21 DCM-d2
8b NOESY
F1 [ppm]
F2 [ppm]

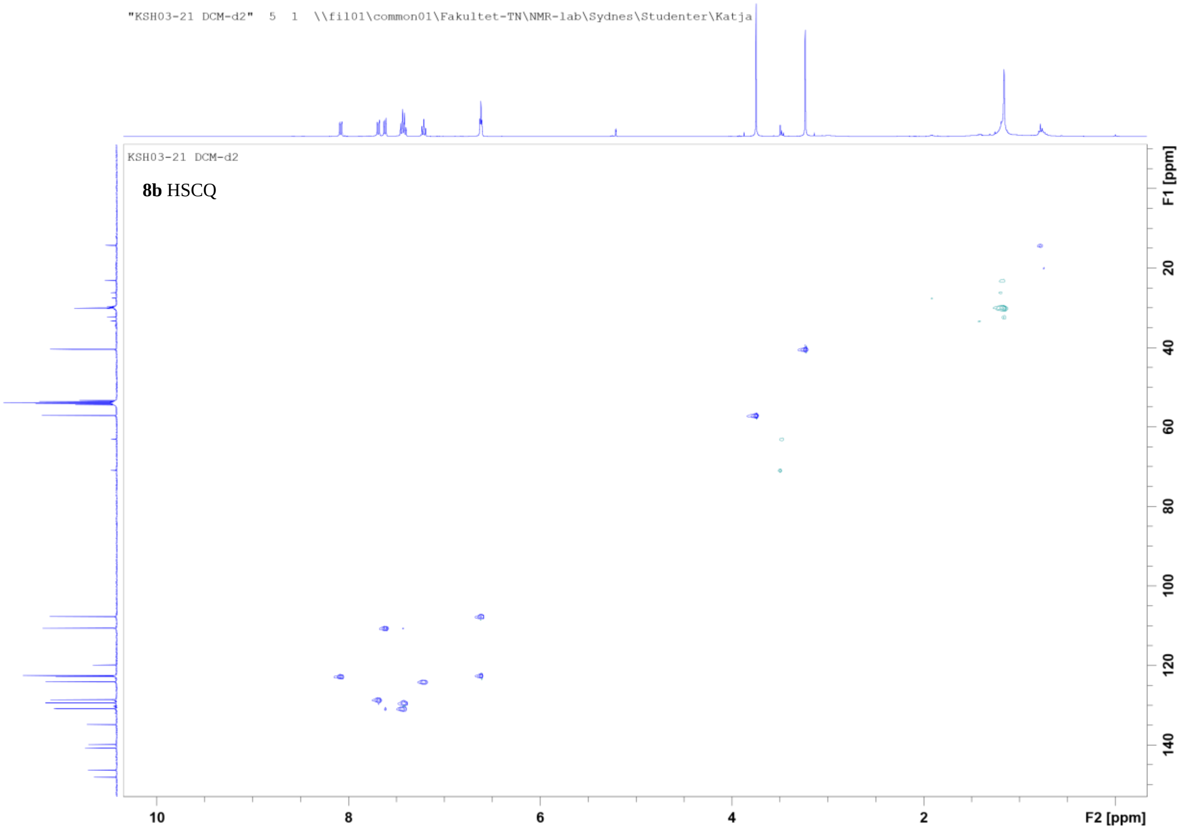
"KSH03-21 DCM-d2" 5 1 \\fi101\common01\Fakultet-TN\NMR-lab\Sydnes\Studenter\Katja
KSH03-21 DCM-d2
8b HSCQ
F1 [ppm]
F2 [ppm]

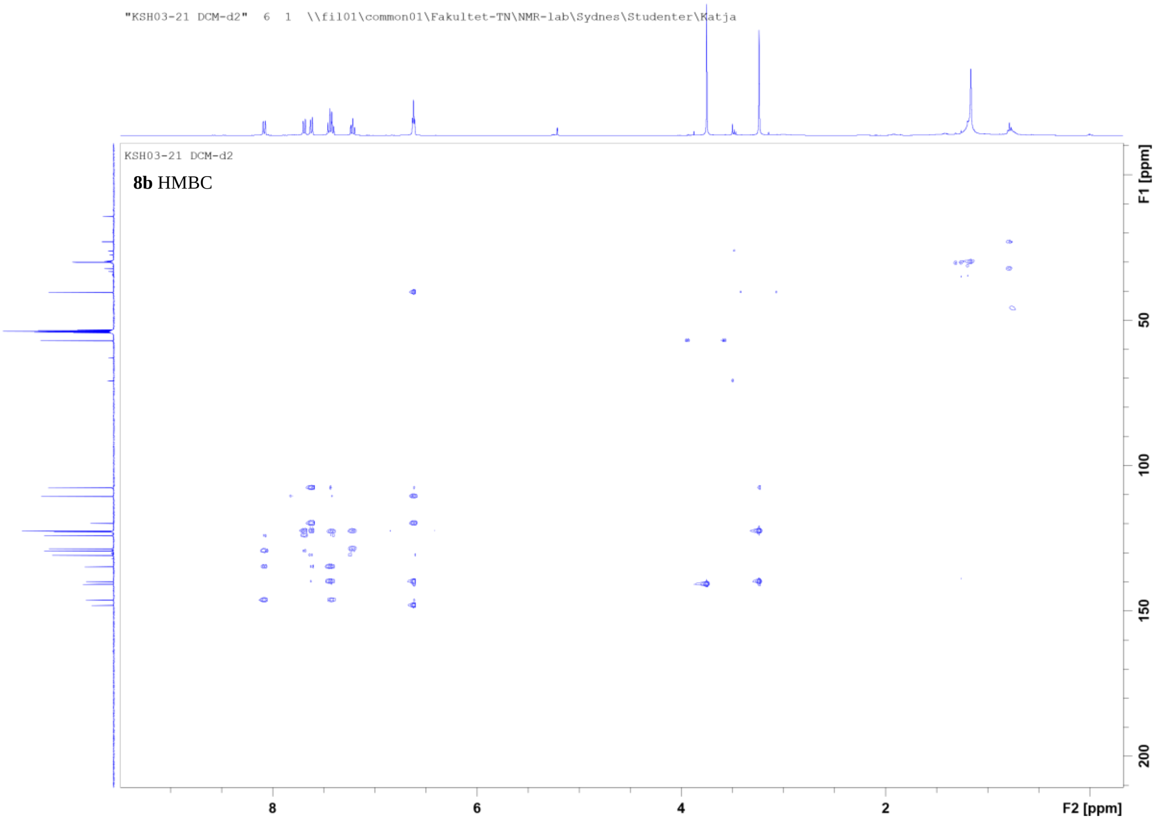
"KSH03-21 DCM-d2" 6 1 \\fil01\common01\Fakultet-TN\NMR-lab\Sydnes\Studenter\Katja
KSH03-21 DCM-d2
8b HMBC
F1 [ppm]
50
100
150
200
8
6
4
2
F2 [ppm]

7*H*-Pyrido[2,3-*c*]carbazole (9a)

^{1}H NMR (400 MHz, DMSO-d_6)

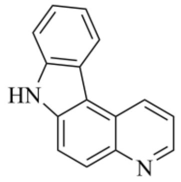

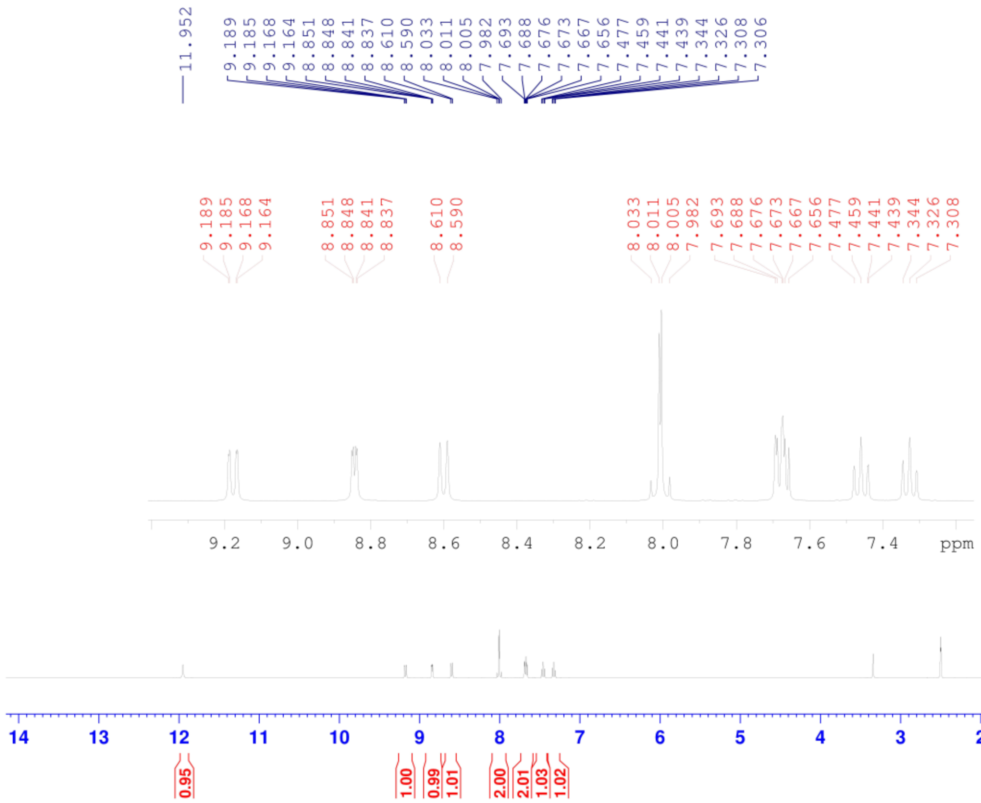

^{13}C NMR (100 MHz, DMSO-d_6)

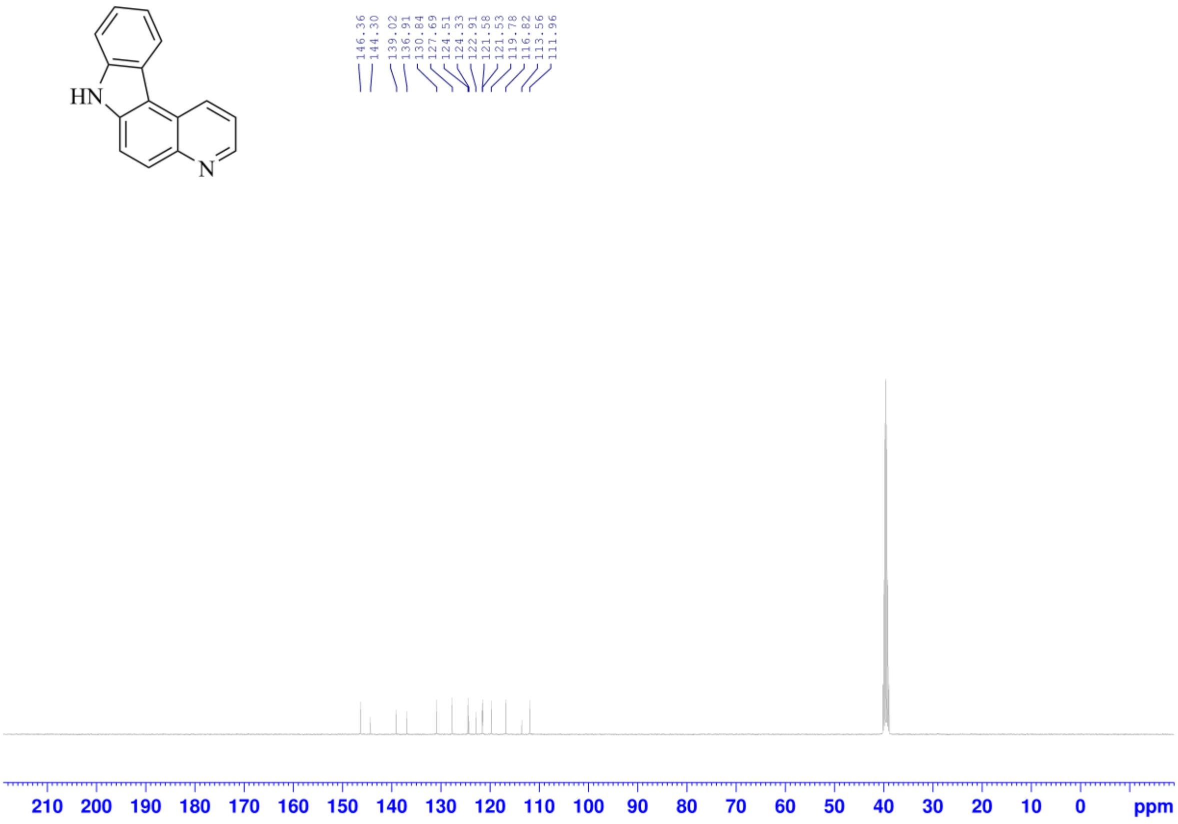

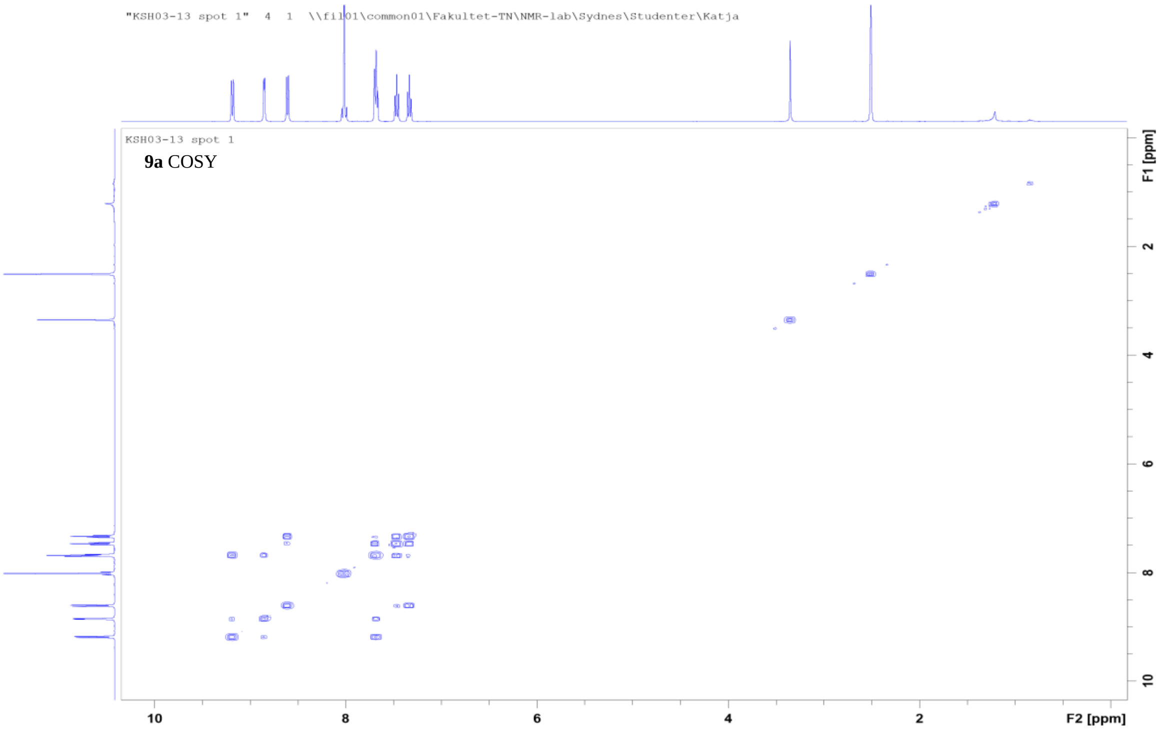

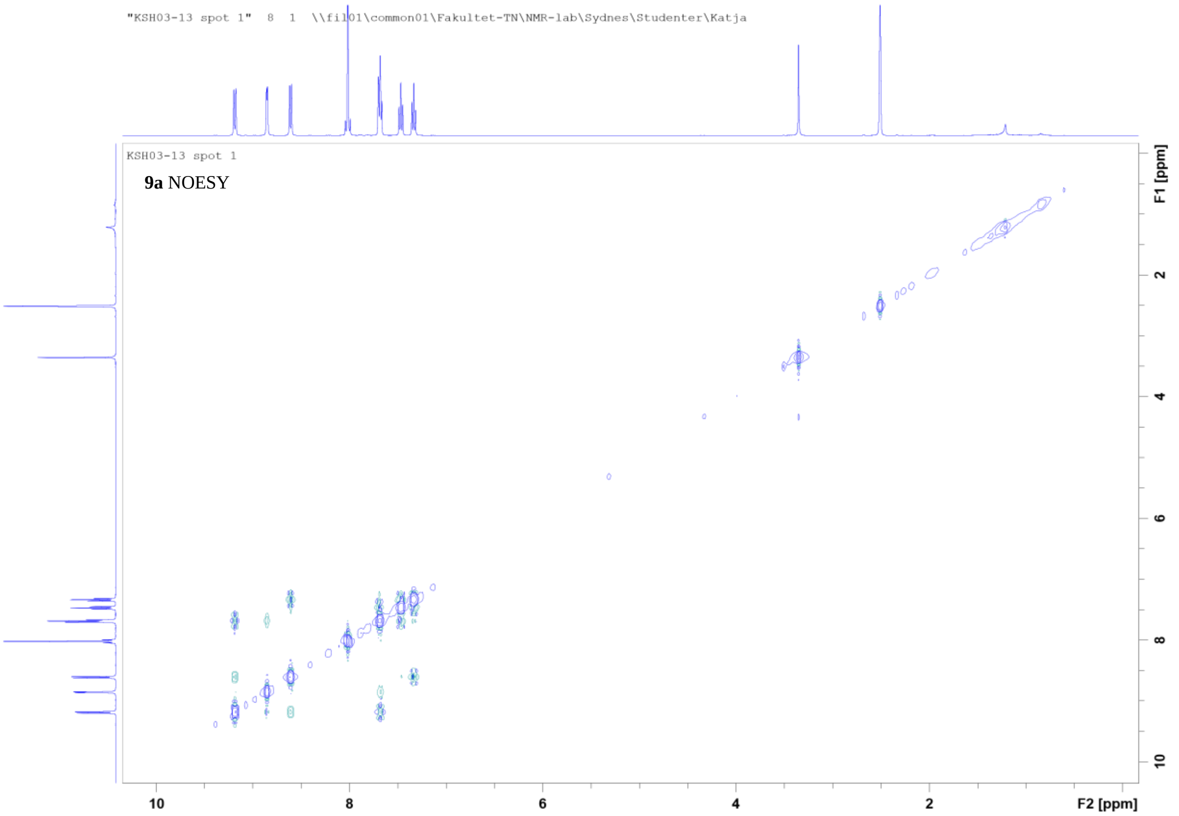
"KSH03-13 spot 1" 8 1 \\fil01\common01\Fakultet-TN\NMR-lab\Sydnes\Studenter\Katja
KSH03-13 spot 1
9a NOESY
F1 [ppm]
F2 [ppm]

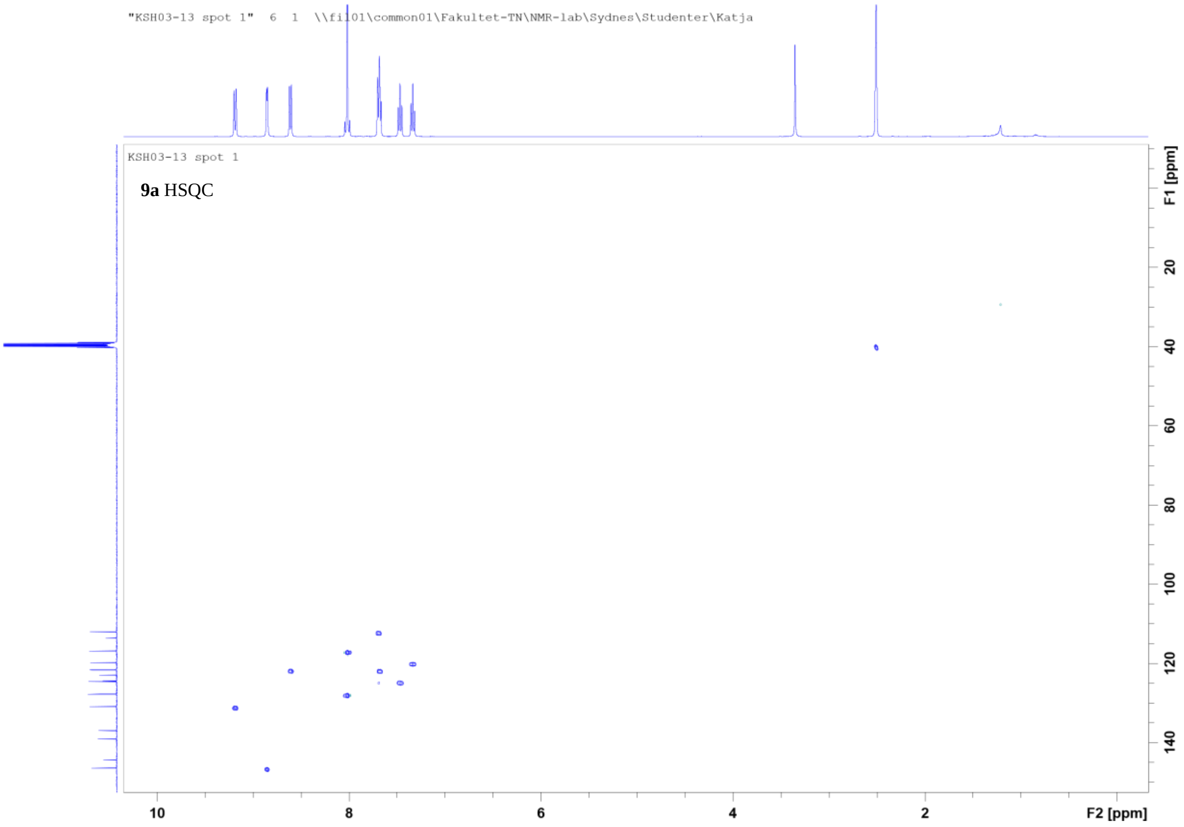
"KSH03-13 spot 1" 6 1 \\fil01\common01\Fakultet-TN\NMR-lab\Sydnes\Studenter\Katja
KSH03-13 spot 1
9a HSQC
F1 [ppm]
F2 [ppm]

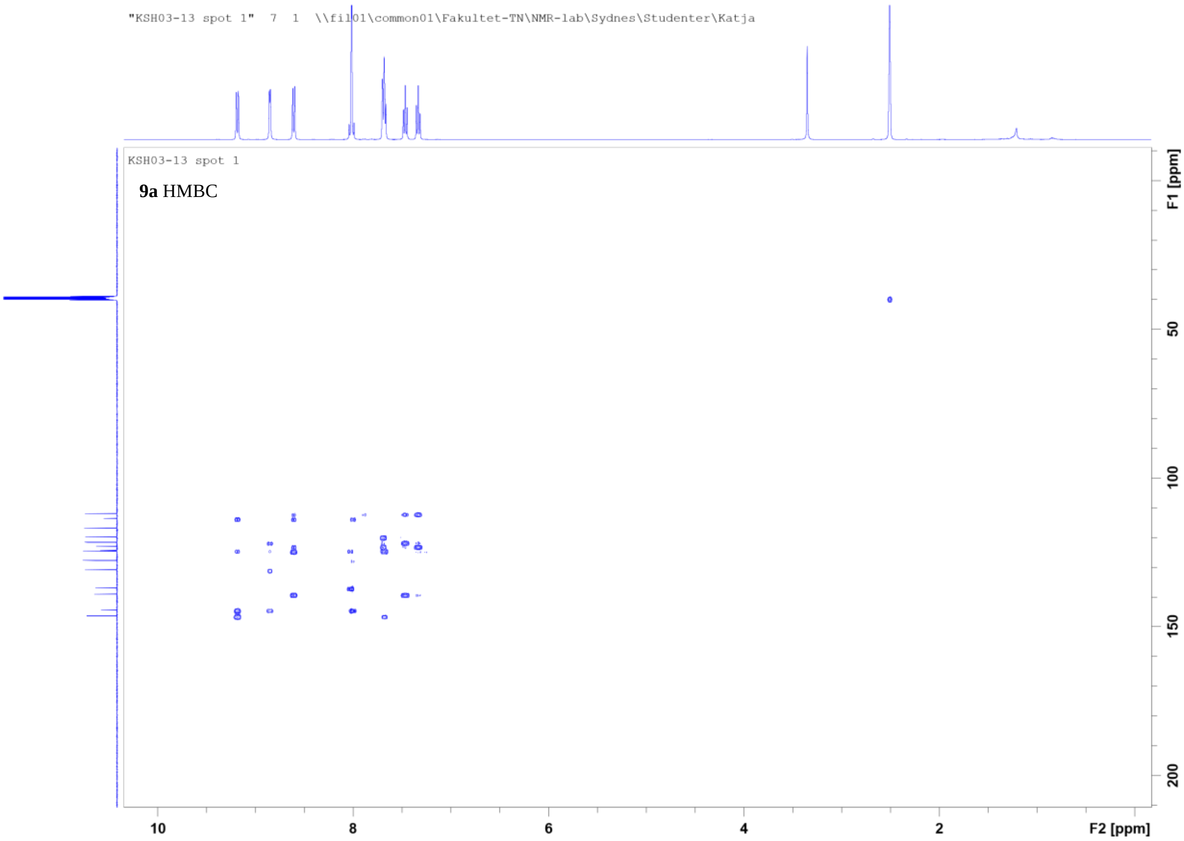
"KSH03-13 spot 1" 7 1 \\fil01\common01\Fakultet-TN\NMR-lab\Sydnes\Studenter\Katja
KSH03-13 spot 1
9a HMBC
F1 [ppm]
50
100
150
200
10
8
6
4
2
F2 [ppm]

10-Fluoro-7*H*-pyrido[2,3-*c*]carbazole (9b)

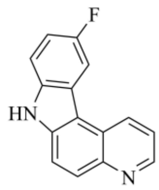

^{1}H NMR (400 MHz, CD_3OD)

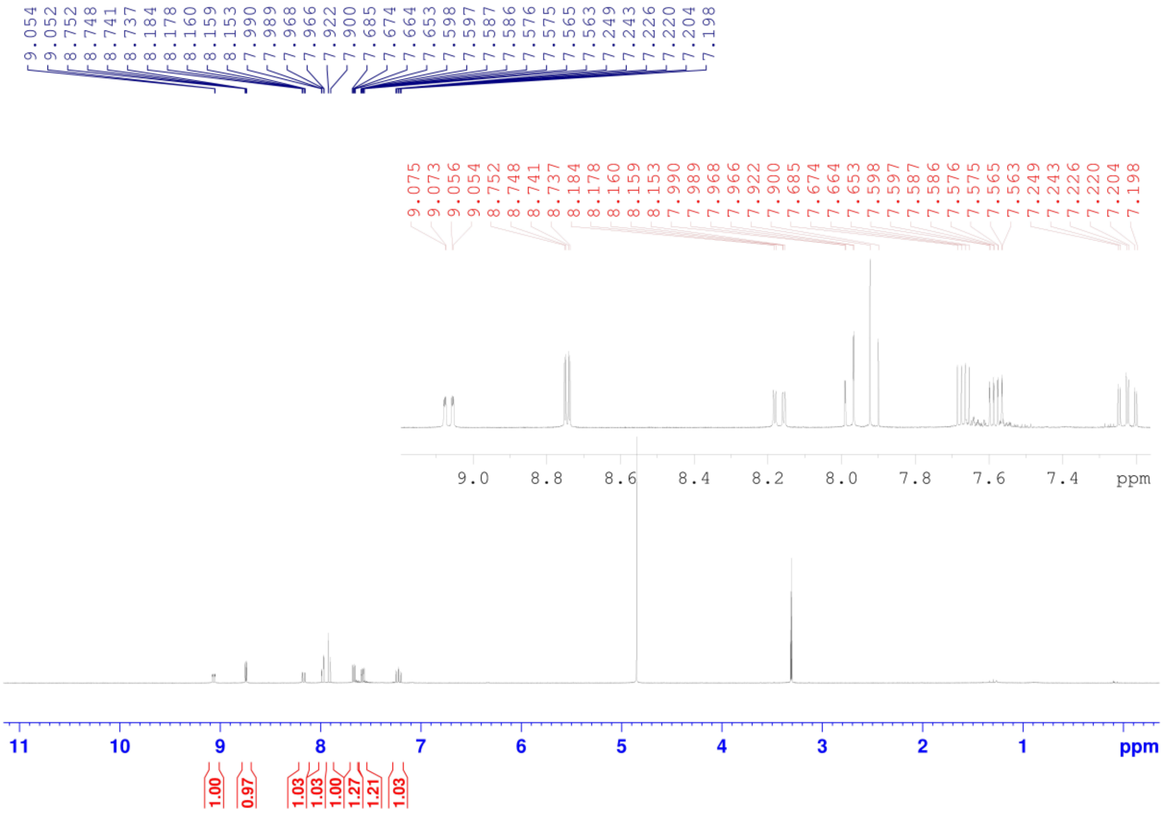

^{13}C NMR (100 MHz, CD_3OD)

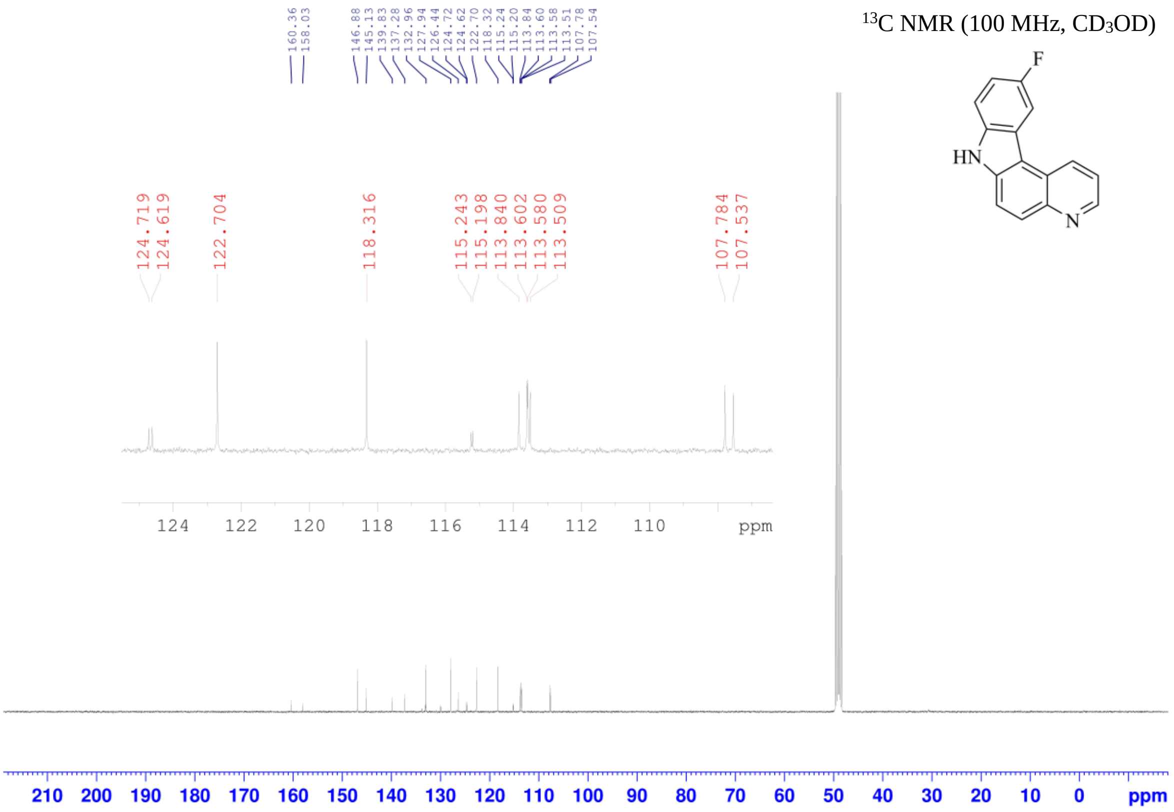

^{19}F NMR (376 MHz, CD_3OD)

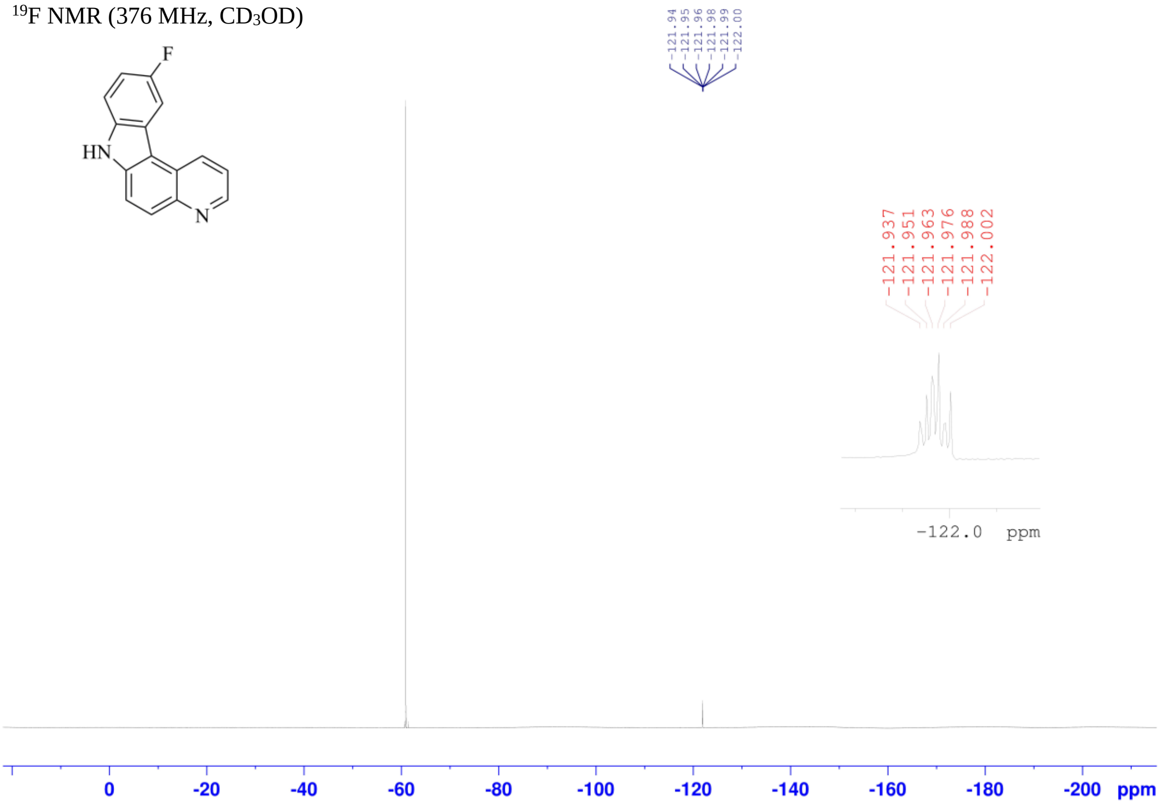

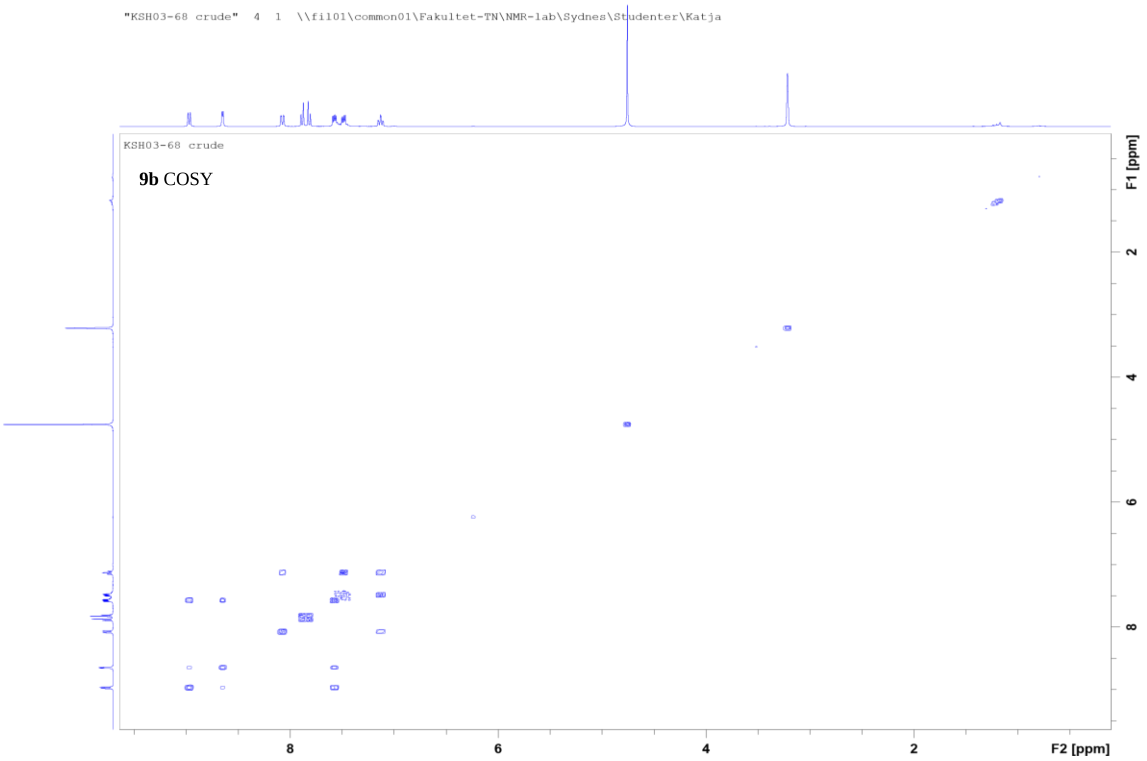

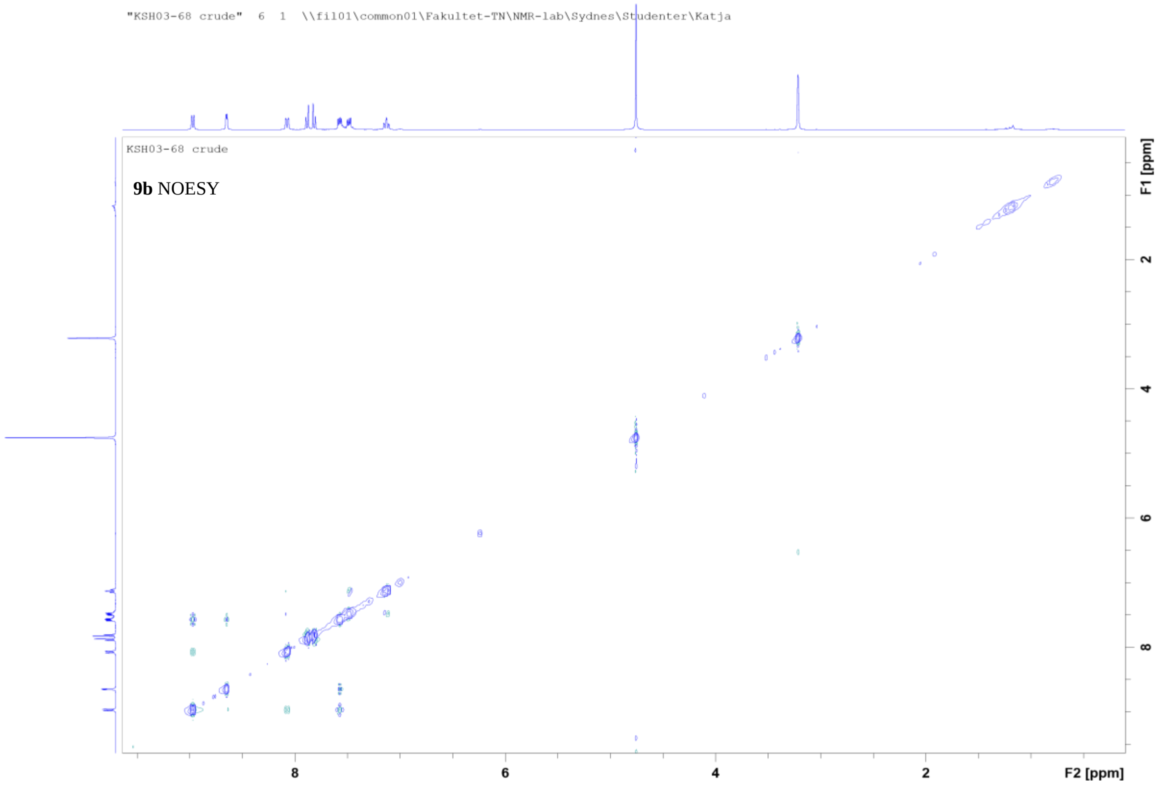
"KSH03-68 crude" 6 1 \\fil01\common01\Fakultet-TN\NMR-lab\Sydnes\Studenter\Katja
KSH03-68 crude
9b NOESY
F1 [ppm]
2
4
6
8
8
6
4
2
F2 [ppm]

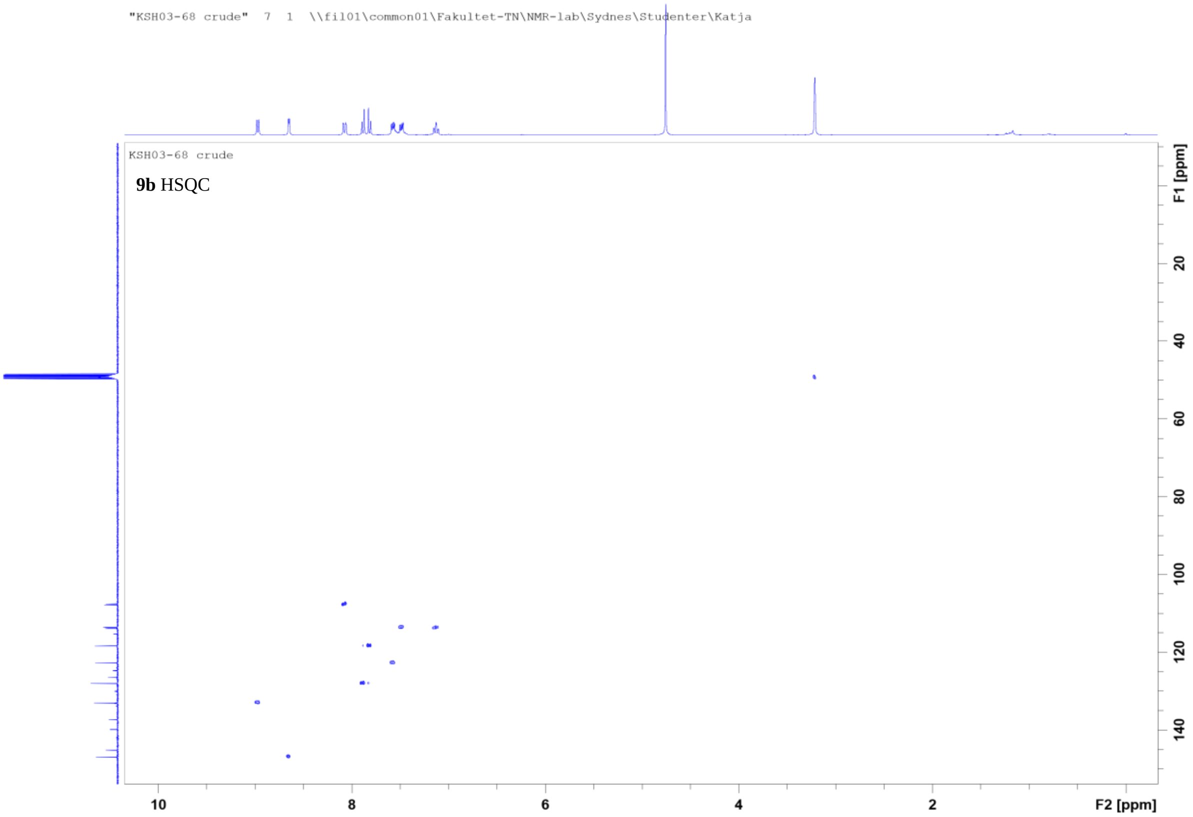
"KSH03-68 crude" 7 1 \\fil01\common01\Fakultet-TN\NMR-lab\Sydnes\Studenter\Katja
KSH03-68 crude
9b HSQC
F1 [ppm]
20
40
60
80
100
120
140
10
8
6
4
2
F2 [ppm]

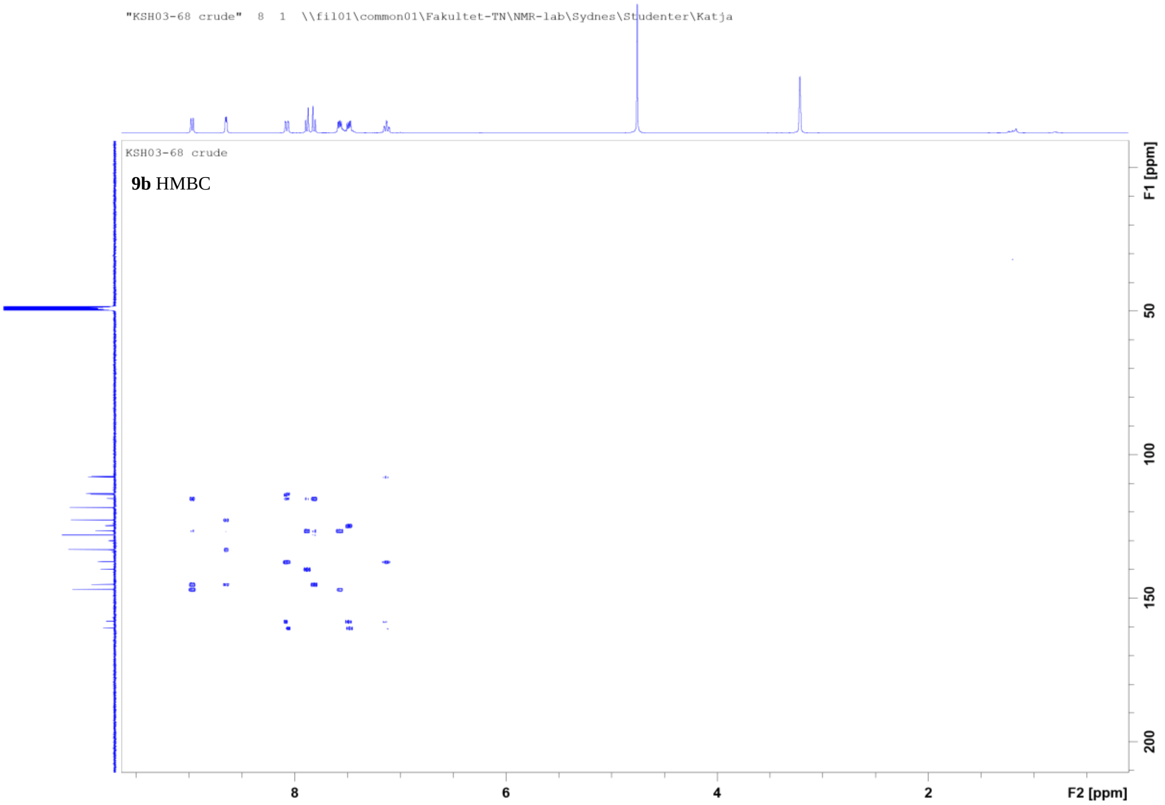
"KSH03-68 crude" 8 1 \\fil01\common01\Fakultet-TN\NMR-lab\Sydnes\Studenter\Katja
KSH03-68 crude
F1 [ppm]
50
100
150
200
8
6
4
2
F2 [ppm]

9b HMBC

4-Methyl-7*H*-pyrido[2,3-*c*]carbazolium iodide (10)

^{1}H NMR (400 MHz, DMSO-d_6)

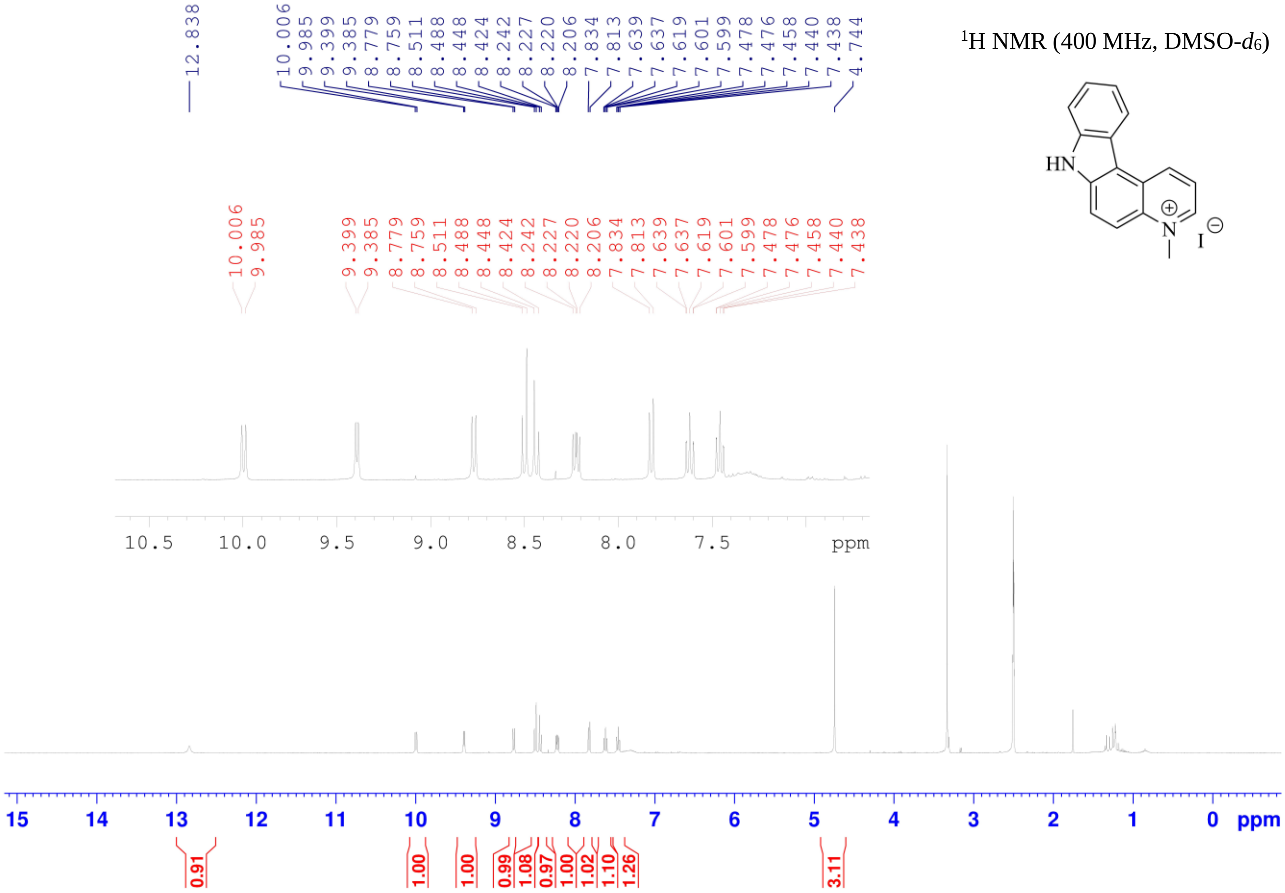

^{13}C NMR (100 MHz, DMSO-d_6)

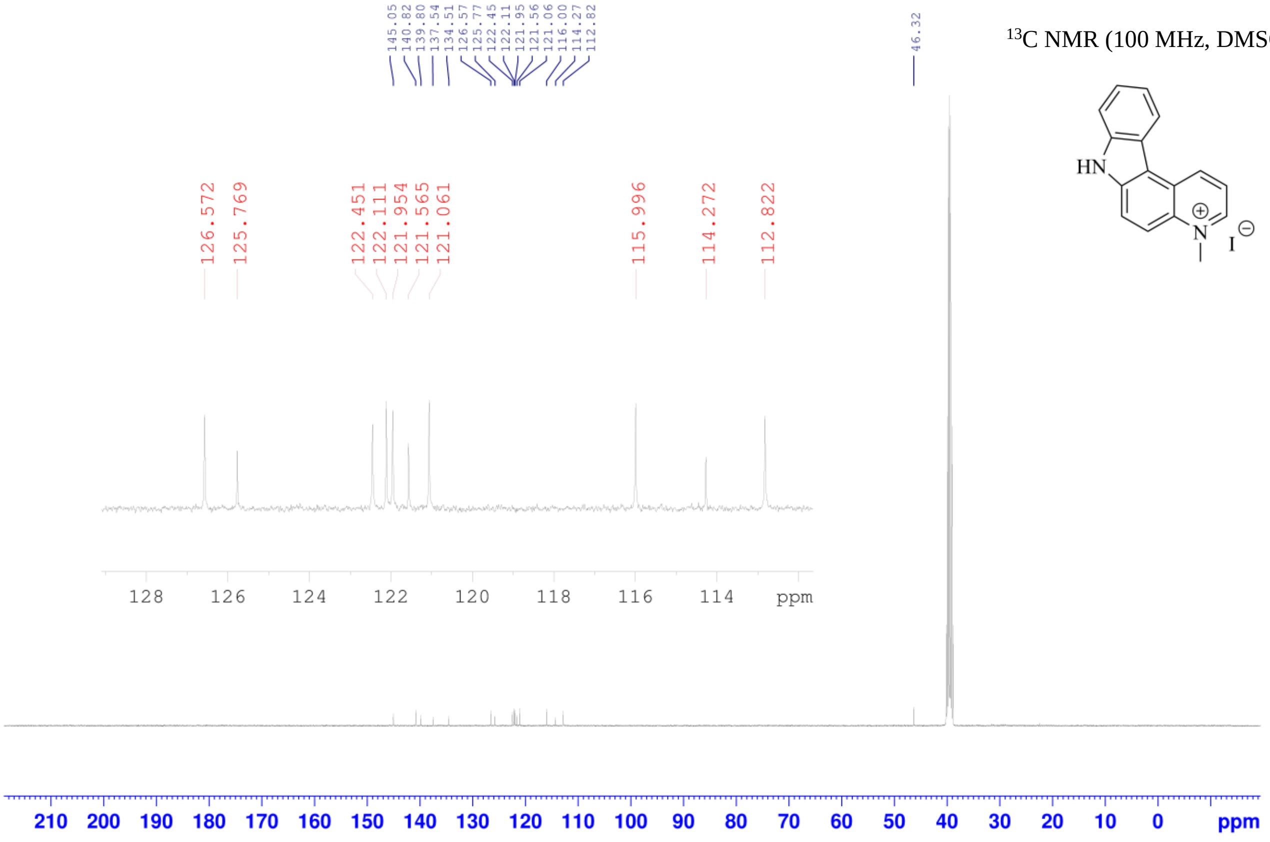

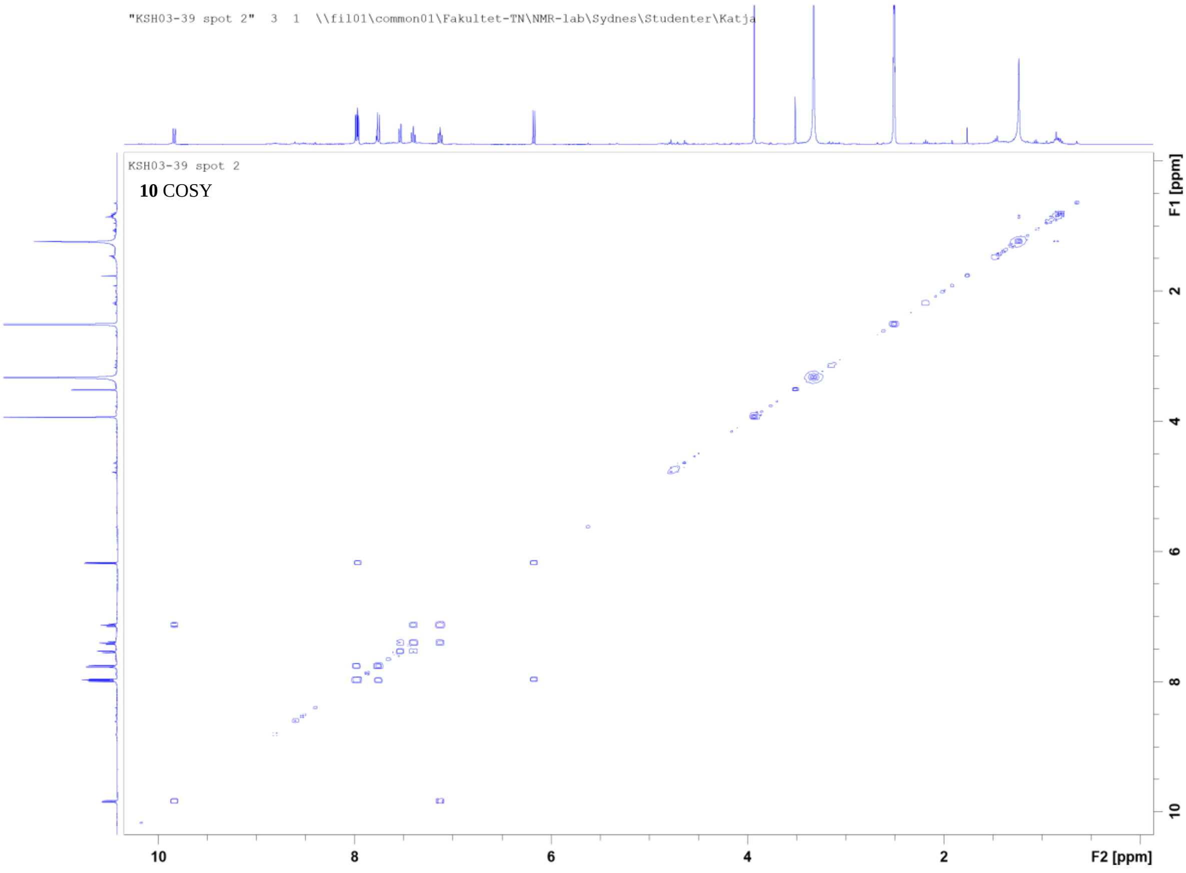

10 COSY

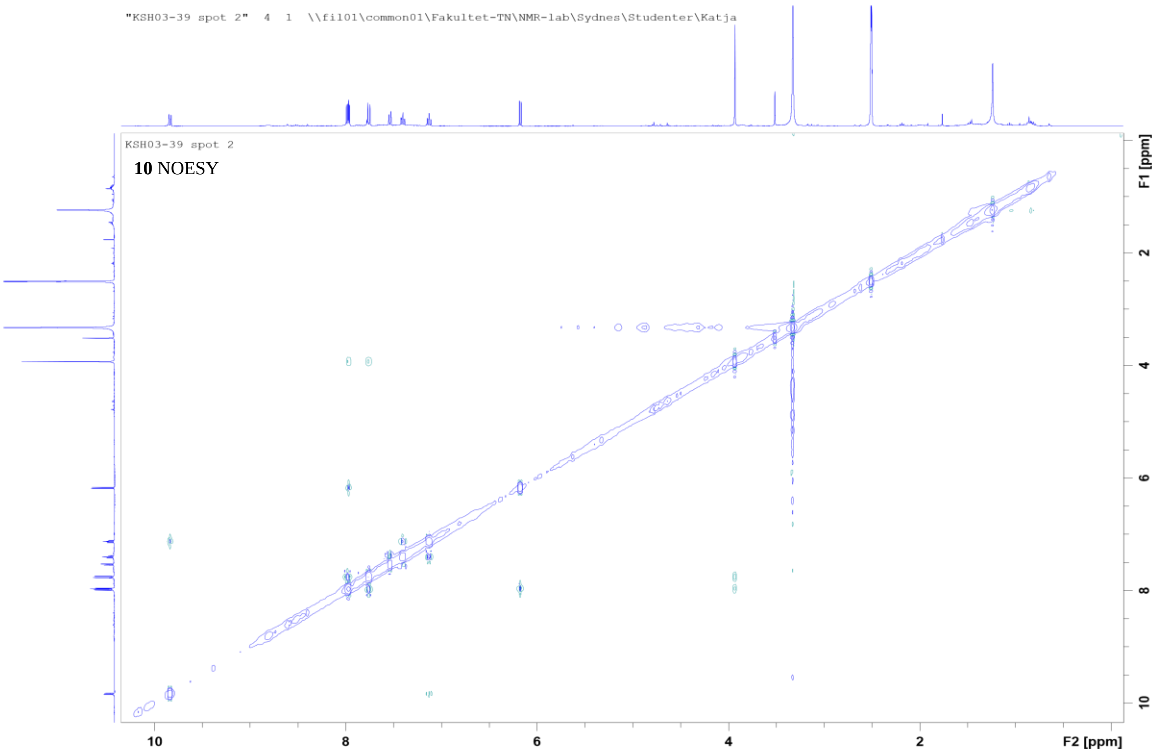
"KSH03-39 spot 2" 4 1 \\fi101\common01\Fakultet-TN\NMR-lab\Sydnes\Studenter\Katja
KSH03-39 spot 2
10 NOESY
F1 [ppm]
F2 [ppm]

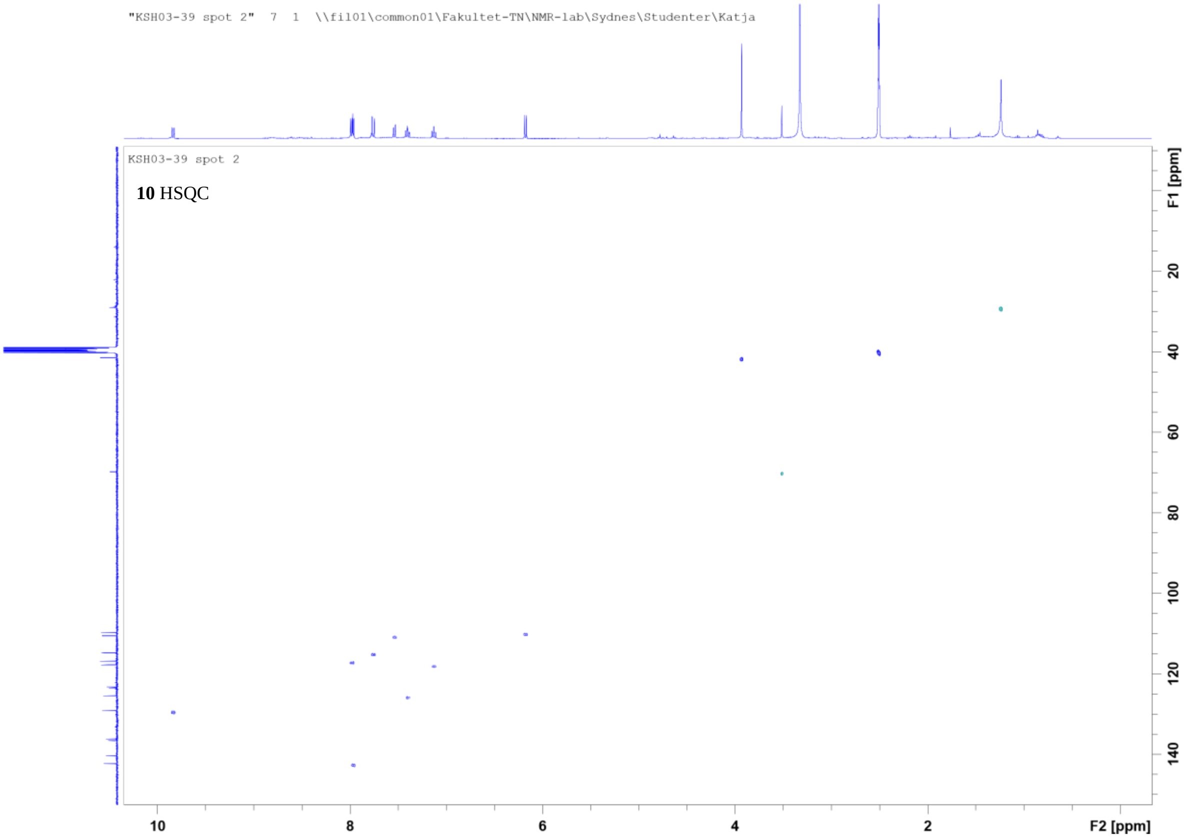
"KSH03-39 spot 2" 7 1 \\fi101\common01\Fakultet-TN\NMR-lab\Sydnes\Studenter\Katja
KSH03-39 spot 2
10 HSQC
F1 [ppm]
F2 [ppm]

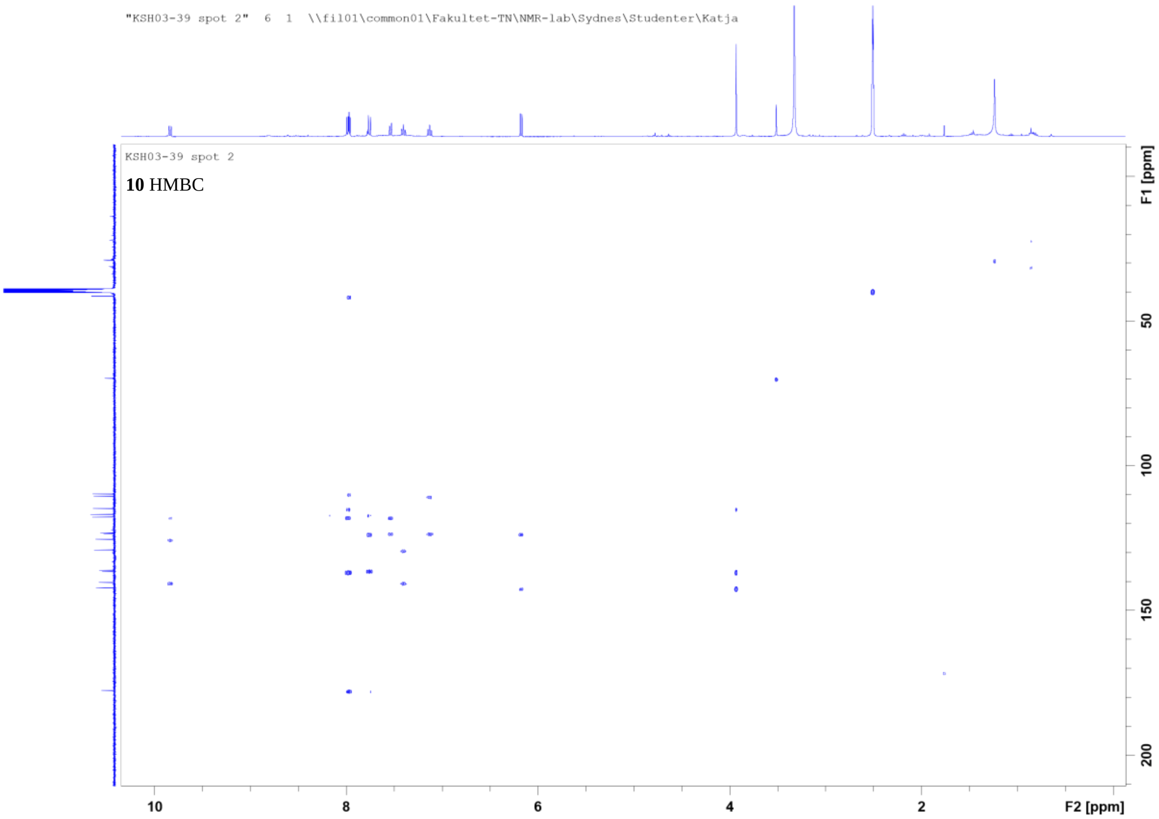
"KSH03-39 spot 2" 6 1 \\fil01\common01\Fakultet-TN\NMR-lab\Sydnes\Studenter\Katja
KSH03-39 spot 2
10 HMBC
F1 [ppm]
50
100
150
200
10
8
6
4
2
F2 [ppm]

4-Methyl-11*H*-pyrido[3,2-*a*]carbazolium iodide (21)

^{1}H NMR (400 MHz, CD_3OD)

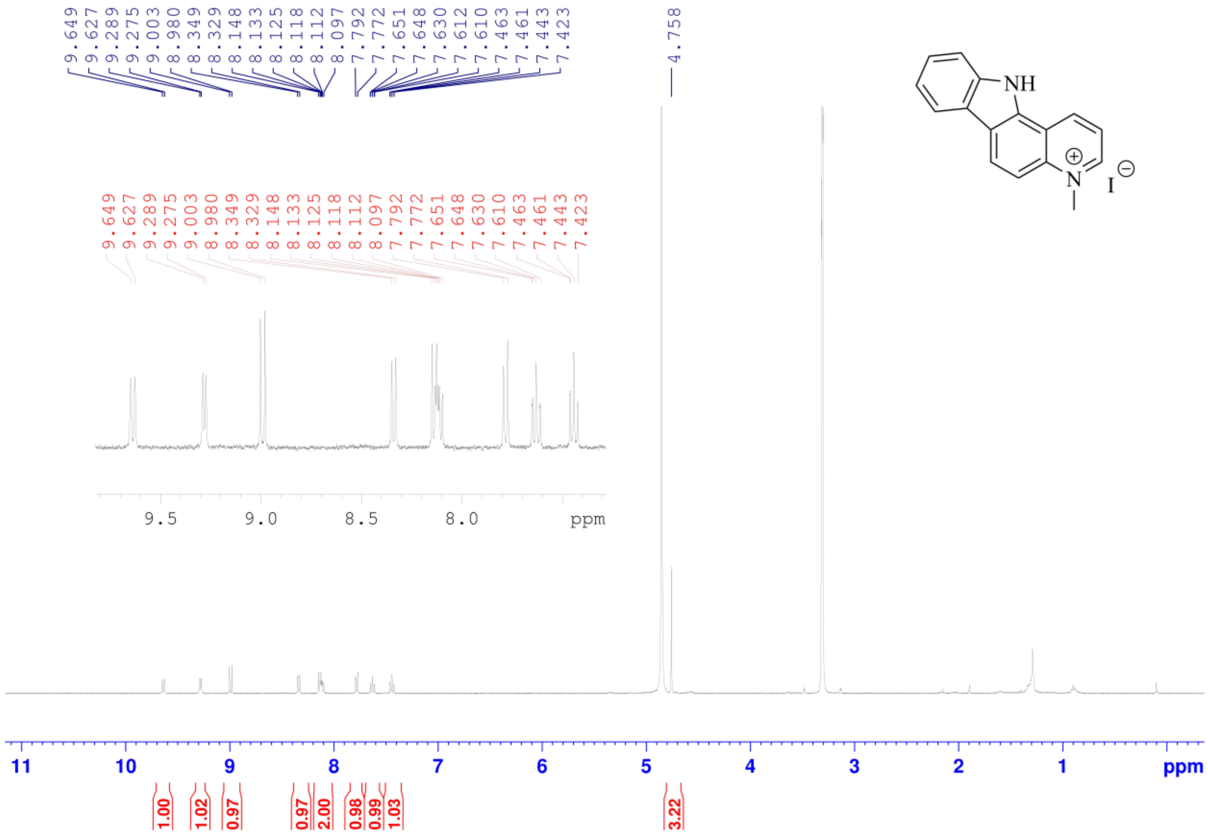

^{13}C NMR (100 MHz, CD_3OD)

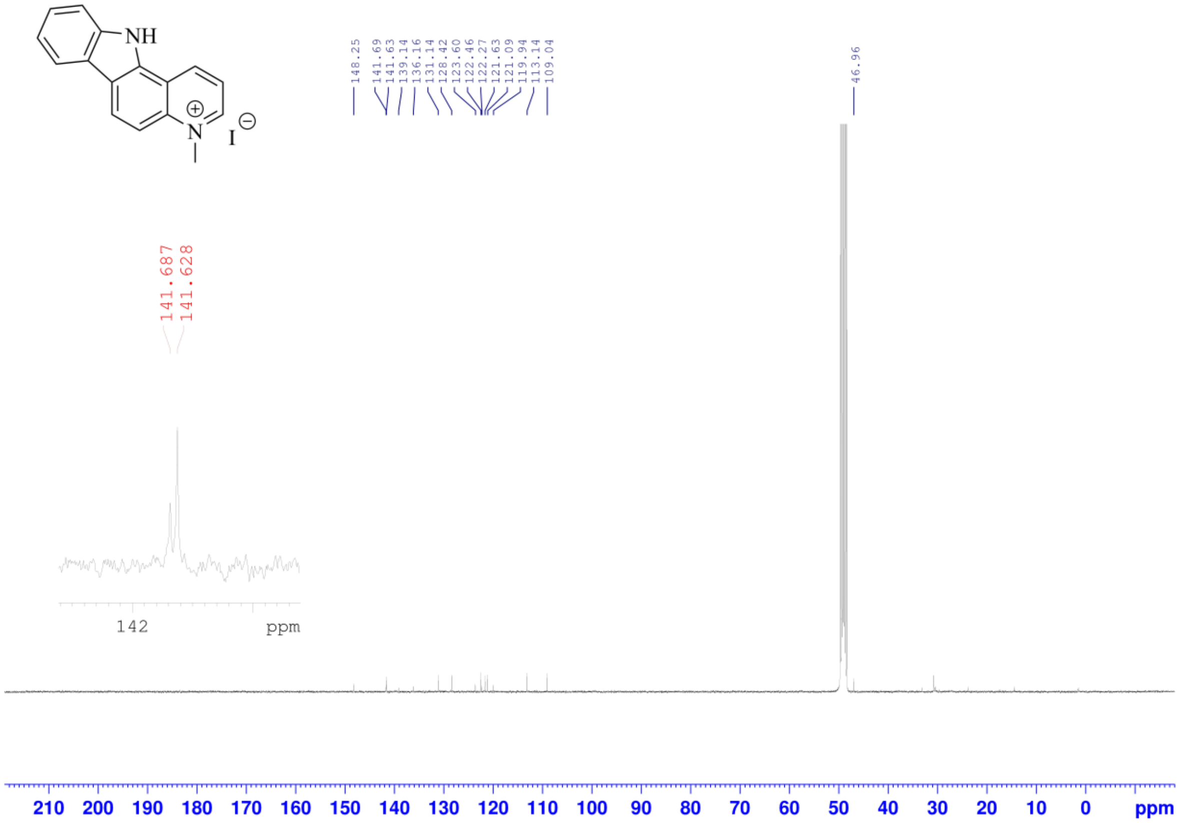

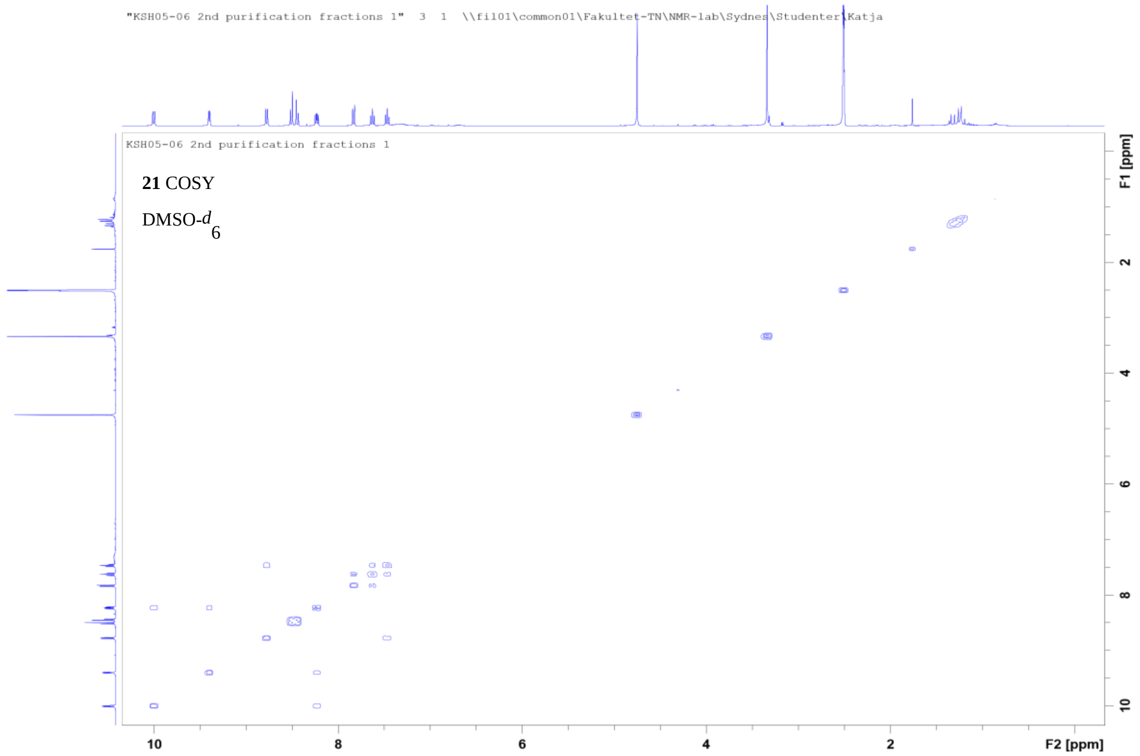

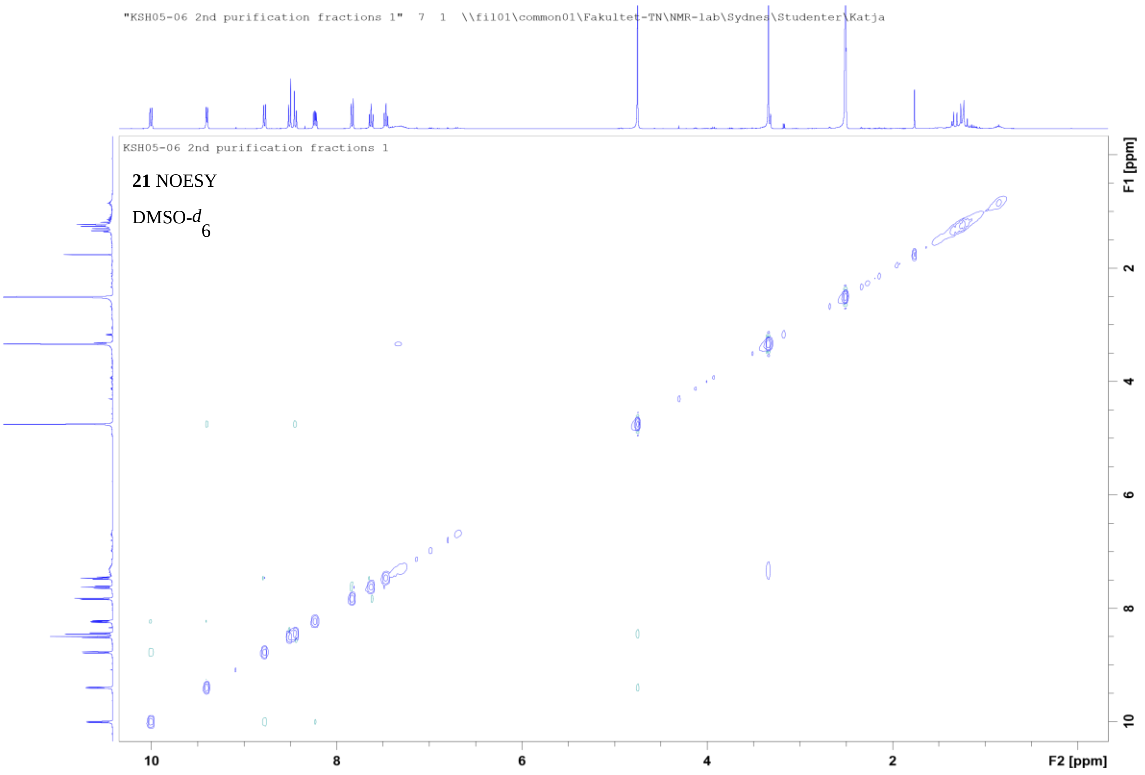
"KSH05-06 2nd purification fractions 1" 7 1 \\fil01\common01\Fakultet-TN\NMR-lab\Sydnes\Studenter\Katja
KSH05-06 2nd purification fractions 1
21 NOESY
DMSO-d6
F1 [ppm]
F2 [ppm]

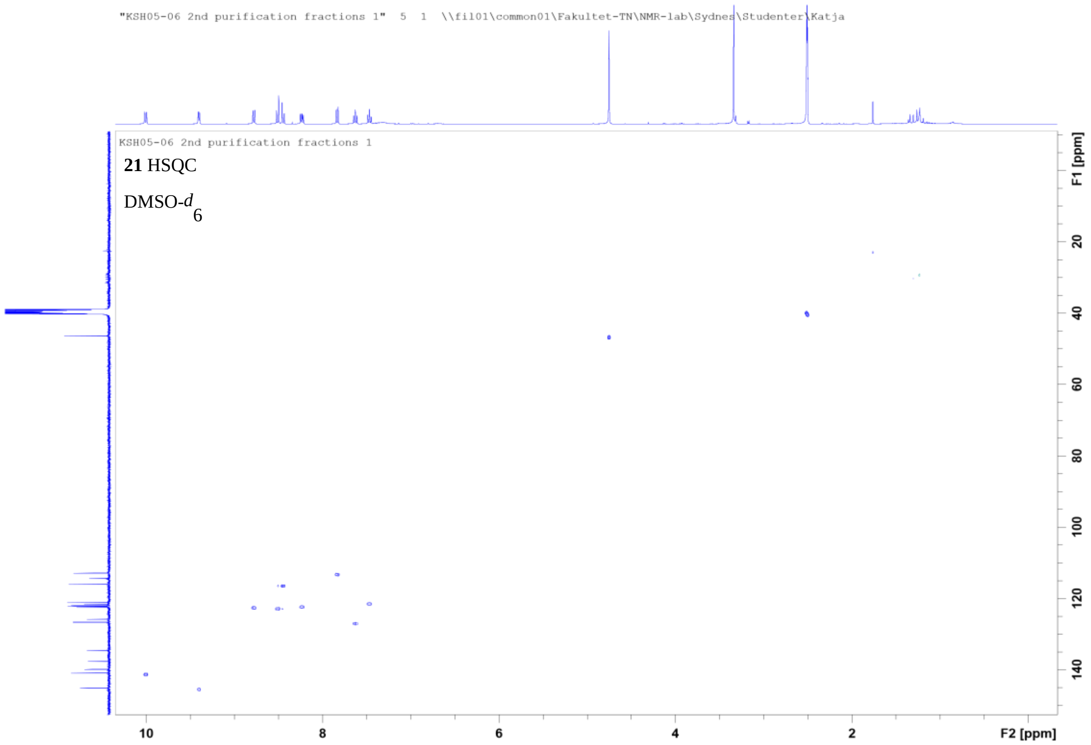
"KSH05-06 2nd purification fractions 1" 5 1 \\fil01\common01\Fakultet-TN\NMR-lab\Sydnes\Studenter\Katja
KSH05-06 2nd purification fractions 1
21 HSQC
DMSO-d6
F1 [ppm]
F2 [ppm]

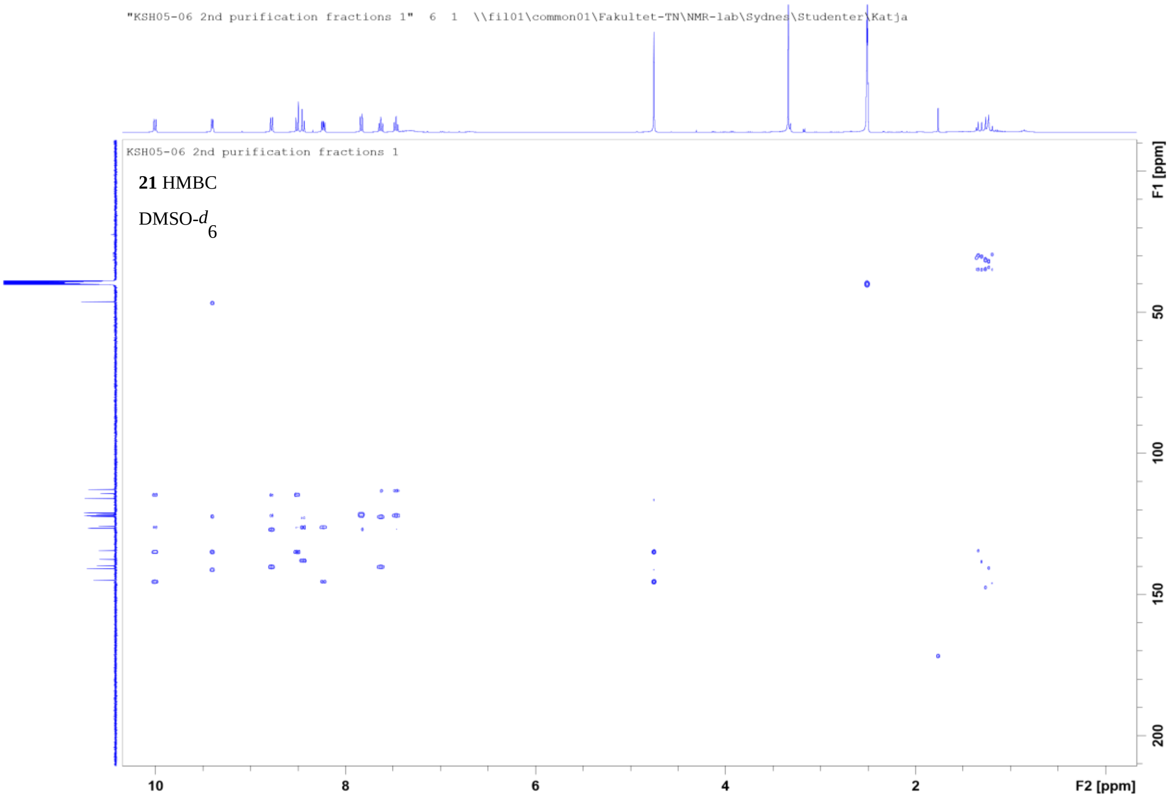

"KSH05-06 2nd purification fractions 1" 6 1 \\fil01\common01\Fakultet-TN\NMR-lab\Sydnes\Studenter\Katja
KSH05-06 2nd purification fractions 1
21 HMBC
DMSO-d6
F1 [ppm]
50
100
150
200
10
8
6
4
2
F2 [ppm]
